# Structure-guided development of heterodimer-selective GPCR ligands

**DOI:** 10.1038/ncomms12298

**Published:** 2016-07-26

**Authors:** Harald Hübner, Tamara Schellhorn, Marie Gienger, Carolin Schaab, Jonas Kaindl, Laurin Leeb, Timothy Clark, Dorothee Möller, Peter Gmeiner

**Affiliations:** 1 Department of Chemistry and Pharmacy, Emil Fischer Center, Friedrich-Alexander-Universität Erlangen-Nürnberg, Schuhstraße 19, Erlangen 91052, Germany; 2 Department of Chemistry and Pharmacy, Computer-Chemie-Center, Friedrich-Alexander-Universität Erlangen-Nürnberg, Nägelsbachstraße 25, Erlangen 91052, Germany; 3 Centre for Molecular Design, University of Portsmouth, King Henry Building, Portsmouth PO1 2DY, UK

## Abstract

Crystal structures of G protein-coupled receptor (GPCR) ligand complexes allow a rational design of novel molecular probes and drugs. Here we report the structure-guided design, chemical synthesis and biological investigations of bivalent ligands for dopamine D_2_ receptor/neurotensin NTS_1_ receptor (D_2_R/NTS_1_R) heterodimers. The compounds of types 1–3 consist of three different D_2_R pharmacophores bound to an affinity-generating lipophilic appendage, a polyethylene glycol-based linker and the NTS_1_R agonist NT(8-13). The bivalent ligands show binding affinity in the picomolar range for cells coexpressing both GPCRs and unprecedented selectivity (up to three orders of magnitude), compared with cells that only express D_2_Rs. A functional switch is observed for the bivalent ligands 3b,c inhibiting cAMP formation in cells singly expressing D_2_Rs but stimulating cAMP accumulation in D_2_R/NTS_1_R-coexpressing cells. Moreover, the newly synthesized bivalent ligands show a strong, predominantly NTS_1_R-mediated β-arrestin-2 recruitment at the D_2_R/NTS_1_R-coexpressing cells.

## Abstract (web summary)

G protein-coupled receptors (GPCRs) are involved in key signalling pathways and represent important targets for the treatment of neurological and psychiatric disorders. Here, the authors describe powerful bivalent ligands that efficiently bind to therapeutically relevant GPCR heterodimers

## Graphical abstract

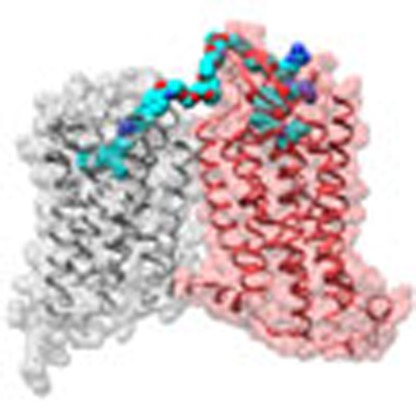

Gprotein-coupled receptors (GPCRs) form the largest family of membrane proteins^1^. Because of their diversity and critical involvement in numerous cellular signalling processes in both central nervous system (CNS) and periphery, GPCRs represent today's most popular drug targets attracting interdisciplinary scientific attention. Consequently, large progress has been made in understanding GPCR structures and modes of function. A growing number of studies showed that GPCRs not only exist as isolated entities but also interact within the plasma membrane by forming receptor dimers or higher-order oligomers^2^,^3^,^4^,^5^. Besides enabling cross-talk between individual signalling networks, receptor dimerization can induce activation of alternative signalling pathways^6^,^7^, influence ligand pharmacology and is critical for receptor trafficking and function^3^.

Dopamine D_2_ receptors (D_2_Rs), which belong to the family A of GPCRs, regulate a large number of physiological functions and are involved in a number of neuropsychiatric disorders including schizophrenia and Parkinson's disease. Along with numerous other GPCRs, D_2_Rs have been proven to form homodimers^8^,^9^ and heterodimers^10^,^11^,^12^,^13^,^14^, and growing evidence indicates that altered D_2_R cooperativity may significantly contribute to CNS disorders^15^,^16^. Among receptors interacting with D_2_Rs in the CNS, the neurotensin receptor subtype 1 (NTS_1_R) together with its endogenous ligand, the tridecapeptide neurotensin, has gained substantial interest over the past decades. Both GPCRs are closely associated and highly co-localized *in vivo*^17^. For example, more than 80% of dopaminergic neurons in the mesolimbic system express NTS_1_R (ref. 18). Moreover, neurotensin was found to decrease the D_2_R-affinity for dopamine and other agonists in striatal^19^ and co-transfected HEK 293 T membrane preparations^20^. Evidence for the physical intramembrane interaction of both receptors was also conferred by means of bioluminescence resonance energy transfer, co-immunoprecipitation and attenuation of dopaminergic signalling in co-transfected human cells^20^,^21^. Since central administration of the neuropeptide in animals can mimic the effects of neuroleptic treatment, neurotensin has been hypothesized to act as endogenous antipsychotic^22^.

Powerful tools for studying GPCR dimerization are bivalent ligands consisting of two pharmacophores tethered by an appropriate linker^23^,^24^. Bivalent ligands bridging the proximate orthosteric-binding sites of a dimer provide valuable insights into the quaternary structure of receptor dimers and the functional relevance of GPCR dimerization. Because of their selective recognition properties, bivalent ligands can be used for a tissue-specific targeting of cells expressing an individual GPCR dimer. Pioneering work in this field was performed developing dimer-preferring ligands to investigate opioid receptor dimerization *in vitro* and *in vivo*^25^,^26^,^27^ and further compounds were synthesized to target GPCR homo- and heterodimers^28^,^29^,^30^,^31^,^32^,^33^. In theory, bivalent ligands successfully bridging two binding sites of adjacent protomers should confer extremely high affinity (resulting from the total binding energy of two recognition elements) and thus selectivity for the heterodimer. Most of the previous reports have shown compounds with only modest preference for heterodimers over monomers.

High-resolution crystal structures of GPCR-ligand complexes open new opportunities for the design of bivalent ligands. A carefully designed bivalent ligand bridging two neighboured receptor protomers should exhibit extremely high binding affinity. This approach should lead to high tissue selectivity between heterodimer-expressing cells and those that express only one individual receptor^6^. Our work presents heterobivalent D_2_R/NTS_1_R ligands of type 1-3 comprising NT(8-13), the active fragment of the neuropeptide neurotensin, covalently linked to three different D_2_R-specific pharmacophores. These newly synthesized bivalent compounds exhibit high selectivity up to three orders of magnitude and picomolar *K*_i_ values in D_2_R/NTS_1_R-coexpressing cells compared with cells expressing D_2_R only. Using bivalent ligands containing an agonist D_2_R pharmacophore substructure, we demonstrate that G_i_/G_o_-promoted D_2_R signalling is attenuated in the D_2_R/NTS_1_R coexpressing cells, while the compounds behave as full dopamine receptor agonists in cells singly expressing D_2_R.

## Results

### Design

**Figure 1:**
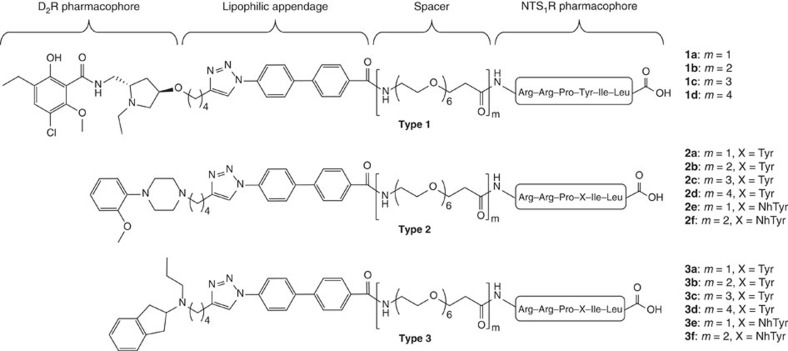
Newly synthesized bivalent ligands. Type 1-3 ligands contain NT(8-13) as NTS_1_R pharmacophore and differ in their D_2_R recognition element (type 1: eticlopride, antagonist; type 2: 2-methoxyphenylpiperazine, antagonist; and type 3: aminoindane, agonist). The spacer length connecting both pharmacophores ranges from 22 to 88 atoms (*m*=1–4). For bivalent control compounds **2e**/**f** and **3e**/**f**, tyrosine was replaced by N-homotyrosine (NhTyr).

**Figure 2:**
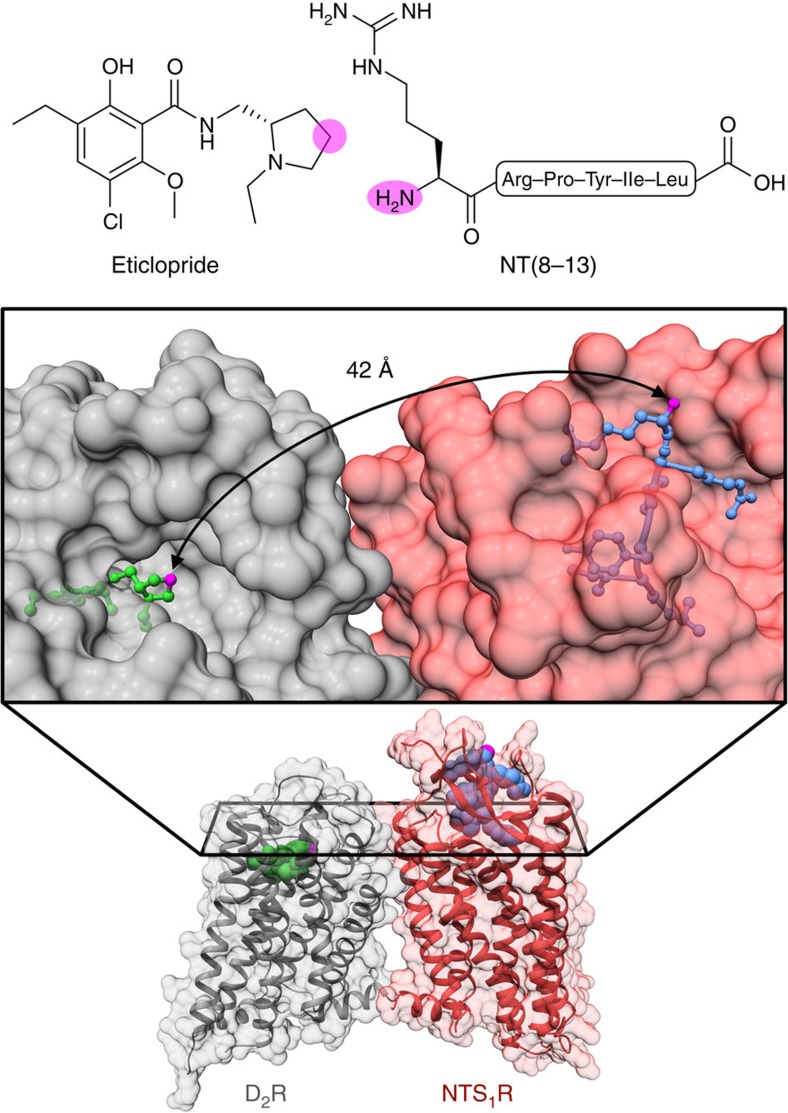
A structure-guided approach for the design of bivalent ligands. On the basis of X-ray crystal structures, a D_2_R/NTS_1_R heterodimer model was generated and exploited for the design of bivalent ligands. A side view of dimer model is displayed at the bottom half and the ligand structures at the top of the figure. The middle part shows a magnified top view of the dimer model. Ribbons and surfaces of D_2_R and NTS_1_R are coloured in grey and red, respectively. Eticlopride (green) and NT(8-13) (blue) were positioned according to their coordinates in the crystal structures of D_3_R and NTS_1_R, respectively. Clearly, the N-terminus of NT(8-13) and the 4'-position of eticlopride are accessible from the extracellular side and were therefore selected as attachment points (highlighted as pink spots in all three representations). The beeline and hence the minimum distance connecting these two attachment points measures 42 Å.

To design heterobivalent ligands, we intended to connect three different D_2_R pharmacophores to the NTS_1_R agonist NT(8-13) via an affinity-generating biphenyltriazole-moiety (lipophilic appendage)^34^,^35^ and ω-amino acid-functionalized polyethylene glycol (PEG) spacers (Fig. 1). As D_2_R pharmacophores, we used the D_2_R/D_3_R antagonist eticlopride, co-crystallized in complex with D_3_R (ref. 36), the privileged structure of a phenylpiperazine-based scaffold^37^ and an aminoindane-type agonist^32^. Suitable attachment points for the connection of the pharmacophores with the linker were identified using the crystal structures of NTS_1_R (refs 38, 39) and D_3_R (ref. 36). Inspection of the crystal structures revealed that the N-terminus of NT(8-13) and the 4'-position of eticlopride are accessible from the extracellular side. In an effort to determine a suitable linker length, we generated a D_2_R/NTS_1_R heterodimer model (Fig. 2) consisting of a D_2_R homology model^40^ (which was based on the D_3_R crystal structure) and the NTS_1_R crystal structure^39^ (Supplementary Note 1; Supplementary Tables 1,2; and Supplementary Fig. 1). As templates to build the dimer model, we considered 16 crystal structures of 12 different GPCRs displaying homodimers with 18 individual receptor orientations. We generated dimer models based on every template. Models were not considered further if they showed substantial clashes between the two receptors, as well as models revealing a high distance between the protomers or showing a low parallelism of the two protomers. Showing relatively high sequence similarity with D_2_R, the structure of a β_1_-adrenergic receptor (β_1_-AR) homodimer^41^ was selected as a template for the generation of the heterodimer model. The crystal structure revealed a dimer interface involving transmembrane helix 1 (TM1), TM2 and helix 8 (H8) that was previously reported to be important for D_2_R dimerization^8^ and validated by crosslinking studies at β1-AR (ref. 41). The model showed a minimum distance (beeline) of 42 Å between the attachment points of eticlopride and NT(8-13) (Fig. 2 and Supplementary Note 2). However, docking of eticlopride with the affinity-generating biphenyltriazole-moiety into the heterodimer model revealed two reasons why a longer spacer length should be required. First, the binding pocket of D_2_R restricts the D_2_R-attachment in a position not facing straight towards NTS_1_R (Supplementary Fig. 2a) and in addition, the way is partially blocked by the extracellular loop 1 of D_2_R and the N-terminus of NTS_1_R, resulting in a total distance of ∼55 Å. We concluded that at least two PEG-units, in addition to the biphenyltriazole-based attachment, should be necessary to enable a bivalent-binding mode (ligand **1b**), while a ligand containing only one PEG-unit (ligand **1a**, corresponding to a maximal linker length of ∼46 Å) should lack the ability to bridge the two binding sites and could thus serve as a control agent (Supplementary Fig. 2b). To determine an optimum linker length, we additionally designed compounds **1c** and **1d** bearing three and four units of the functionalized PEG-spacer, respectively. Using an identical approach, we designed the bivalent compounds of types 2 and 3 featuring phenylpiperazine- and aminoindane-based D_2_R pharmacophores. Here, the attachment points at the pharmacophores were identified based on docking studies (Supplementary Note 2 and Supplementary Fig. 3). We performed molecular dynamics (MD) on the bivalent ligands **1b**, **2b** and **3b** (400 ns for each compound) in complex with the generated heterodimer model, because flaws in the process of ligand design would potentially appear as instabilities in the simulation systems. All three systems adopted stable receptor–ligand complexes during this time (Supplementary Note 3; Supplementary Fig. 4; and Supplementary Data 1, 2, 3).

### Synthesis

Chemical synthesis was conducted on solid phase involving the generation of the peptidic sequence, followed by ligation of the individual linker and coupling with the carboxylate-functionalized dopaminergic pharmacophore. The affinity-generating biphenyltriazole-moieties were installed using click chemistry. To generate appropriate control agents, we linked the dopaminergic building blocks to a peptide–peptoid hybrid of NT(8-13) (ref. 42), which is highly similar to NT(8-13) but shows only poor NTS_1_R affinity (compounds **2e**,**f**, **3e**,**f**; Fig. 1 and Supplementary Table 3).

### Radio-ligand binding

**Figure 3:**
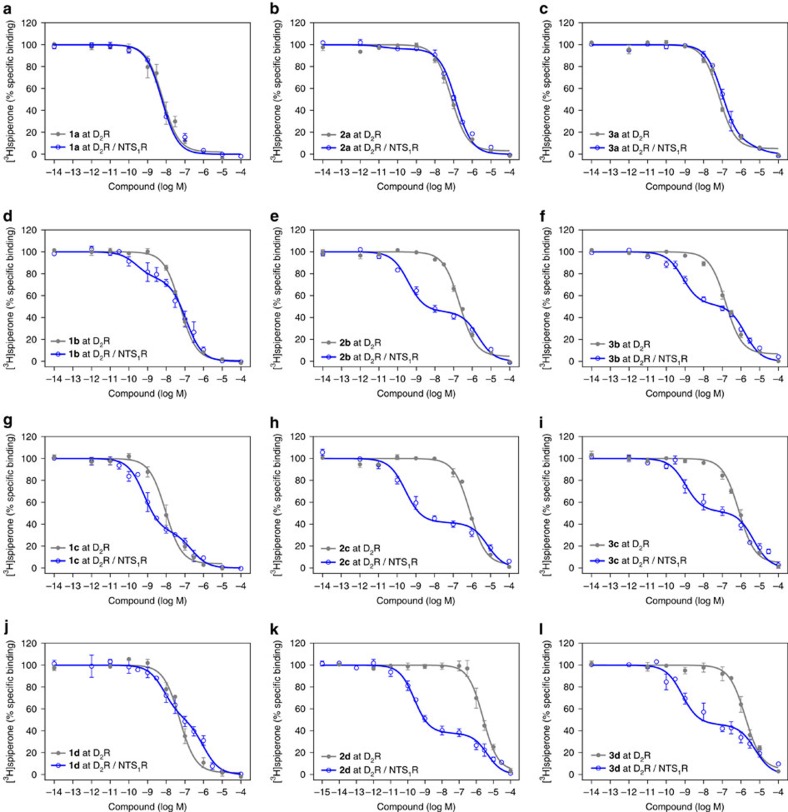
Biphasic competition-binding curves indicate a bivalent binding mode. Dopamine receptor binding of the bivalent ligands **1a**–**d**, **2a**–**d** and **3a**–**d** was measured by displacement of the radio-ligand [^3^H]spiperone from membranes of HEK 293 T cells coexpressing D_2_R/NTS_1_R (blue open circles) or monoexpressing D_2_R only (grey filled circles). (**a**-**c**) Bivalent ligands with a spacer length of 22 atoms (*m*=1) result in monophasic competition-binding curves. (**d**–**l**) Biphasic-binding curves indicating bivalent ligand binding are observed when the linker length is increased to 44, 66 or 88 atoms (*m*=2–4) at membranes coexpressing both target receptors (D_2_R/NTS_1_R) but not at membranes with D_2_R only. Data points represent the mean±s.e.m. of 3–22 independent experiments (see Table 1 for details), each performed in triplicate.

Binding profiles of the bivalent ligands of types 1–3 were determined by displacement of the radio-ligand [^3^H]spiperone from the human D_2_R in membranes from HEK 293 T cells singly expressing the D_2_R and in D_2_R/NTS_1_R-coexpressing cells (Table 1). Test compounds **1a**, **2a** and **3a** containing biphenyltriazole-substituted eticlopride, phenylpiperazine and aminoindane moieties, respectively, linked to NT(8-13) by a short 22-atom spacer to NT(8-13) showed acceptable binding affinities to the D_2_R with *K*_i_ values ranging from 1.4 and 1.7 nM for **1a** up to double-digit nanomolar values for **2a** and **3a**, in D_2_R and D_2_R/NTS_1_R expressing membranes (Table 1 and Fig. 3a–c). Extension of the linker to 44 atoms resulted in comparable affinities for **1b**, **2b** and **3b** at the D_2_R monoexpressing cells, (*K*_i_ 9.9, 42 and 36 nM). However, binding characteristics at the D_2_R/NTS_1_R-coexpressing cells were changed dramatically by the elongation of the linker. Thus, we observed biphasic competition curves with two individual values for *K*_i high_ and *K*_i low_ (Fig. 3d–f). For all three compounds, high-affinity binding was observed at subnanomolar concentrations (*K*_i high_ 0.11–0.47 nM) with a high-affinity population of 31–55%, while the affinity for the low-affinity site ranged from 43 to 630 nM (Table 1). We suggest that the high-affinity *K*_i_ values represent a bivalent receptor-bridging binding mode of **1b**, **2b** and **3b** to D_2_R/NTS_1_R heterodimers, whereas low-affinity *K*_i_ values reflect a monovalent-binding mode to D_2_R as a monomer or within a homo-/heterodimer. Thus, these newly designed ligands exhibit a 76–200-fold preference for the high-affinity bivalent interaction with the D_2_R/NTS_1_R heterodimer over monovalent-binding modes to D_2_R monoexpressing membranes.

In contrast, the respective analogues **2e**/**f** and **3e**/**f** (spacer length 22 and 44 atoms), containing a peptide–peptoid hybrid with almost no affinity for NTS_1_R instead of the highly similar peptide NT(8-13), displayed monophasic-binding curves at both, D_2_R and D_2_R/NTS_1_R-coexpressing membranes (*K*_i_ 15-40 nM for D_2_R and *K*_i_ 22–110 nM for D_2_R/NTS_1_R; Supplementary Fig. 5a–d). Typical monophasic-binding curves were also observed for the monovalent analogues of types 2 and 3 ligands **2g** and **3g** for both expression systems (*K*_i_ 20 and 21 nM for **2g** and **3g** at D_2_R and *K*_i_ 42 and 42 nM for **2g** and **3g** at D_2_R/NTS_1_R, respectively; Supplementary Fig. 5e,f).

In an attempt to find an optimum linker length, we also investigated the binding behaviour of compounds **1c**–**3c** and **1d**–**3d** with a spacer length of 66 and 88 atoms, respectively. Whereas ligands **1c** and **1d** maintained one-digit nanomolar affinity (*K*_i_ 2.5 and 9.0 nM), elongation of the spacer led to a loss of binding affinity for types 2 and 3 ligands (*K*_i_ 140–520 nM) at D_2_R monoexpressing membranes. Nevertheless, the biphasic-binding profiles with separated high- and low-affinity sites at D_2_R/NTS_1_R-coexpressing membranes were preserved or even enhanced for ligands **1c**–**3c** and **1d**–**3d** (*K*_i high_ 0.087–2.6 nM, *K*_i low_ 120–1,800 nM, Fig. 3g–l). Within the entire set of compounds, ligand **2d** (spacer length 88 atoms) displayed the outstanding affinity of 87 pM for the high-affinity binding site. Interestingly, the preference for the high-affinity binding site versus the affinity for D_2_R monoexpressing membranes was more pronounced for types 2 and 3 ligands based on phenylpiperazines or aminoindane as D_2_R pharmacophores (76–4,700-fold), compared with the eticlopride-based derivatives (3.5–90-fold), with comparable fractions of high-affinity binding sites (50–66% high-affinity fraction).

**Figure 4:**
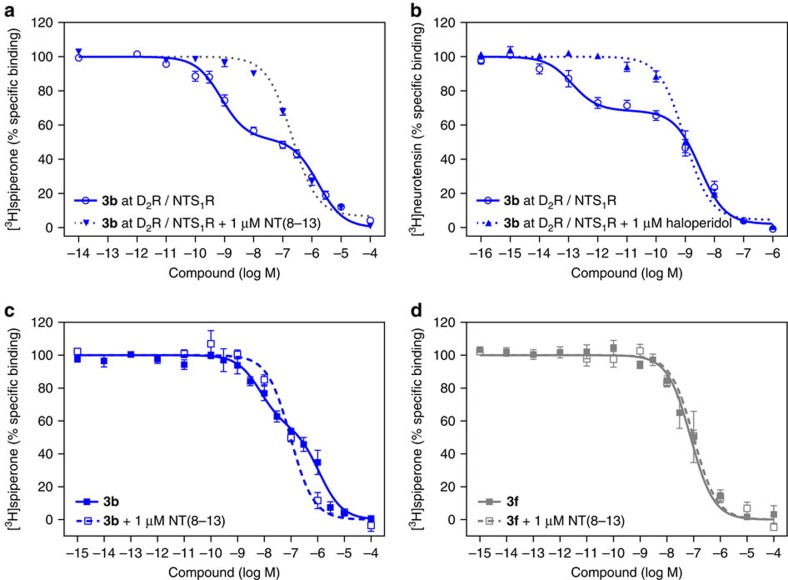
Prevention of bivalent binding mode abolishes biphasic competition curves. (**a**) Dopamine receptor binding of **3b** (*m*=2, 44-atom spacer) at D_2_R/NTS_1_R in the absence (blue open circles) or presence (blue inverted triangles) of 1 μM NT(8-13). Incubation with the monovalent NTS_1_R agonist NT(8-13) prevents a bivalent binding mode and converts the biphasic-binding curve (*K*_i high_ 0.47±0.14 nM, *K*_i low_ 300±40 nM, *n*=22) into a monophasic sigmoid competition curve (*K*_i_ 63±8 nM, *n*=6). (**b**) Neurotensin receptor binding of **3b** (*m*=2) at D_2_R/NTS_1_R in the absence (blue open circles) or presence (blue filled triangles) of 1 μM haloperidol. Incubation with the monovalent D_2_R antagonist prevents the bivalent binding mode, observed for the coexpression of D_2_R/NTS_1_R (*K*_i high_ 0.11±0.05 pM, *K*_i low_ 3.7±1.4 nM, *n*=9 versus *K*_i_ 0.79±0.21 nM, *n*=9). (**c**) When radio-ligand displacement studies were performed with striatal membranes and [^3^H]spiperone, biphasic-binding behaviour was observed for the bivalent ligand **3b** (blue filled squares, *K*_i high_ 2.8±1.1 nM, *K*_i low_ 310±90 nM, fraction high-affinity sites 38±5%, *n*=5) alone, but not in the presence of 1 μM NT(8-13) (blue open sqares, *K*_i_ 28±3 nM, *n*=3). (**d**) For the bivalent control compound **3f** comprising a peptoid-peptide hybrid instead of the NT(8-13) pharmacophore monophasic competition curves were observed in the absence (grey filled squares, *K*_i_ 28±16 nM, *n*=5) and presence (grey open squares, *K*_i_ 29±5 nM, *n*=4) of 1 μM NT(8-13). Data represent mean±s.e.m. of *n* independent experiments, each performed in triplicate.

Additional binding assays were performed in the presence of an excess of NT(8-13) (1 μM), which should prevent a bivalent-binding mode of the test compounds to D_2_R/NTS_1_R-coexpressing membranes by displacing the NT(8-13) pharmacophore of the bivalent ligands from NTS_1_R. In fact, co-incubation prevented high-affinity binding, resulting in typical sigmoidal monophasic curves (Fig. 4a and Supplementary Fig. 6a). Detailed analyses revealed a slightly reduced D_2_R-affinity in the presence of the monovalent NTS_1_R agonist, which is in agreement with the reduced *K*_i low_ observed for bivalent ligands at D_2_R/NTS_1_R-coexpressing membranes compared with D_2_R monoexpression (Table 1). Importantly, these findings are consistent with earlier studies demonstrating a negative effect of neurotensin especially on D_2_R agonist affinity^19^,^20^. The binding properties of the reference antagonist spiperone remained almost constant under these conditions (*K*_i_ 0.073 and 0.080 nM, *n*=3, in the absence and presence of 1 μM NT(8-13), respectively).

However, even in the absence of NT(8-13), slight differences between the affinity for D_2_R monoexpressing membranes and the low-affinity binding site of D_2_R/NTS_1_R-coexpressing membranes were observed, suggesting that more complex ligand/receptor interactions might take place in the coexpressing membranes. Moreover, the simultaneous presence of at least three binding modes (bivalent and monovalent to D_2_R/NTS_1_R heterodimer, monovalent to D_2_R monomer) should putatively result in triphasic-binding curves, which we have not been able to resolve.

Above described experiments were performed with a twofold excess of NTS_1_R, concluding that most of [^3^H]spiperone-bound D_2_R were able to form D_2_R/NTS_1_R heterodimers. By changing the ratio towards a two- or three-fold excess of D_2_R, the high-affinity fraction, which corresponds to the bound receptor heterodimer, was rightward shifted and the biphasic character of the curve was gradually diminished (Supplementary Fig. 6b). Thus, a correlation between the ratio of protomers and the formation of molecular entities bound by bivalent ligands with particularly high affinity could be demonstrated.

Further competition experiments with the bivalent ligands **2b** and **3b** were conducted in presence of the non-hydrolysable GTP analogue GppNHp, thereby destabilizing receptor-G protein association. In fact, co-incubation with 100 μM GppNHp had no influence on the binding behaviour of the bivalent ligand **2b** at membranes from D_2_R-expressing cells (*K*_i – GppNHp_ 42±5 nM versus *K*_i+GppNHp_ 45±6 nM). In contrast, a slight rightward shift of the *K*_i_ was observed for compound **3b**, which is in good agreement with its D_2_R agonist pharmacophore (*K*_i – GppNHp_ 36±9 nM versus *K*_i+GppNHp_ 68±8 nM). However, at D_2_R/NTS_1_R-coexpressing membranes, a rightward shift of the high-affinity binding site occurred for both compounds (5.2- and 6.4-fold for **2b** and **3b**, respectively, Supplementary Fig. 7a,b). These changes are expected, since the agonist NT(8-13) as NTS_1_R-recognizing fragment is part of both bivalent ligands, and agonist affinity is strongly dependent on the presence of G proteins. Nonetheless, in the presence of GppNHp, the biphasic-binding behaviour is retained, leading to a 330- and 190-fold preference for the high- over the low-affinity binding site in D_2_R/NTS_1_R-coexpressing membranes.

To confirm the bivalent receptor-bridging binding mode, we performed reciprocal competition experiments by labelling the NTS_1_R with the radio-ligand [^3^H]neurotensin. Therefore, we used a homogenate with a 2.5-fold excess of D_2_R. Employing **3b**, we observed a biphasic-binding curve in cells expressing the D_2_R/NTS_1_R heterodimer with a *K*_i high_ value of 0.11 pM and a *K*_i low_ at 1.7 nM, which was shifted to a monophasic sigmoidal binding curve in the presence of haloperidol (*K*_i_ 0.79 nM, Fig. 4b). Hence, incubation with the monovalent D_2_R antagonist efficiently prevented the bivalent-binding mode. Affinities for this competition-enforced monovalent-binding mode were found to be in good agreement with results obtained with membranes from CHO-cells stably expressing NTS_1_R only (*K*_i_ 0.86 nM; Supplementary Table 3 and Supplementary Methods).

To complement the results obtained with overexpressing heterologous cell lines with results from native brain tissue, competition-binding studies with [^3^H]spiperone and the bivalent ligand **3b** in comparison with the control agent **3f** (both with a spacer length of 44 atoms) were performed with membranes from porcine striatum. Convincingly, test compound **3b** displayed a biphasic-binding behaviour with a 140-fold preference for the high-affinity binding site over the low-affinity receptor population (*K*_i high_ 2.8 nM, *K*_i low_ 310 nM, high-affinity fraction 38%). In good agreement with the results from heterologous cell lines, addition of 1 μM NT(8-13) reverted this biphasic-binding curve to a sigmoidal binding isotherm with a *K*_i_ value of 28 nM. In contrast, typical monophasic-binding curves with *K*_i_ values of 28 and 29 nM were observed in the absence and presence of 1 μM NT(8-13) for the highly similar peptide–peptoid hybrid ligand **3f**, which proved to have almost no affinity for NTS_1_R (Fig. 4c,d). Thus, the connection of D_2_R- and NTS_1_R-addressing pharmacophores by an appropriate linker allows the superior recognition of heterodimers over monomers or homo(oligo-)mers not only in heterologous cell lines but also in native tissue. However, the observed preferences are less pronounced in striatal membranes, which might be, at least in part, explained by lower receptor expression levels leading to a lower propensity to form D_2_R/NTS_1_R heterodimers.

### Functional evaluation

**Figure 5:**
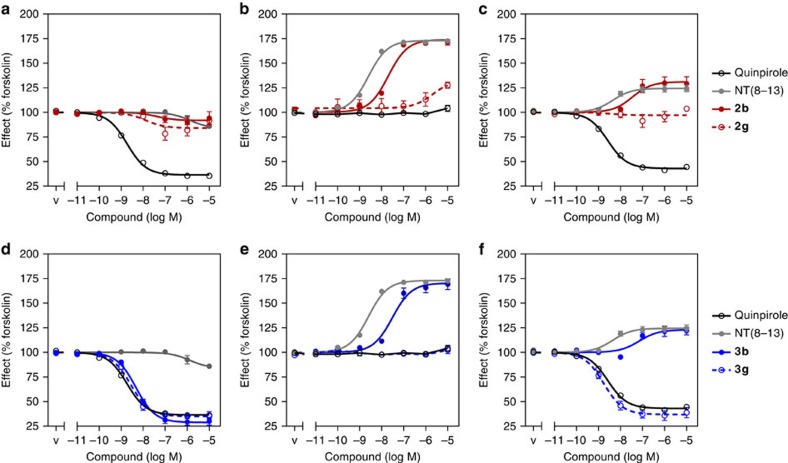
Functional investigation (cAMP accumulation) for representative ligands. Functional activity of the bivalent ligands **2b**, **3b** and their monovalent analogues **2g**, **3g** was determined in HEK 293 T cells coexpressing the cAMP-BRET biosensor CAMYEL and the D_2_R (**a**,**d**), the NTS_1_R (**b**,**e**) or both D_2_R and NTS_1_R (**c**,**f**). Cells were stimulated with increasing amounts of the ligands in the presence of 10 μM forskolin. cAMP production was normalized to the percentage of forskolin-induced cAMP concentration (100%). (**a**) While quinpirole potently inhibited cAMP formation, both D_2_R antagonists **2b**, **2g** and the NTS_1_R agonist NT(8-13) remained without significant effect on cells expressing D_2_R. (**b**) NTS_1_R could be stimulated by NT(8-13) and the bivalent ligand **2b**, also bearing a NT(8-13) pharmacophore. (**c**) In the coexpressing cells, NT(8-13) induced an increase of cAMP, while quinpirole decreased the forskolin stimulated cAMP production. The bivalent ligand **2b** also increased the cAMP production, similar to cells expressing NTS_1_R only. (**d**) Ligands **3b** and **3g** inhibited cAMP formation highly similar to quinpirole, revealing potent D_2_R agonism. (**e**) In cells monoexpressing NTS_1_R, only the bivalent ligand **3b** stimulated receptor activation, while the monovalent analogue **3g** caused no effect. (**f**) While the monovalent ligand **3g** showed an effect similar to quinpirole, the bivalent ligand **3b** induced further cAMP accumulation, indicating that its D_2_R-mediated effect was missing in the coexpressing cell line. Data represent mean±s.e.m. of 3–11 (for details see Supplementary Table 4) independent experiments each performed in triplicate; v=vehicle (PBS+10 μM forskolin).

To measure activation profiles of the bivalent ligands **2b** and **3b** and their monovalent analogues **2g** and **3g** comprising the pharmacophore of a D_2_R antagonist and a D_2_R agonist, respectively, we performed a BRET-based cAMP accumulation assay^43^. Coupling to inhibitory Gα_i/o_ proteins, the stimulation of the D_2_R leads to a decrease of cAMP, whereas activation of the Gα_s_-coupled NTS_1_R increases adenylyl cyclase activity. As expected, the reference agonist quinpirole potently inhibited forskolin-induced cAMP accumulation in cells expressing D_2_R only, while the phenylpiperazine-derived ligands **2b** and **2g** and the NTS_1_R-binding fragment NT(8-13) remained without significant effects (Fig. 5a).

Since we were unable to detect intrinsic activity for type 2 ligands in our cAMP accumulation assay, we tested the representative bivalent and monovalent ligands **2b** and **2g** for their capacity to prevent quinpirole-mediated inhibition of cAMP accumulation. As expected, both ligands were able to fully inhibit the effect of 10 nM quinpirole. In comparison, the type 1 ligand **1b** and its pharmacologically active D_2_R fragment eticlopride were more potent and even showed an inverse agonist effect, leading to a 20–32% change in the basal cAMP level (Supplementary Fig. 8). The bivalent ligand **3b** and the monovalent dopaminergic **3g** bearing the aminoindane moiety displayed functional properties that were highly similar to quinpirole. Observed potencies (EC_50_) were in the low nanomolar range (2.3–5.0 nM), and maximum efficacies did not differ significantly among the three investigated D_2_R agonists (Fig. 5d and Supplementary Table 4). In cells expressing only NTS_1_R, neither the monovalent ligands **2g**, **3g**, nor quinpirole were able to exhibit receptor activation. However, the bivalent ligands **2b** and **3b** were as effective as NT(8-13), albeit at 10-fold higher concentrations (EC_50_ 2.6 nM for NT(8-13) versus EC_50_ 20.7 and 30.6 nM for **2b** and **3b,** Fig. 5b,e). In D_2_R/NTS_1_R-coexpressing cells, quinpirole and the monovalent D_2_R agonist **3g** inhibited cAMP formation with similar potencies compared with cells monoexpressing D_2_R. The monovalent phenylpiperazine **2g** had no effect on the intracellular cAMP concentration. Interestingly, all investigated bivalent ligands increased cytosolic cAMP in a similar manner as NT(8-13). Observed potencies were comparable to the monoexpressing NTS_1_R cells (EC_50_ 2.0 nM for NT(8-13) versus 39.3 and 70.0 nM for **2b** and **3b**, respectively), although a slight loss in potency could be observed for the bivalent ligands (Fig. 5c,f). The extremely high affinity of the bivalent ligands could not be transduced into an increase in potency at D_2_R/NTS_1_R-coexpressing cells. When the spacer connecting the D_2_R and NTS_1_R pharmacophores was elongated to 66 atoms for the bivalent ligands **2c** and **3c**, similar observations concerning the activation of D_2_R, NTS_1_R and D_2_R/NTS_1_R heterodimers were made (Supplementary Fig. 9a–c and Supplementary Table 4).

The absence of D_2_R-mediated inhibition of cAMP accumulation in cells coexpressing D_2_R/NTS_1_R, is not specific for bivalent ligands, since a comparable attenuation of dopaminergic signalling is achieved when D_2_R/NTS_1_R-coexpressing cells are stimulated with quinpirole and NT(8-13) simultaneously (Supplementary Fig. 10a). In contrast, a reciprocal inhibition of NTS_1_R signalling by increasing concentrations of D_2_R agonist could not be observed (Supplementary Fig. 10b).

To exclude interference from forskolin stimulation or the relative receptor stoichiometry, experiments were also performed in the absence of forskolin and under conditions leading to enhanced NTS_1_R expression and therefore higher propensity to obtain D_2_R/NTS_1_R heterodimers. As illustrated in Supplementary Fig. 11a–e, these modifications did not result in significant changes of the receptor activation profiles of quinpirole, NT(8-13) or the bivalent ligands **2b** and **3b**. Coexpression of NTS_1_R and a signalling incompetent D_2_R-mutant (D80A)^44^,^45^ led to a loss of dopamine receptor signalling for the monovalent dopaminergic **3g** and quinpirole while preserving the above described biphasic-binding behaviour and the activation profile of bivalent ligand **3b** (Supplementary Fig. 12a,b) in D_2_R_D80A/NTS_1_R-coexpressing cells.

**Figure 6:**
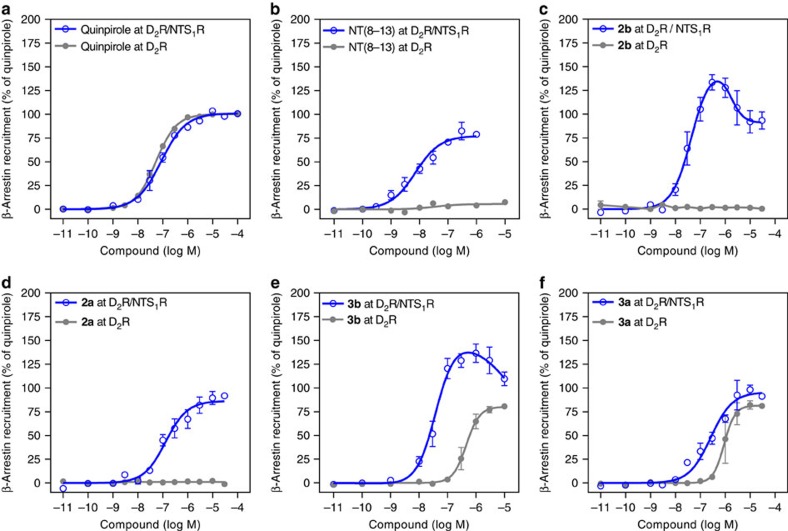
β-Arrestin-2 recruitment at D_2_R and D_2_R/NTS_1_R heterodimers. β-Arrestin-2 recruitment was determined employing an assay based on enzyme complementation. HEK 293 cells stably expressing β-arrestin-2 tagged with the EA were transfected with ProLink-tagged D_2_R with (open blue circles) or without (filled grey circles) cotransfection of NTS_1_R (**a**) Quinpirole induces β-arrestin-2 recruitment in cells singly expressing D_2_R (*n*=9) and cells coexpressing D_2_R/NTS_1_R (*n*=11) with similar potencies. (**b**) NT(8-13) induces β-arrestin-2 recruitment with a maximum effect of 77±3% in cells coexpressing D_2_R/NTS_1_R (*n*=5), but not in D_2_R monoexpressing cells (*n*=4). (**c**) The phenylpiperazine-derived bivalent ligand **2b** has no intrinsic activity in cells expressing D_2_R only (*n*=3), while it potently induces β-arrestin-2 recruitment in cells coexpressing D_2_R/NTS_1_R (*n*=7). Instead of a sigmoid curve, a bell-shaped dose–response profile is observed with a superior maximum effect as compared with both reference agonists. (**d**) The bivalent ligand **2a** does not lead to β-arrestin-2 recruitment in D_2_R monoexpressing cells (*n*=3), but causes a typical sigmoid dose–response curve in the D_2_R/NTS_1_R-coexpressing cells (*n*=7). (**e**) The aminoindane-based bivalent ligand **3b** induces β-arrestin-2 recruitment in cells expressing D_2_R (*n*=4) and cells coexpressing D_2_R/NTS_1_R (*n*=6). Coexpression of NTS_1_R leads to a significant increase in potency and efficacy and a bell-shaped dose–response curve as observed in **c**. (**f**) The aminoindane-type agonist with a 22-atom spacer (**3a**) leads to β-arrestin-2 recruitment in both types of transfected cells (*n*=3 for D_2_R and *n*=5 for D_2_R/NTS_1_R). Data represent mean±s.e.m. of *n* independent experiments, each performed in duplicate. Results were normalized to the maximum effect of quinpirole (100% for D_2_R and D_2_R/NTS_1_R).

Besides G proteins, a class of adaptor proteins called β-arrestins are frequently found to interact with GPCRs. The recruitment of β-arrestin to a GPCR can lead to internalization but also initiate signalling events distinct from the G protein-mediated response^46^. To investigate the interaction of D_2_R/NTS_1_R heterodimers with β-arrestin-2, we made use of an assay system based on enzyme complementation (DiscoveRx PathHunter). Hence, HEK 293 cells stably expressing β-arrestin-2 fused to an enzyme acceptor (EA, galactosidase fragment) were transiently transfected with ProLink-tagged D_2_R together with or without cotransfection of wild-type NTS_1_R. Upon recruitment of β-arrestin-2 to D_2_R, the following enzyme complementation leads to conversion of a substrate and thereby chemiluminescence. The D_2_R agonist quinpirole induced β-arrestin-2 recruitment in D_2_R monoexpressing and D_2_R/NTS_1_R-coexpressing cells with similar potencies (EC_50_ 55±3 versus 75±11 nM). Interestingly, stimulation with NT(8-13) induced β-arrestin-2 recruitment in the coexpressing cells, but not cells singly expressing D_2_R, indicating that β-arrestin-2 recruitment by NTS_1_R can be detected if it occurs in close proximity of D_2_Rs, as for example within a D_2_R/NTS_1_R heterodimer. Although the maximum effect remained below the response of quinpirole (77±3%), NT(8-13) elicited that response at 10-fold lower concentrations (EC_50_ 7.5±2.1 nM; Fig. 6a,b). Application of an equimolar combination of both agonists led to an even enhanced efficiency of β-arrestin-2 recruitment (*E*_max_ 136±6%; Supplementary Fig. 13a).

For the bivalent ligands of the phenylpiperazine-type **2a** and **2b** with 22- and 44-atom spacers, no β-arrestin-2 recruitment was observed in cells expressing D_2_R only, which is in good agreement with the antagonist properties observed for **2a** and **2b** in the cAMP accumulation assay. Interestingly, a bell-shaped dose–response curve was observed for the bivalent ligand **2b** in D_2_R/NTS_1_R-coexpressing cells. Maximum β-arrestin-2 recruitment was determined at a concentration of 300 nM (*E*_max_ 133%), while higher ligand concentrations led to an attenuated response (Fig. 6c). In contrast, a typical sigmoid dose–response curve was observed for the analogue **2a** with the shorter 22-atom spacer (EC_50_ 110±20 nM, *E*_max_ 88±4%, Fig. 6d). As expected, the bivalent ligands **3a** and **3b** bearing the aminoindane-type D_2_R agonist substructure elicited β-arrestin-2 recruitment in cells monoexpressing D_2_R (EC_50_ 1,500±500 nM, *E*_max_ 87±5% and EC_50_ 580±130 nM, *E*_max_ 82±1% for **3a** and **3b** respectively, Fig. 6e,f). Highly similar to the activation profiles of type 2 compounds, a bell-shaped dose–response curve with a maximum effect at a concentration of 300 nM to 1 μM (*E*_max_ 136%) was observed when NTS_1_R was coexpressed for **3b** (44-atom spacer), but not **3a** (22-atom spacer, EC_50_ 37±8 nM, *E*_max_ 105±9%, Fig. 6e,f). Bell-shaped dose–response curves with enhanced efficacy were also observed for the bivalent ligands **2c** and **3c** (66-atom spacer; Supplementary Fig. 13b,c).

Additional experiments were performed employing a ProLink-tagged signalling incompetent D_2_R_D80A mutant coexpressed with NTS_1_R. Under these conditions, only NTS_1_R-mediated β-arrestin-2 recruitment can be detected. As expected, the NTS_1_R agonist NT(8-13), but not the D_2_R agonist quinpirole, was able to induce β-arrestin-2 recruitment in D_2_R_D80A/NTS_1_R-coexpressing cells. The potency of NT(8-13) was highly comparable to cells coexpressing wild-type D_2_R/NTS_1_R (EC_50_ 5.9±1.2 nM for D_2_R_D80A/NTS_1_R and EC_50_ 7.5±2.1 nM for wild-type D_2_R/NTS_1_R; Supplementary Fig. 14a,b). Highly similar to the results obtained with intact D_2_R, bivalent ligands with a short spacer (22-atoms, **2a**, **3a**) resulted in sigmoid dose–response curves (EC_50_ 67±23 nM, *E*_max_ 98±4% and EC_50_ 190±40 nM, *E*_max_ 113±7%, for **2a** and **3a**) in cells coexpressing NTS_1_R with the signalling incompetent D_2_R mutant, while bell-shaped curves with increased maximum efficacy were observed for the ligands with the longer spacer (**2b**,**c**, **3b**,**c**, 44- and 66-atom spacer, Supplementary Fig. 14c–h). Ligands **2b**,**c** and **3b**,**c** reached maximum effects up to 210% relative to NT(8-13).

When the same experiments were performed in HEK 293 cells coexpressing NTS_1_R with ProLink-tagged protease-activated receptor subtype 2 (PAR_2_), only a very weak recruitment of β-arrestin-2 was observed for NT(8-13) and the representative bivalent ligands **2c** and **3b** (≤ 19%) compared with the PAR_2_ agonist f-LIGRLO-NH_2_ (ref. 47). The D_2_R agonist quinpirole was entirely inactive. Importantly, all dose–response curves showed a typical sigmoid profile (Supplementary Fig. 15a–d). These results indicate a specific effect of the bivalent ligands leading to bell-shaped dose–response curves in D_2_R/NTS_1_R-coexpressing cells.

## Discussion

GPCR exist as monomers or cross-react forming dimers and higher-order oligomers. Because dimerization of GPCRs can result in modified ligand-binding and -signalling properties, a selective targeting of these entities is a powerful strategy in chemical biology and drug discovery. Irrespective of whether or not dimerization has physiological consequences *per se*, medicinal chemistry can take advantage of this phenomenon targeting drugs towards cells coexpressing an individual dimer-forming combination of GPCRs. In theory, bivalent ligands successfully bridging two binding sites of adjacent protomers should confer extremely high affinity (resulting from the total binding energy of two recognition elements) and thus selectivity for the receptor heterodimer. Most of the previous reports have shown compounds with only modest preference for heterodimers over monomers. In many cases, it has not been demonstrated that the two linked pharmacophores address two orthosteric-binding sites of two neighbouring protomers.

GPCR crystal structures may leverage an effective development of novel molecular probes and drug candidates^48^, because they can be used for structure-based *in silico* docking screens, giving access to new chemotypes and, as a consequence, to new biological profiles. Furthermore, they can guide the evolution of novel ligands by providing insights into attractive and repulsive ligand–receptor interactions and the relative topology of crucial moieties. Both strategies can be performed based on either the crystal structure of a given GPCR or starting from a homology model of a structurally highly similar congener. Using the co-crystallized ligands eticlopride and NT(8-13) as fragments for the design of bivalent ligands, the recently resolved X-ray crystal structures of NTS_1_R, D_3_R and a β_1_-AR dimer combined with homology modelling enabled us to determine the relative disposition of the pharmacophores to each other and to identify suitable attachment points for the spacer units. The strategy allowed a rational, structure-guided development of bivalent D_2_R/NTS_1_R ligands. The compounds **1b**-**d**, **2b**-**d** and **3b**-**d** show unprecedented selectivity (up to three orders of magnitude) and binding affinity in the picomolar range for cells coexpressing both GPCRs, compared with cells that only express D_2_Rs. Preparations of porcine striatal membranes were used to investigate the biological relevance of our bivalent ligands. Although differences between high- and low-affinity binding sites were smaller, biphasic-binding curves confirmed a bivalent-binding behaviour.

A functional switch was observed for bivalent ligands containing a dopamine receptor agonist moiety. The compounds **3b** and **3c** behaved as agonists in cells singly expressing D_2_R inhibiting cAMP formation. However, no inhibitory effect on the NTS_1_R-promoted cAMP accumulation resulting from NTS_1_R activation by the NT(8-13) fragment was observed in D_2_R/NTS_1_R-coexpressing cells. Thus, the bivalent ligands **3b** and **3c** containing a D_2_R agonist pharmacophore behaved identical to bivalent ligands bearing a D_2_R antagonist moiety (**2b** and **2c**), pointing towards a NTS_1_R-dominated signalling behaviour within D_2_R/NTS_1_R heterodimers. Yet, the exact molecular mechanism underlying this phenomenon is not fully understood. In particular, the extremely high binding affinity could not be translated into activation potency. The observed cAMP accumulation may be caused by monovalent binding to NTS_1_R, if the bivalently bound D_2_R/NTS_1_R heterodimer is unable to activate G proteins. However, interpretation of the functional experiments is far from trivial, as the overall response results from the activation of mixed populations of D_2_R and NTS_1_R monomers, homomers and heteromers. Moreover when dealing with bivalent ligands, at least three different (and probably even more) binding modes have to be considered: a monovalent-binding mode to each protomer as well as a bivalent, receptor-bridging binding mode.

As a second signalling pathway, we investigated the recruitment of β-arrestin-2. Employing an assay based on enzyme complementation, we could determine D_2_R-mediated β-arrestin-2 recruitment; while NTS_1_R-mediated engagement of β-arrestin-2 was only detectable in presence of the ProLink-tagged D_2_R. Compared with cells singly expressing D_2_Rs, coexpression and activation of NTS_1_R leads to a significant increase in potency. Thus, NTS_1_R protomer appears to dominate not only G protein coupling but also β-arrestin-2 recruitment in D_2_R/NTS_1_R-coexpressing cells. Importantly, bell-shaped dose–response curves were observed for the bivalent ligands **2b**,**c** and **3b**,**c**, whereas the structural analogues **2a** and **3a** with a shorter linker or a combination of two monovalent orthosteric ligands (quinpirole and NT(8-13)) showed regular sigmoid dose–response curves. In analogy to the binding behaviour of **2b**,**c** and **3b**,**c** (biphasic curves), the atypical dose–response relationship suggests a concentration-dependent contribution of different modes of receptor–ligand interactions. While it is not clear how exactly different binding modes influence the receptors' capacity to recruit β-arrestin-2, the atypical dose–response curves obviously indicate that bivalent ligands with adequately designed spacer units display receptor activation characteristics distinct from monovalent ligands. Although the simultaneous presence of bivalent, receptor-bridging binding modes and monovalent-binding modes for bivalent ligands may represent a valid concept, we cannot exclude other, probably allosteric effects, leading to an altered binding and signalling behaviour.

Even though the exact molecular mechanism underlying the atypical functional behaviour remains to be elucidated, our study demonstrates the successful development of bi-orthosteric bivalent ligands targeting D_2_R/NTS_1_R heterodimers with unique properties. Because our target receptors are of major relevance for the pathophysiology of neurological and psychiatric disorders including Parkinson's disease and schizophrenia, the D_2_R/NTS_1_R heterodimer may be a promising pharmacological target^17^. The tissue selectivity of bivalent D_2_R/NTS_1_R ligands may confer high potency and reduced side effects. Presumably, the *in vivo* bioavailability of our compounds of types 1–3 will not be suitable for their use as a drug. However, our newly developed bivalent ligands represent powerful pharmacological tools and may serve as a starting point for the development of innovative imaging agents and drugs addressing GPCR heterodimers, as sophisticated drug-delivery systems are currently developed.

## Methods

### Molecular modelling

D_2_R/NTS_1_R dimer models were generated by superimposing both our recently described homology model of the D_2_R (ref. 40) (which was based on the D_3_R crystal structure^36^) and the NTS_1_R crystal structure (PDB-ID 4BUO)^39^ with the so far resolved crystal structures of GPCR dimers. For details on the selection process see Supplementary Note 1 and Supplementary Tables 1 and 2. The final dimer model, created based on the crystal structure of the β_1_-AR dimer (PDB-ID 4GPO)^41^, was submitted to an energy minimization procedure as described previously^40^. Ligand positions were obtained in different ways. The coordinates of NT(8-13) in the crystal structure of NTS_1_R (ref. 39) were maintained for its position in the heterodimer model. The position of eticlopride was obtained by superimposing the crystal structure of D_3_R (ref. 36), including co-crystallized eticlopride, with our D_2_R homology model, followed by a transfer of the eticlopride coordinates to the D_2_R homology model. Coordinates of the remaining compounds were achieved by docking using AutoDock Vina^49^ as described previously^40^. Out of the 20 best-ranked conformations, one final conformation for each ligand was selected based on the scoring function of AutoDock Vina, experimental data and a manual inspection followed by an additional energy minimization. The all-atom force field ff99SB (ref. 50) was used for receptors and NT(8-13) and the general AMBER force field (GAFF)^51^ was used for the remaining ligands. A formal charge of +2 was assigned to NT(8-13), with the N-terminus and side chains of arginine protonated and the C-terminus deprotonated. A formal charge of +1 was assigned to the D_2_R ligands, here the basic nitrogen was protonated. Further details on heterodimer model generation, docking procedures and MD simulations are provided in Supplementary Notes 1–3; Supplementary Figs 1–4; and Supplementary Tables 1 and 2. Snapshots of MD simulations are provided as Supplementary Data 1, 2, 3. All figures were prepared using the UCSF Chimera package 1.10 (ref. 52).

### Synthesis and characterization of bivalent ligands

Detailed schemes and conditions for the synthesis of the bivalent ligands **1a**–**d, 2a**–**f** and **3a**–**f** and the monovalent analogues **2g** and **3g** are provided in Supplementary Figs 16–18. Detailed methods and characterization for all compounds and precursors are provided as Supplementary Methods. For nuclear magnetic resonance analysis of the small molecules described in this article, see Supplementary Figs 19–34.

### Cell culture

HEK 293 T cells (ATCC accession number CRL-11268) and HEK 293 cells stably expressing the EA-tagged β-arrestin-2 fusion protein (DiscoveRx) were maintained in DMEM/F-12 supplemented with 10% fetal bovine serum, 2 mM L-glutamine, 100 μg ml^−1^ penicillin, 100 μg ml^−1^ streptomycin and 150 μg ml^−1^ hygromycin for EA-β-arrestin-2 cells at 37 °C, 5% CO_2_ (all cell culture reagents purchased from Invitrogen/Thermo Fisher Scientific). Cell lines were tested for mycoplasma contamination using the MycoAlert Plus detection kit (Lonza, Verviers, Belgium) on a regular basis.

### Receptor-binding experiments

Receptor-binding studies were carried out in analogy to a previously described method^53^. Accordingly, competition-binding experiments with the human D_2L_R were perfomed using preparations of membranes from HEK 293 T cells, which were transiently transfected with the D_2L_R (from Missouri S&T cDNA Resource Center (UMR), Rolla, MO) using the Mirus TransIT-293 transfection reagent (purchased from MoBiTec, Goettingen, Germany). The assays were carried out in binding buffer (50 mM Tris pH 7.4, 5 mM MgCl_2_, 1 mM EDTA, 100 μg ml^−1^ bacitracin and 100 μg ml^−1^ soybean trypsin inhibitor) at a final volume of 200 μl with a protein concentration of 5–8 μg per assay tube, *K*_D_ values of 0.048–0.060 nM and corresponding *B*_max_ values of 960–970 fmol mg^−1^, together with [^3^H]spiperone (specific activity 81 Ci mmol^−1^, PerkinElmer, Rodgau, Germany) at a final concentration of 0.20–0.25 nM. Binding experiments with the co-transfected receptors were performed using membrane preparations from HEK 293 T cells, which were transiently transfected (Mirus TransIT-293) with the D_2L_R and NTS_1_R (from UMR) in the appropriate ratio of cDNA. Competition-binding experiments with the resulting homogenates of membranes coexpressing both receptors were carried out at a protein concentration of 1–8 μg per assay tube together with [^3^H]spiperone at a final concentration of 0.10–0.25 nM. For the detailed investigation of the heterobivalent ligands membranes with receptor densities of D_2L_R and NTS_1_R in the ratio of 1:2 (*K*_D_ values of 0.053–0.080 nM, *B*_max_=800 fmol mg^−1^ for D_2L_R, 1,500–2,000 fmol mg^−1^ for NTS_1_R), 2:1 (*K*_D_ 0.040 nM, *B*_max_=2,000 fmol mg^−1^ D_2L_R 1,000 fmol mg^−1^ NTS_1_R) and 3:1 (*K*_D_ 0.050 nM, *B*_max_=8,000 fmol mg^−1^ D_2L_R, 2,500 fmol mg^−1^ NTS_1_R) were used. Competition-binding experiments with [^3^H]neurotensin (final concentratrion 0.50 nM, specific activity 101 Ci mmol^−1^; PerkinElmer, Rodgau, Germany) were performed at a protein concentration of 2 μg per assay tube and relative expression levels of 2.5:1 (*B*_max_=3,000 fmol mg^−1^ D_2L_R, 1,200 fmol mg^−1^ NTS_1_R, *K*_D_ 0.50 nM). Unspecific binding was determined in the presence of haloperidol (10 μM for D_2L_R) or NT(8-13) (10 μM for NTS_1_R). Protein concentration was established by the method of Lowry using bovine serum albumin as standard^54^.

Binding experiments with porcine striatal membranes were performed as described above together with [^3^H]spiperone (final concentration 0.20–0.24 nM) at a protein concentration of 20 μg per tube (B_max_=220 fmol mg^−1^ D_2L_R, 140 fmol mg^−1^ NTS_1_R, *K*_D_=0.090 nM).

### Data analysis

The resulting competition curves of the receptor-binding experiments were analysed by nonlinear regression using the algorithms in PRISM 6.0 (GraphPad Software, San Diego, CA). For each individual experiment, the data were fitted using a monophasic competition model to provide an IC_50_ value, which was then transformed into a *K*_i_ value according to the equation of Cheng and Prusoff^55^. The monophasic fit was accepted, unless a biphasic competition model providing two individual values for *K*_i high_ and *K*_i low_ resulted in a statistically significant better fitting of the data (extra sum-of-squares F-test, P<0.05).

### cAMP BRET Assay

HEK 293 T cells were transiently transfected with pcDNA3L-His-CAMYEL (ref. 43) (ATCC) and D_2S_R and/or NTS_1_R at a cDNA ratio of 2:2 or 2:2:2 (unless indicated otherwise), respectively, using Mirus TransIT-293 transfection reagent. Resulting receptor expression levels were determined in saturation-binding experiments with membranes from transfected HEK 293 T cells and found to be 21±6 pmol mg^−1^ protein for D_2S_R monoexpression, 25±13 pmol mg^−1^ for NTS_1_R monoexpression and 15±4 and 7.2±1.7 pmol mg^−1^ for D_2S_R and NTS_1_R (1:1 transfection ratio) or 5.6±3.0 and 26±11 pmol mg^−1^ for D_2S_R and NTS_1_R (0.5:3.5 transfection ratio), respectively in the coexpressing cells. 24 h post-transfection cells were seeded into white half-area 96-well plates at 2.0 × 10^4^ cells per well and grown overnight. On the following day phenol red free medium was removed and replaced by PBS and cells were serum starved for 1 h before treatment. The assay was started by adding 10 μl coelenterazine-h (Promega, Mannheim, Germany) to each well to yield a final concentration of 5 μM. After 5 min incubation, compounds were added in PBS containing 50 μM forskolin (final concentration 10 μM). Reads of the plates started 15 min after agonist addition. BRET readings were collected using a CLARIOstar plate reader (BMG LabTech, Ortenberg, Germany). Emission signals from Renilla Luciferase and YFP were measured simultaneously using a BRET^1^ filter set (475–30 nm/535–30 nm). BRET ratios (emission at 535–30 nm/emission at 475–30 nm) were calculated and dose–response curves were fitted by nonlinear regression using the algorithms of PRISM 6.0. Curves were normalized to basal BRET ratio obtained from dPBS (0%) and the effect of 10 μM forskolin (100%).

### β-Arrestin-2 recruitment assay

The measurement of β-arrestin-2 recruitment stimulated by receptor activation was performed by utilizing the PathHunter assay purchased from DiscoveRx (Birmingham, UK) according to the manufacturer's protocol. Accordingly, HEK 293 cells stably expressing the EA-tagged β-arrestin-2 fusion protein (provided by DiscoveRx) were transiently transfected with the ProLink(ARMS2-PK2)-tagged D_2S_R (or the respective ARMS2-PK2-tagged D_2S_R_D80A mutant) together with or without cotransfection of NTS_1_R at a cDNA ratio of 1:3 using Mirus TransIT-293 transfection reagent. Resulting receptor expression levels were determined in saturation-binding expriments with membranes from the same pool of cells and found to be 3.5±0.9 pmol mg^−1^ protein for D_2S_R and 4.2±0.3 pmol mg^−1^ for NTS_1_R in the D_2_R/NTS_1_R coexpression and 3.5±1.2 and 11.5±3.3 pmol mg^−1^ for D_2S_R_D80A and NTS_1_R, respectively for coexpression of the signalling incompetent D_2_R mutant together with NTS_1_R. 24 h after transfection, cells were detached using Versene (Invitrogen), 5,000 cells per well were seeded in 384-well plates and maintained at 37 °C, 5% CO_2_ for 24 h. After incubation with different concentrations of test compounds (from 10^−15^ to 10^−4^ M final concentration) in duplicates for 5 h, the detection mix was added and incubation was continued for further 60 min. Chemiluminescence was determined with a CLARIOstar reader for microplates (BMG LabTech). Resulting responses were normalized to the maximum effect obtained with quinpirole (100%) and the basal response (vehicle, 0%), or relative to the response of NT(8-13) (100%) when the signalling incompetent D_2S_R_D80A mutant was used. Dose–response curves were calculated by nonlinear regression using the algorithms of PRISM 6.0. Control experiments were performed using the same approach and transfection of ProLink(PK1)-tagged PAR_2_ together with wild-type NTS_1_R. Under these conditions, NTS_1_R expression levels were determined to be 8.7±1.2 pmol mg^−1^ protein. Resulting responses were then normalized to the effect of the PAR_2_ agonist f-LIGRLO-NH_2_.

### Data availability

The data that support the findings of this study are available within the Supplementary Information files and/or from the corresponding authors upon request.

## Additional information

**How to cite this article:** Hübner, H. *et al*. Structure-guided development of heterodimer-selective GPCR ligands. *Nat. Commun.* 7:12298 doi: 10.1038/ncomms12298 (2016).

## Supplementary Material

Supplementary Information Supplementary Figures 1-34, Supplementary Tables 1-4, Supplementary Notes 1-3, Supplementary Methods and Supplementary References

Supplementary Data 1 MD ligand 1b

Supplementary Data 2 MD ligand 2b

Supplementary Data 3 MD ligand 3b

## Figures and Tables

**Table 1:**
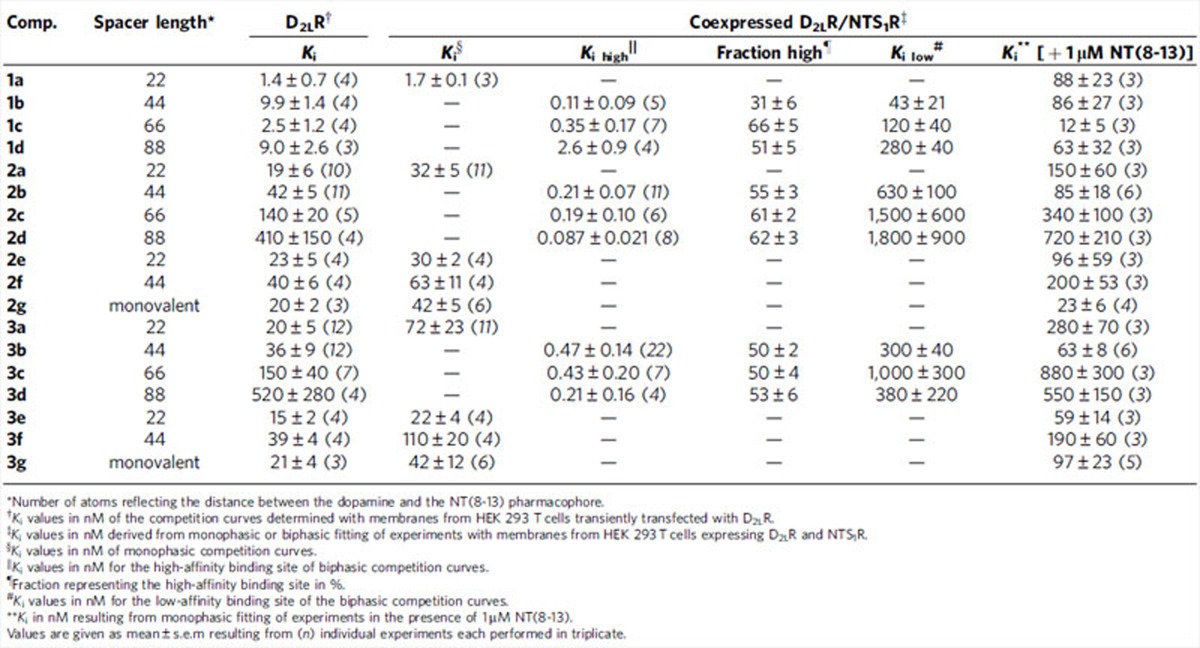
D_2_R-affinity for ligands 1a–d, 2a–g and 3a–g determined by [^3^H]spiperone displacement.

## References

[b1] Kobilka B. K. G protein coupled receptor structure and activation. Biochim. Biophys. Acta 1768, 794–807 (2007). 17188232 10.1016/j.bbamem.2006.10.021 PMC1876727

[b2] Bouvier M. Oligomerization of G-protein-coupled transmitter receptors. Nat. Rev. Neurosci. 2, 274–286 (2001). 11283750 10.1038/35067575

[b3] Ferre S., Ciruela F., Woods A. S., Lluis C. & Franco R. Functional relevance of neurotransmitter receptor heteromers in the central nervous system. Trends Neurosci. 30, 440–446 (2007). 17692396 10.1016/j.tins.2007.07.001

[b4] George S. R., O'Dowd B. F. & Lee S. P. G-protein-coupled receptor oligomerization and its potential for drug discovery. Nat. Rev. Drug Discov. 1, 808–820 (2002). 12360258 10.1038/nrd913

[b5] Han Y., Moreira I. S., Urizar E., Weinstein H. & Javitch J. A. Allosteric communication between protomers of dopamine class A GPCR dimers modulates activation. Nat. Chem. Biol. 5, 688–695 (2009). 19648932 10.1038/nchembio.199 PMC2817978

[b6] Bellot M. . Dual agonist occupancy of AT1-R-alpha2C-AR heterodimers results in atypical Gs-PKA signaling. Nat. Chem. Biol. 11, 271–279 (2015). 25706338 10.1038/nchembio.1766 PMC4465922

[b7] Milligan G. G protein-coupled receptor hetero-dimerization: contribution to pharmacology and function. Br. J. Pharmacol. 158, 5–14 (2009). 19309353 10.1111/j.1476-5381.2009.00169.x PMC2795239

[b8] Guo W. . Dopamine D2 receptors form higher order oligomers at physiological expression levels. EMBO J. 27, 2293–2304 (2008). 18668123 10.1038/emboj.2008.153 PMC2529367

[b9] Albizu L. . Time-resolved FRET between GPCR ligands reveals oligomers in native tissues. Nat. Chem. Biol. 6, 587–594 (2010). 20622858 10.1038/nchembio.396 PMC3506176

[b10] Scarselli M. . D2/D3 dopamine receptor heterodimers exhibit unique functional properties. J. Biol. Chem. 276, 30308–30314 (2001). 11373283 10.1074/jbc.M102297200

[b11] Lee S. Oligomerization of dopamine and serotonin receptors. Neuropsychopharmacology. 23, S32–S40 (2000). 11008065 10.1016/S0893-133X(00)00155-X

[b12] Hillion J. . Coaggregation, cointernalization, and codesensitization of adenosine A2A receptors and dopamine D2 receptors. J. Biol. Chem. 277, 18091–18097 (2002). 11872740 10.1074/jbc.M107731200

[b13] Urizar E. . CODA-RET reveals functional selectivity as a result of GPCR heteromerization. Nat. Chem. Biol. 7, 624–630 (2011). 21785426 10.1038/nchembio.623 PMC3158273

[b14] Hasbi A. . Calcium signaling cascade links dopamine D1–D2 receptor heteromer to striatal BDNF production and neuronal growth. Proc. Natl Acad. Sci. USA 106, 21377–21382 (2009). 19948956 10.1073/pnas.0903676106 PMC2795506

[b15] Wang M. . Schizophrenia, amphetamine-induced sensitized state and acute amphetamine exposure all show a common alteration: increased dopamine D2 receptor dimerization. Mol. Brain 3, 25 (2010). 20813060 10.1186/1756-6606-3-25 PMC2942879

[b16] Bagalkot T. R. . Chronic social defeat stress increases dopamine D2 receptor dimerization in the prefrontal cortex of adult mice. Neuroscience 311, 444–452 (2015). 26484605 10.1016/j.neuroscience.2015.10.024

[b17] Binder E. B., Kinkead B., Owens M. J. & Nemeroff C. B. Neurotensin and dopamine interactions. Pharmacol. Rev. 53, 453–486 (2001). 11734615

[b18] Boules M., Fredrickson P., Muehlmann A. & Richelson E. Elucidating the role of neurotensin in the pathophysiology and management of major mental disorders. Behav. Sci. 4, 125–153 (2014). 25379273 10.3390/bs4020125 PMC4219245

[b19] Fuxe K. . Intramembrane interactions between neurotensin receptors and dopamine D2 receptors as a major mechanism for the neuroleptic-like action of neurotensin. Ann. NY Acad. Sci. 668, 186–204 (1992). 1361113 10.1111/j.1749-6632.1992.tb27350.x

[b20] Koschatzky S., Tschammer N. & Gmeiner P. Cross-receptor interactions between dopamine D2L and neurotensin NTS1 receptors modulate binding affinities of dopaminergics. ACS Chem. Neurosci. 2, 308–316 (2011). 22778874 10.1021/cn200020y PMC3369761

[b21] Borroto-Escuela D. O. . Dopamine D2 receptor signaling dynamics of dopamine D2-neurotensin 1 receptor heteromers. Biochem. Biophys. Res. Commun. 435, 140–146 (2013). 23624386 10.1016/j.bbrc.2013.04.058

[b22] Kinkead B., Binder E. B. & Nemeroff C. B. Does neurotensin mediate the effects of antipsychotic drugs? Biol. Psychiatry 46, 340–351 (1999). 10435199 10.1016/s0006-3223(99)00070-0

[b23] Hiller C., Kühhorn J., Gmeiner P. & Class A. G-protein-coupled receptor (GPCR) dimers and bivalent ligands. J. Med. Chem. 56, 6542–6559 (2013). 23678887 10.1021/jm4004335

[b24] Shonberg J., Scammells P. J. & Capuano B. Design strategies for bivalent ligands targeting GPCRs. ChemMedChem. 6, 963–974 (2011). 21520422 10.1002/cmdc.201100101

[b25] Waldhoer M. . A heterodimer-selective agonist shows *in vivo* relevance of G protein-coupled receptor dimers. Proc. Natl Acad. Sci. USA 102, 9050–9055 (2005). 15932946 10.1073/pnas.0501112102 PMC1157030

[b26] Daniels D. J. . Opioid-induced tolerance and dependence in mice is modulated by the distance between pharmacophores in a bivalent ligand series. Proc. Natl Acad. Sci. USA 102, 19208–19213 (2005). 16365317 10.1073/pnas.0506627102 PMC1323165

[b27] Akgün E. . Inhibition of inflammatory and neuropathic pain by targeting a Mu opioid receptor/chemokine Receptor5 heteromer (MOR-CCR5). J. Med. Chem. 58, 8647–8657 (2015). 26451468 10.1021/acs.jmedchem.5b01245 PMC5055304

[b28] McRobb F. M., Crosby I. T., Yuriev E., Lane J. R. & Capuano B. Homobivalent ligands of the atypical antipsychotic clozapine: design, synthesis, and pharmacological evaluation. J. Med. Chem. 55, 1622–1634 (2012). 22243698 10.1021/jm201420s

[b29] Butini S. . Discovery of bishomo(hetero)arylpiperazines as novel multifunctional ligands targeting dopamine D3 and serotonin 5-HT1A and 5-HT2A receptors. J. Med. Chem. 53, 4803–4807 (2010). 20481570 10.1021/jm100294b

[b30] Gogoi S. . Novel bivalent ligands for D2/D3 dopamine receptors: significant cooperative gain in D2 affinity and potency. ACS Med. Chem. Lett. 3, 991–996 (2012). 23275802 10.1021/ml3002117 PMC3530844

[b31] Soriano A. . Adenosine A2A receptor-antagonist/dopamine D2 receptor-agonist bivalent ligands as pharmacological tools to detect A2A-D2 receptor heteromers. J. Med. Chem. 52, 5590–5602 (2009). 19711895 10.1021/jm900298c

[b32] Kühhorn J. . Development of a bivalent dopamine D(2) receptor agonist. J. Med. Chem. 54, 7911–7919 (2011). 21999579 10.1021/jm2009919

[b33] Jacobson K. A., Xie R., Young L., Chang L. & Liang B. T. A novel pharmacological approach to treating cardiac ischemia: binary conjugates of A1 and A3 adenosine receptor agonists. J. Biol. Chem. 275, 30272–30279 (2000). 10887176 10.1074/jbc.M001520200 PMC3561767

[b34] Tschammer N. . Highly potent 5-aminotetrahydropyrazolopyridines: enantioselective dopamine D-3 receptor binding, functional selectivity, and analysis of receptor-ligand interactions. J. Med. Chem. 54, 2477–2491 (2011). 21388142 10.1021/jm101639t

[b35] Dörfler M., Tschammer N., Hamperl K., Hübner H. & Gmeiner P. Novel D3 selective dopaminergics incorporating enyne units as nonaromatic catechol bioisosteres: synthesis, bioactivity, and mutagenesis studies. J. Med. Chem. 51, 6829–6838 (2008). 18834111 10.1021/jm800895v

[b36] Chien E. Y. . Structure of the human dopamine D3 receptor in complex with a D2/D3 selective antagonist. Science 330, 1091–1095 (2010). 21097933 10.1126/science.1197410 PMC3058422

[b37] Löber S., Hübner H., Tschammer N. & Gmeiner P. Recent advances in the search for D3- and D4-selective drugs: probes, models and candidates. Trends Pharmacol. Sci. 32, 148–157 (2011). 21232805 10.1016/j.tips.2010.12.003

[b38] White J. F. . Structure of the agonist-bound neurotensin receptor. Nature 490, 508–513 (2012). 23051748 10.1038/nature11558 PMC3482300

[b39] Egloff P. . Structure of signaling-competent neurotensin receptor 1 obtained by directed evolution in *Escherichia coli*. Proc. Natl Acad. Sci. USA 111, E655–E662 (2014). 24453215 10.1073/pnas.1317903111 PMC3926081

[b40] Hiller C. . Functionally selective dopamine D2/D3 receptor agonists comprising an enyne moiety. J. Med. Chem. 56, 5130–5141 (2013). 23730937 10.1021/jm400520c

[b41] Huang J., Chen S., Zhang J. J. & Huang X.-Y. Crystal structure of oligomeric β1-adrenergic G protein–coupled receptors in ligand-free basal state. Nat. Struct. Mol. Biol. 20, 419–425 (2013). 23435379 10.1038/nsmb.2504 PMC3618578

[b42] Einsiedel J. . Discovery of highly potent and neurotensin receptor 2 selective neurotensin mimetics. J. Med. Chem. 54, 2915–2923 (2011). 21446649 10.1021/jm200006c

[b43] Jiang L. I. . Use of a cAMP BRET sensor to characterize a novel regulation of cAMP by the sphingosine 1-phosphate/G13 pathway. J. Biol. Chem. 282, 10576–10584 (2007). 17283075 10.1074/jbc.M609695200 PMC2526465

[b44] Peterson S. M. . Elucidation of G-protein and β-arrestin functional selectivity at the dopamine D2 receptor. Proc. Natl Acad. Sci. USA 112, 7097–7102 (2015). 25964346 10.1073/pnas.1502742112 PMC4460444

[b45] Neve K. A., Cox B. A., Henningsen R. A., Spanoyannis A. & Neve R. L. Pivotal role for aspartate-80 in the regulation of dopamine D2 receptor affinity for drugs and inhibition of adenylyl cyclase. Mol. Pharmacol. 39, 733–739 (1991). 1828858

[b46] Lefkowitz R. J. A brief history of G protein-coupled receptors (Nobel Lecture). Angew Chem. Int. Ed. Engl. 52, 6366–6378 (2013). 23650015 10.1002/anie.201301924

[b47] Hollenberg M. D. . Derivatized 2-furoyl-LIGRLO-amide, a versatile and selective probe for proteinase-activated receptor 2: binding and visualization. J. Pharmacol. Exp. Ther. 326, 453–462 (2008). 18477767 10.1124/jpet.108.136432

[b48] Shoichet B. K. & Kobilka B. K. Structure-based drug screening for G-protein-coupled receptors. Trends Pharmacol. Sci. 33, 268–272 (2012). 22503476 10.1016/j.tips.2012.03.007 PMC3523194

[b49] Trott O. & Olson A. J. AutoDock Vina: improving the speed and accuracy of docking with a new scoring function, efficient optimization, and multithreading. J. Comput. Chem. 31, 455–461 (2010). 19499576 10.1002/jcc.21334 PMC3041641

[b50] Hornak V. . Comparison of multiple Amber force fields and development of improved protein backbone parameters. Proteins 65, 712–725 (2006). 16981200 10.1002/prot.21123 PMC4805110

[b51] Jojart B. & Martinek T. A. Performance of the general amber force field in modeling aqueous POPC membrane bilayers. J. Comput. Chem. 28, 2051–2058 (2007). 17431937 10.1002/jcc.20748

[b52] Pettersen E. F. . UCSF Chimera—a visualization system for exploratory research and analysis. J. Comput. Chem. 25, 1605–1612 (2004). 15264254 10.1002/jcc.20084

[b53] Hübner H., Haubmann C., Utz W. & Gmeiner P. Conjugated enynes as nonaromatic catechol bioisosteres: synthesis, binding experiments and computational studies of novel dopamine receptor agonists recognizing preferentially the D3 subtype. J. Med. Chem. 43, 756–762 (2000). 10691700 10.1021/jm991098z

[b54] Lowry O. H., Rosebrough N. J., Farr A. L. & Randall R. J. Protein measurement with the folin phenol reagent. J. Biol. Chem. 193, 265–275 (1951). 14907713

[b55] Cheng Y.-C. & Prusoff W. H. Relationship between the inhibition constant (KI) and the concentration of inhibitor which causes 50 per cent inhibition (I50) of an enzymatic reaction. Biochem. Pharmacol. 22, 3099–3108 (1973). 4202581 10.1016/0006-2952(73)90196-2

