# Supplementary material for: Structure-guided development of heterodimer-selective GPCR ligands
Source: Nat Commun. 2016 Jul 26;7:12298. doi: 10.1038/ncomms12298 (PMC4963535; doi:10.1038/ncomms12298)
Supplement: Supplementary Information — Supplementary Figures 1-34, Supplementary Tables 1-4, Supplementary Notes 1-3, Supplementary Methods and Supplementary References [file ncomms12298-s1.pdf]

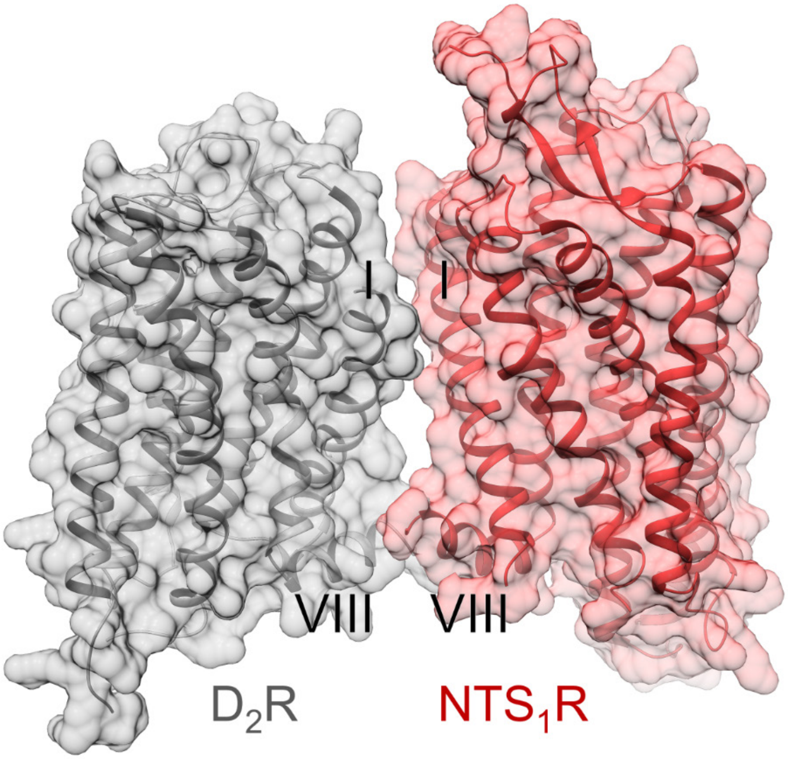


**Supplementary Figure 1. $D_2R/NTS_1R$ heterodimer model.** Transmembrane helices and surface of $D_2R$ and $NTS_1R$ are shown in grey and red, respectively. The dimer orientation is based on the $\beta_1$-AR crystal structure (4GPO) with the main contacts between receptor protomers via TM1 and helix 8. The dimer model was built as described in the methods section.

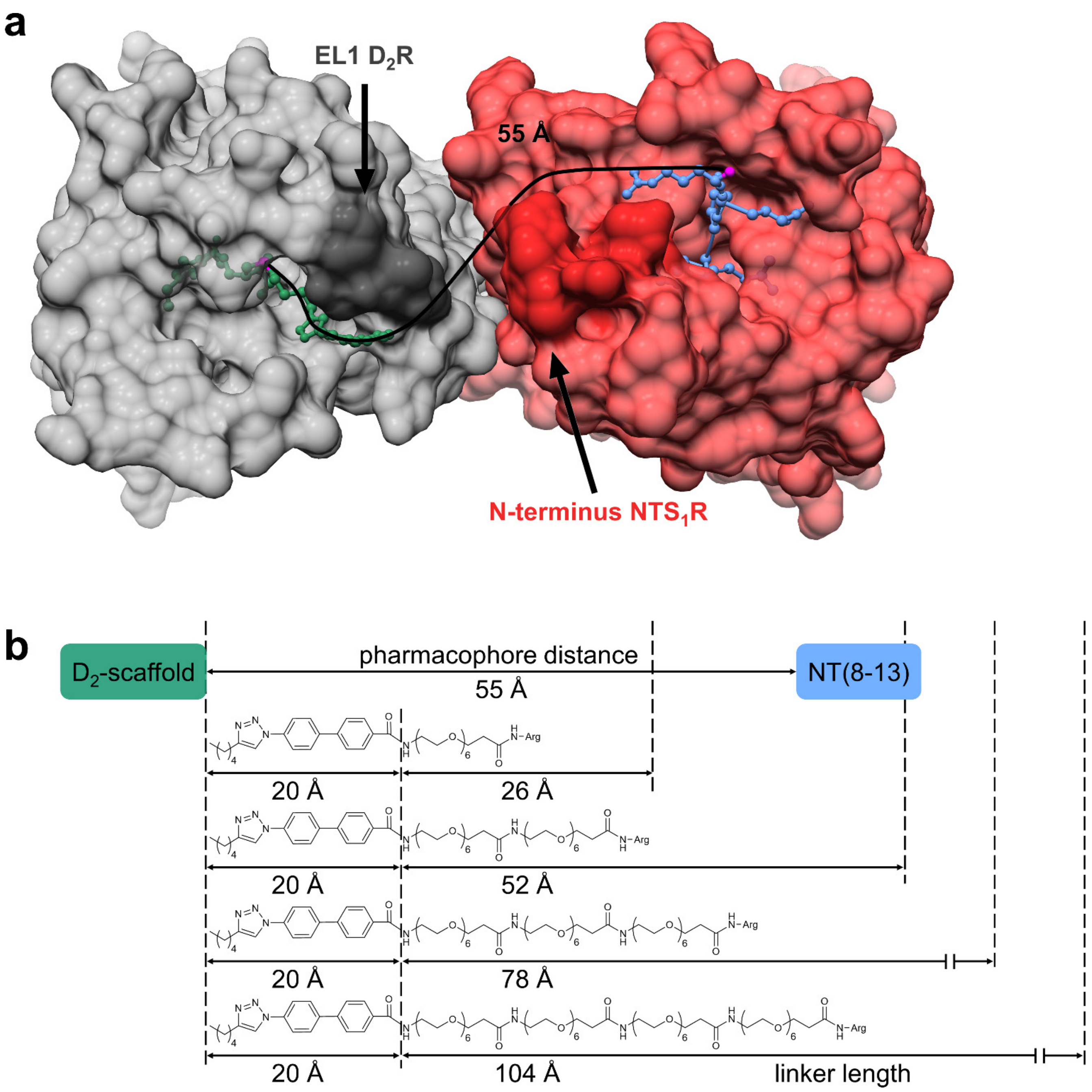


**Supplementary Figure 2. Distance between the eticlopride-type scaffold substituted with the lipophilic appendage and NT(8-13) at the $D_2R/NTS_1R$ dimer model and the inter-scaffold distance compared to maximal spacer lengths.** (**a**) The surface of $D_2R$ and $NTS_1R$ is shown in grey and red, respectively. NT(8-13) (blue) and the docked eticlopride-type scaffold including the appendage (green) are shown in ball and sticks representation. The dimer model was built as described in the methods section. The binding pose of the eticlopride-type scaffold achieved by docking reveals that the biphenyltriazol-based attachment is not facing straight towards $NTS_1R$ and that the way from the terminal phenyl group of the attachment to NT(8-13) is partially blocked by the extracellular loop 1 (EL1) of $D_2R$ and the N-terminus of $NTS_1R$. Therefore, the estimated distance between the connection points of the two pharmacophores is about 55 Å (connection points highlighted in pink). (**b**) Schematic comparison of the measured inter-scaffold distance and the maximal spacer lengths. The comparison indicates that two PEG-spacer units are required to bridge both orthosteric binding sites.

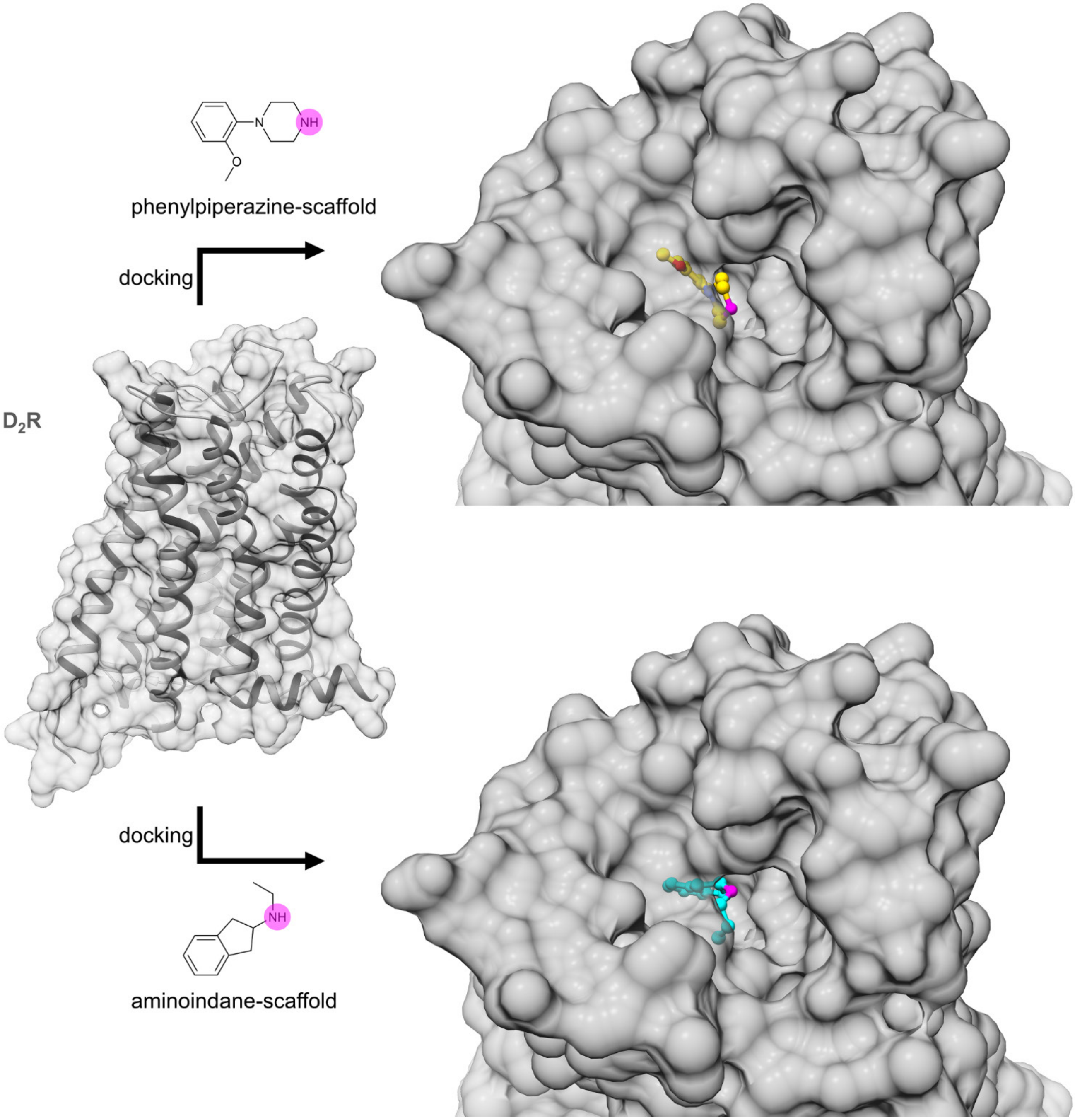


**Supplementary Figure 3. Attachment points of phenylpiperazine and aminoindane-type scaffolds determined by docking studies.** Ribbons and surface of $D_2R$, the structures of the phenylpiperazine- (top) and the aminoindane-type scaffold (bottom) are shown in grey, yellow and cyan, respectively. Positions of the two scaffolds were achieved by docking both compounds into our recently described $D_2R$ homology model (left)[1]. Docking studies were performed as described in the methods section. In both cases, the basic nitrogen (highlighted in pink in all representations) is accessible from the extracellular side of the receptor and therefore was selected as attachment point for the lipophilic appendage for compounds of type 2 and 3.

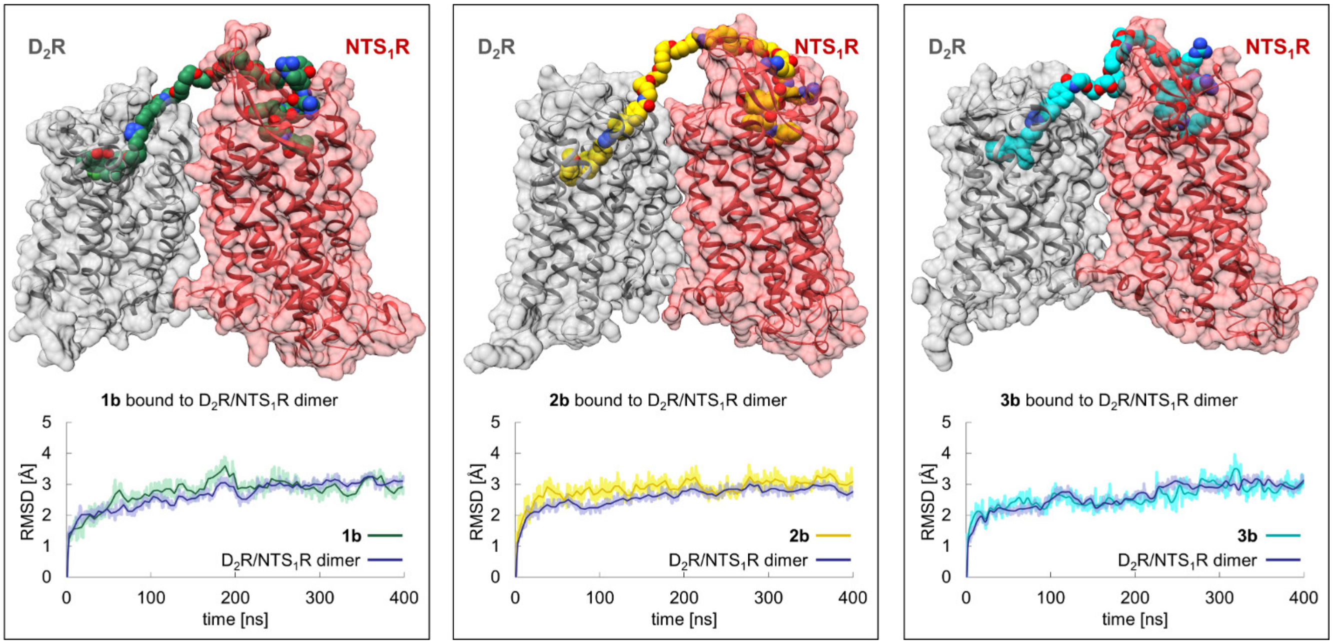


**Supplementary Figure 4. Molecular dynamics simulations of 1b, 2b and 3b bound to $D_2R/NTS_1R$ dimer.** Representative snapshots from molecular dynamics simulations of **1b** (left), **2b** (middle) and **3b** (right) in complex with our $D_2R/NTS_1R$ dimer model are shown (average structures taken from 250-400 ns, 200-400 ns and 250-400 ns, respectively). Ribbons and surfaces of the $D_2R$ and $NTS_1R$ are displayed in grey and red, respectively, with the ligands **1b**, **2b** and **3b** as spheres representation and highlighted in green, yellow and cyan. The RMS-deviations for the heterodimer and the ligands in the three system are displayed separately. The ligand-receptor-complexes were fitted on the $C\alpha$-atoms of the receptor. All values are shown as moving averages, in which each data point represents the mean of 10 ns. Unsmoothed values are displayed in lighter colors. All three simulation systems showed a formation of stable ligand-receptor complexes, which encouraged use to further evaluate compounds of type 1, 2 and 3 experimentally. Energy minimized representative structures of all three ligand-receptor complexes are supplied as Supplementary Data 1-3.

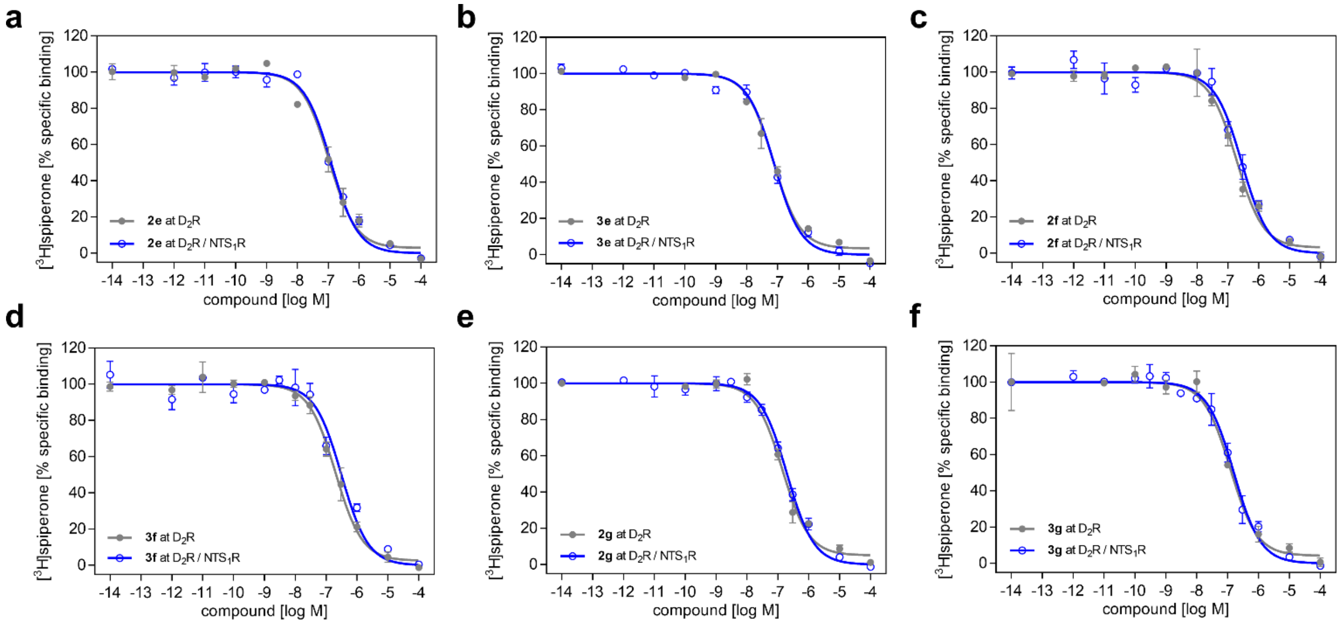


**Supplementary Figure 5: Competition binding experiments evaluating $D_2R$ affinities for compounds 2e-g, 3e-g.** Dopamine receptor binding of newly sythesized ligands was investigated by radioligand displacement with membranes from HEK 293T cells expressing only $D_2R$ (filled grey circles) or coexpressing $D_2R/NTS_1R$ (open blue circles). (**a-d**) Bivalent compounds comprising a peptoid-peptide hybrid instead of NT(8-13) (**2e**/**f**, **3e**/**f**, m=1 and 2, n=4 for all experiments) do not exhibit bivalent binding modes as indicated by the absence of biphasic competition binding curves. (**e**,**f**) Typical monophasic binding curves are observed for the monovalent ligands **2g**/**3g** (n=3 for $D_2R$, n=6 for $D_2R/NTS_1R$). Data points represent mean ± s.e.m. of *n* independent experiments, each performed in triplicate.

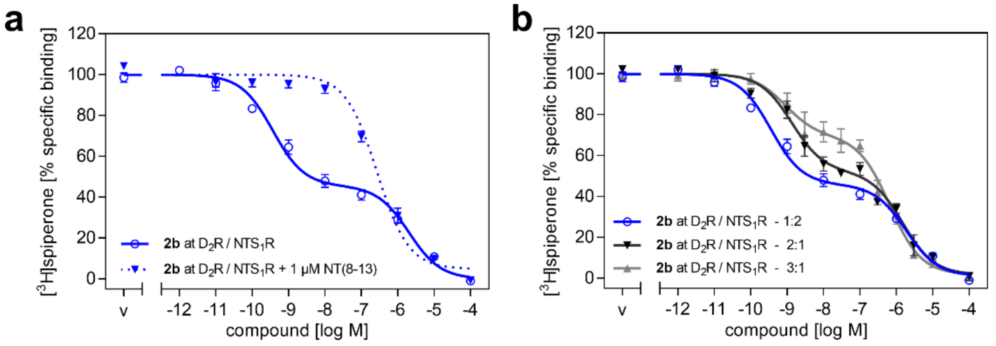


**Supplementary Figure 6: Binding behavior of bivalent ligand 2b in presence of NT(8-13) and with membranes coexpressing $D_2R/NTS_1R$ in different stoichiometry.** (**a**) Competition binding curves of ligand **2b** at the coexpressed receptors $D_2R$ and $NTS_1R$ were obtained in the absence (open blue circles) and presence (filled blue inverted triangles) of 1 µM NT(8-13). The type 2 ligand **2b** shows biphasic binding behaviour at the coexpressed receptor system $D_2R/NTS_1R$ with a $K_{i\ high}$ of 0.21 ± 0.07 nM and $K_{i\ low}$ of 630 ± 100 nM (n=11). In the presence of 1 µM NT(8-13) bivalent binding is completely abolished by blocking the $NTS_1R$ protomer by the monovalent NT(8-13) leading to a monophasic sigmoid competition curve with a $K_i$ value of 85 ± 18 nM (n=6). (**b**) Representative binding behaviour of the bivalent ligand **2b** at the coexpressed receptors $D_2R$ and $NTS_1R$ at different stoichiometry of receptor density. While **2b** shows the typical biphasic binding curve with a fraction of high affinity binding of 55 ± 3 % at a coexpression system with $D_2R/NTS_1R$ = 1:2 (open blue circles) (n=11, $K_{i\ high}$ 0.21 ± 0.07 nM, $K_{i\ low}$ 630 ± 100 nM), a 4- and 6-fold relative increase of expressed $D_2R$ attenuates the number of $D_2R/NTS_1R$ heterodimers. This is shown by a reduction of the high affinity binding sites to 49 ± 3 % ($D_2R/NTS_1R$ = 2:1 (black inverted triangles), n=3, $K_{i\ high}$ 0.42 ± 0.21 nM, $K_{i\ low}$ 260 ± 90 nM) and 30 ± 3% ($D_2R/NTS_1R$ = 3:1 (grey triangles), n=5, $K_{i\ high}$ 0.45 ± 0.37 nM, $K_{i\ low}$ 140 ± 30 nM). Data points represent mean ± s.e.m. of *n* independent experiments, each performed in triplicate; v = vehicle (binding buffer).

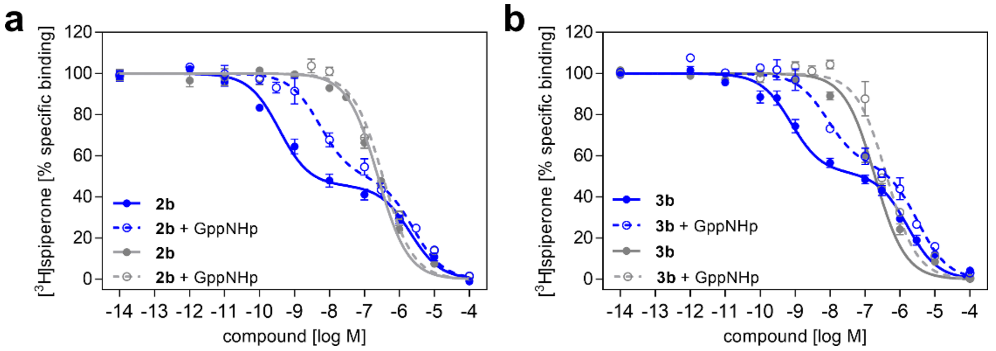


**Supplementary Figure 7: Influence of GppNHp on the binding behaviour of 2b and 3b.** Comparative receptor binding experiments investigating the influence of GppNHp on the binding behaviour were performed with the bivalent ligands **2b** (**a**) and **3b** (**b**) with $D_2R$ monoexpressing membranes (grey circles) or $D_2R/NTS_1R$ coexpressing homogenates (blue circles) in the absence (filled circles) or presence of 100 µM GppNHp (open cirles). For compound **2b**, binding to monoexpressed $D_2R$ is not significantly influenced by the presence of GppNHp ($K_{i\ -GppNHp}$ 42 ± 5 nM, n=11 vs. $K_{i\ +GppNHp}$ 45 ± 6 nM, n=6 ), whereas a slight rightward shift is observed for the bivalent ligand **3b**, bearing a $D_2R$ agonist pharmacophore ($K_{i\ -GppNHp}$ 36 ± 9 nM, n=12 vs. $K_{i\ +GppNHp}$ 68 ± 8 nM, n=6) For $D_2R/NTS_1R$ coexpressing membranes, biphasic curves were observed for **2b** and **3b** in presence and absence of GppNHp. However, addition of 100 µM GppNHp caused a rightward shift of the high affinity binding site for both bivalent ligands. The loss in binding affinity was 5.2-fold for **2b** ($K_{i\ high\ -GppNHp}$ 0.21 ± 0.07 nM, fraction high 50 ± 2 %, n=11 vs. $K_{i\ high\ +GppNHp}$ 1.1 ± 0.5 nM, fraction high 46 ± 3 %, n=3) and 6.4-fold for **3b** ($K_{i\ high\ -GppNHp}$ 0.47 ± 0.14 nM, fraction high 55 ± 3 %, n=22 vs. $K_{i\ high\ +GppNHp}$ 3.0 ± 1.9 nM, fraction high 47 ± 5 %, n=3). Data points represent mean ± s.e.m. of *n* independent experiments, each performed in triplicate.

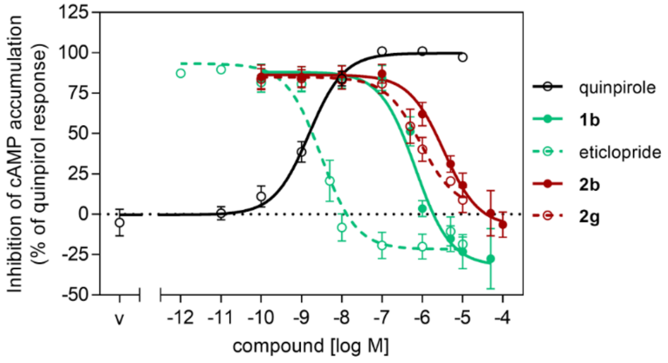


**Supplementary Figure 8: Antagonist effect on quinpirole-mediated inhibiton of cAMP accumulation.** Inhibition of quinpirole induced effect on cAMP accumulation by bivalent ligands **1b**/**2b** and monovalent control compounds. When HEK 293T cells expressing $D_2R$ receptors were treated with 10 nM quinpirole (~$EC_{80}$) and rising concentrations of eticlopride (open green circles) and **1b** (filled green circles), the quinpirole effect was completely abolished. Both test compounds acted as inverse agonists, decreasing the basal response by 22 % and 32 % with an apparent $IC_{50}$ of 3.1 and 680 nM, respectively. Albeit at lower potency, the two phenylpiperazines **2b** (filled red circles) and **2g** (open red circles) inhibited the quinpirole mediated response without any influence on the basal cAMP level, thus acting as neutral antagonists. Data points represent mean ± s.e.m. of 4 (3 for quinpirole) individual experiments, each performed in triplicate.

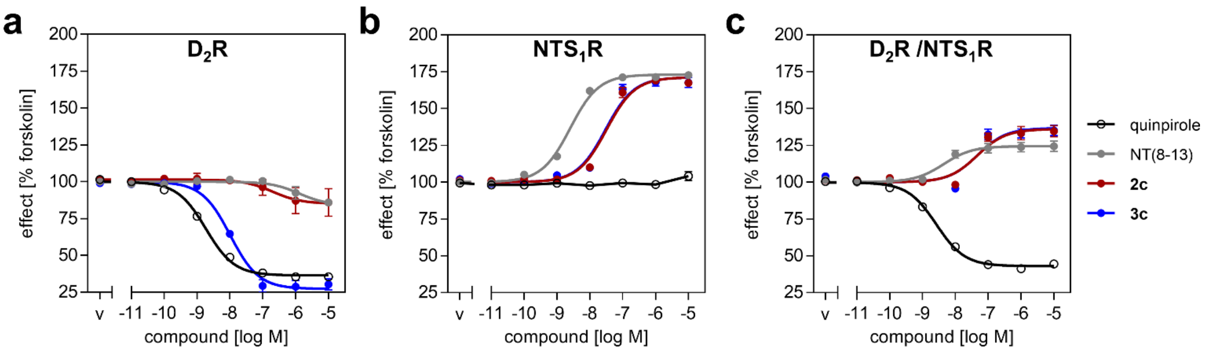


**Supplementary Figure 9: Activation profiles for of the bivalent ligands 2c and 3c in a cAMP accumulation assay with the BRET biosensor CAMYEL.** Activation profiles for of the bivalent ligands **2c** (n=3) and **3c** (n=4) in comparison to the reference agonists quinpirole (n=11) and NT(8-13) (n=11) at cells monoexpressing $D_2R$ (**a**) or $NTS_1R$ (**b**) and coexpressing $D_2R$ and $NTS_1R$ (**c**) and the cAMP-BRET biosensor CAMYEL. (**a**) While **3c** (blue circles) bearing the dopamine agonist pharmacophore stimulates $D_2R$ similar to quinpirole (open blac circles), **2c** (red circles) with the phenylpiperazine-pharmacophore only weakly activates $D_2R$. (**b**) $NTS_1R$ expressing cells were fully activated by both bivalent ligands (**2c** and **3c**) similar to the effect of NT(8-13) (grey circles). (**c**) In coexpressing cells, **2c** and **3c** behave similar to NT(8-13) indicating that $NTS_1R$ stimulation prevents the $G\alpha_{i/o}$-coupled inhibitory effect on cAMP production by $D_2R$. Data points represent mean ± s.e.m. of *n* independent experiments, each performed in triplicate.

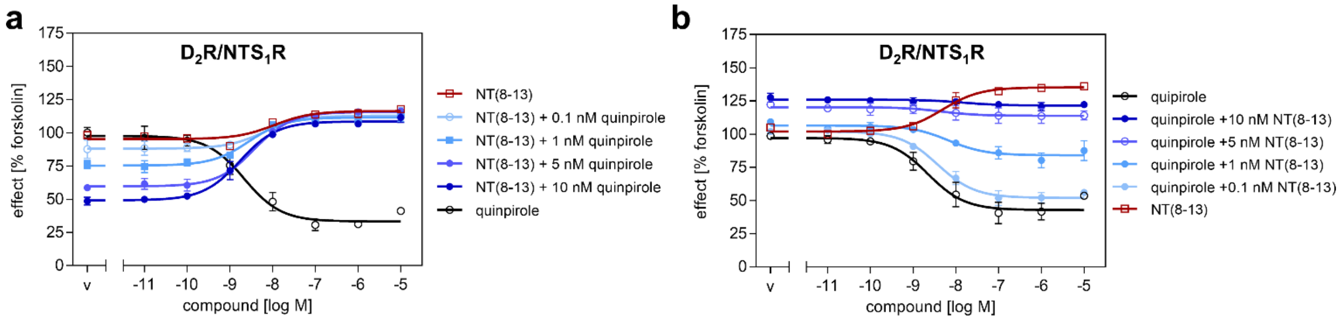


**Supplementary Figure 10: Activation profiles of the reference compounds quinpirole and NT(8-13) at the cAMP accumulation assay with the cAMP sensor CAMYEL.** HEK 293T cells transiently transfected with the cAMP biosensor (CAMYEL), $D_2R$ and $NTS_1R$ were stimulated with increasing concentrations of quinpirole and NT(8-13) as well as NT(8-13) in presence of 0.1-10 nM quinpirole (**a**), or quinpirole in the presence of 0.1-10 nM NT(8-13) (**b**). BRET ratio was transformed into cAMP concentration when the efficacy was normalized relative to the effect of 10 µM forskolin (=100 %) and the unstimulated effect by buffer (=0 %). (**a**) While quinpirole alone decreased the intracellular cAMP level dose-dependently, application of NT(8-13) increased the intracellular cAMP level by G protein-mediated stimulation of adenylyl cyclase. Consequently, addition of 0.1-10 nM quinpirole to vehicle (dPBS + 10 µM forskolin) decreased the amount of intracellular cAMP. However, by titration of NT(8-13) this initial decrease could be overcompensated. (**b**) When the same experiment was repeated with constant amounts of NT(8-13) (0.1-10 nM), the quinpirole mediated effect was gradually diminished. Only at concentrations of 0.1 nM or 1 nM NT(8-13) quinpirole was able to decrease the cAMP concentration below the vehicle conditions. Data represent mean ± s.e.m. of three individual experiments, each performed in triplicate.

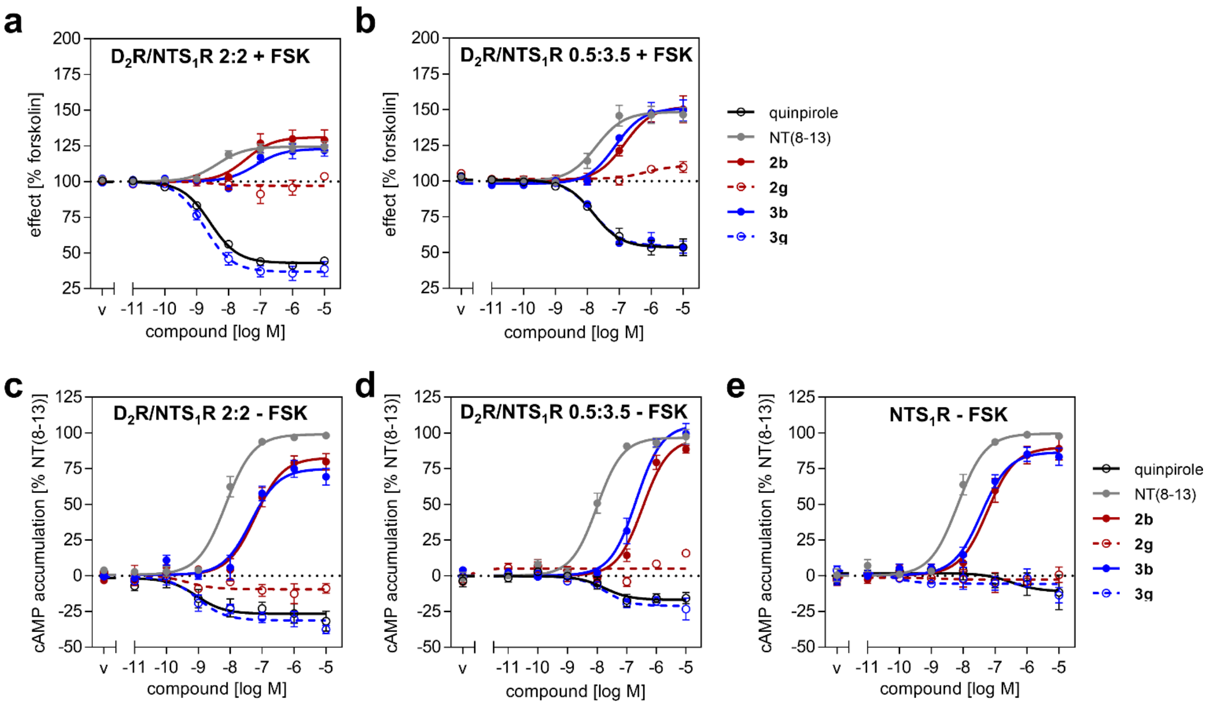


**Supplementary Figure 11: cAMP accumulation assay with ligands 2b, 3b and 2g, 3g under different conditions.** Activation profiles of the bivalent ligands **2b**, **3b** and their monovalent congeners **2g**, **3g** were determined at different ratios of $D_2R$/$NTS_1R$ expression or $NTS_1R$ monoexpression in presence (**a**,**b**) or absence (**c-e**) of 10 µM forskolin (+/-FSK). (**a**) Although potent activation for the $D_2R$ mediated inhibition of cAMP accumulation is seen for the monovalent ligand **3g** (open blue circles) (n=5) and quinpirole (n=11), the bivalent analog **3b** (filled blue circles) leads to an $NTS_1R$ mediated increase of the intracellular cAMP concentration (n=4). For ligand **2g** (open red circles) (n=3) no significant influence on cAMP levels was observed, while the bivalent congener **2b** (filled red circles) (n=5) also induces an increase of cAMP comparable to the effect of NT(8-13) (n=11). (**b**) At higher expression levels of $NTS_1R$, the $G\alpha_s$-stimulated response is increased ($E_{max}$ 148 % vs 124 % for NT(8-13), n=5). However, activation profiles of ligands **2b**,**g** (n=3) and **3b**,**g** (n=4) remain unchanged relative to NT(8-13) and quinpirole (n=5). (**c**) In the absence of forskolin (n=4 for all compounds), $D_2R$ activation is only weakly detectable. Quinpirole and the monovalent $D_2R$-agonist **3g** reduce cAMP levels by -25 % and -31 % respectively. The phenylpiperazine **2g** has a marginal effect, it leads to a slight decrease in basal cAMP (-10 %) However the bivalent ligands **2b**, **3b** behave similar to NT(8-13), increasing the cAMP levels to 83 % and 75 % of the maximum effect. (**d**) Under conditions with increased $NTS_1R$ expression (n=3 for all compounds), cAMP responses are comparable to the effects seen in (**c**). (**e**) In cells expressing $NTS_1R$ only, the monovalent ligands quinpirole, **2g**, **3g** do not elicit any response. Bivalent ligands **2b** and **3b** are able to increase cAMP levels in a manner similar to NT(8-13) (n=4 for all compounds) in the absence of FSK. In presence of FSK data was normalized to vehicle (0 %) and the effect obtained by FSK (10 µM, 100 %), in absence of FSK data was normalized to vehicle (0 %) and the maximum effect obtained with NT(8-13) (100 %). Ratios indicate the relative amount of cDNAs used for transfection. Data represent mean ± s.e.m. of *n* independent experiments, each performed in triplicate.

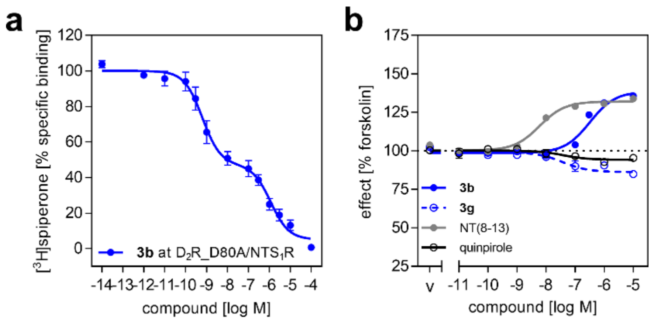


**Supplementary Figure 12: Binding profile and activation properties for the coexpression of $NTS_1R$ with a signalling deficient $D_2R$ mutant.** (**a**) Displacement of $[^3H]$spiperone from membranes of HEK 293T cells expressing $NTS_1R$ and a signalling incompetent $D_2R$ mutant (D80A) show biphasic binding behavior for the bivalent ligand **3b** ($K_{i\,high}$ 0.014 ± 0.02 nM, $K_{i\,low}$ 310 ± 30 nM) (**b**) When a signalling incompetent $D_2R$ (D80A) mutant is coexpressed with $NTS_1R$, quinpirole and **3g** are unable to inhibit cAMP accumulation. However, the activation profiles of NT(8-13) and the bivalent ligand **3b** remain unchanged compared to cells coexpressing wild type $D_2R/NTS_1R$. Data represent mean ± s.e.m. of 3 independent experiments, each performed in triplicate.

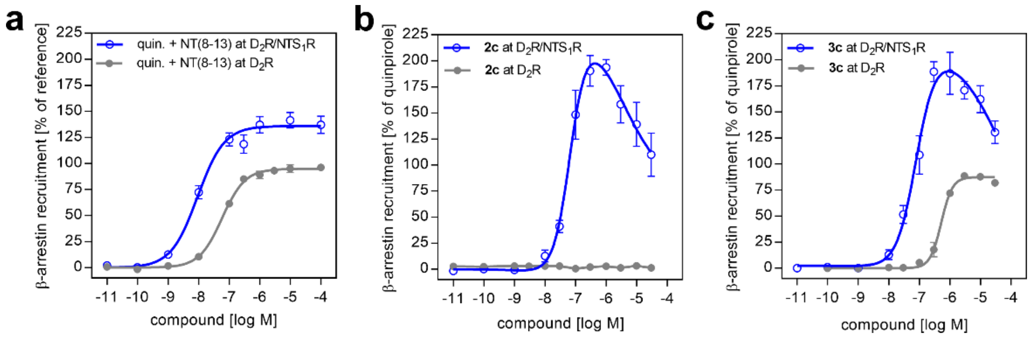


**Supplementary Figure 13: β-Arrestin-2 recruitment at $D_2R$ and $D_2R/NTS_1R$ induced by the bivalent ligands 2c and 3c and an equimolar combination of quinpirole and NT(8-13).** β-Arrestin recruitment was determined employing an assay based on enzyme complementation (DiscoveRx Pathhunter). HEK 293 cells stably expressing β-arrestin-2 tagged with the enzyme acceptor (EA) were transfected with ProLink™-tagged $D_2R$ with (open blue circles) or without (filled grey circles) cotransfection of $NTS_1R$. (**a**) When applied at equimolar concentration, quinpirole and NT(8-13) are able to stimulate β-arrestin-2 recruitment in $D_2R$ (n=4) and $D_2R/NTS_1R$ (n=6) expressing cells. Coexpression of $NTS_1R$ markedly increases the observed potency but also slightly the efficacy. (**b**) The bivalent ligand **2c** bearing a phenylpiperazine scaffold elicits β-arrestin-2 recruitment only in cells coexpressing $NTS_1R$ (n=3). The bell-shaped dose-response curve indicates a switch from bivalent to monovalent binding at concentrations >1 μM. (**c**) The aminoindane-derived bivalent ligand **3c** induces β-arrestin-2 recruitment in both cell types (n=4 for $D_2R$, n=3 for $D_2R/NTS_1R$). Coexpression of $NTS_1R$ potentiates the effect and leads to a bell-shaped dose-response curve with a maximum effect at 300 nM. Data represent mean ± s.e.m. of *n* independent experiments, each performed in duplicate. Results were normalized to the maximum effect of quinpirole (100 % for $D_2R$ and $D_2R/NTS_1R$).

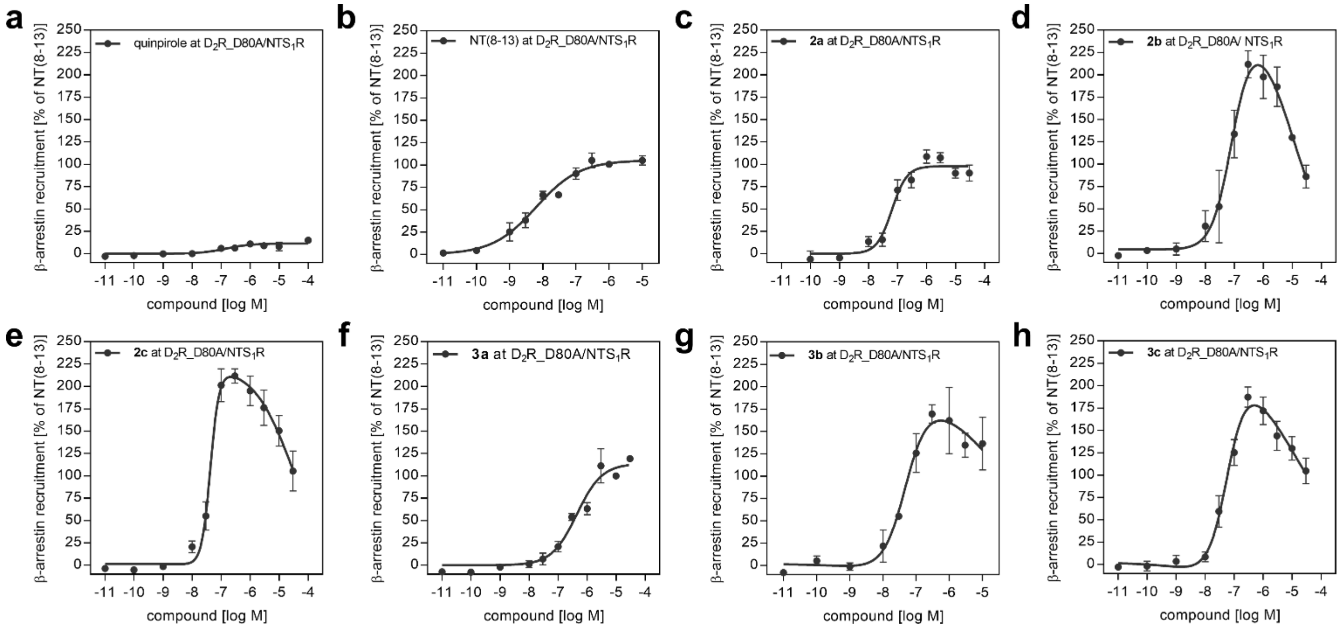


**Supplementary Figure 14: β-Arrestin-2 recruitment in HEK 293 cells coexpressing a signalling incompetent $D_2$R mutant (D80A) and $NTS_1$R.** β-Arrestin-2 recruitment was determined employing an assay based on enzyme complementation (DiscoveRx Pathhunter). HEK 293 cells stably expressing β-arrestin-2 tagged with the enzyme acceptor (EA) were transfected with a signalling incompetent ProLink™-tagged $D_2$R mutant (D80A) and $NTS_1$R (black circles). (**a**) Quinpirole is not able to induce significant recruitment of β-arrestin-2 when $D_2$R is signalling incompetent (n=6). (**b**) NT(8-13) elicits β-arrestin-2 recruitment at a similar potency compared to coexpression of wild type $D_2$R/$NTS_1$R ($EC_{50}$ 5.9 ± 1.2 nM, n=9). (**c**) The bivalent ligand **2a** with a 22-atom spacer displays a sigmoid dose-response curve with a maximum effect of 98 ± 4 % ($EC_{50}$ 67 ± 23 nM, n=4). (**d**,**e**) Increasing the spacer length to 44 or 66 atoms converts the sigmoid curves into bell-shaped dose-response relationships, where the maximum effect is observed at a concentration of 300 nM. Maximum effects are significantly higher as observed for NT(8-13), indicating a higher degree of heterodimerization (n=4 for **2b** and **2c**). (**f**) Similar to compound **2a**, ligand **3a** shows β-arrestin-2 recruitment to a similar extend as NT(8-13), albeit at lower concentration ($EC_{50}$ 190 ± 40 nM, n=4). (**g**, **h**) The bivalent ligands **3b** and **3c** bearing a $D_2$R agonist pharmacophore and a spacer length of 44 and 66 atoms, respectively, display bell-shaped dose-response curves (n=3 and 4 for **3b** and **3c**) similar to the type 2 ligands **2b** and **2c**. Data represent mean ± s.e.m. of *n* independent experiments, each performed in duplicate. Responses were normalized to the maximum effect obtained with NT(8-13) (100 %).

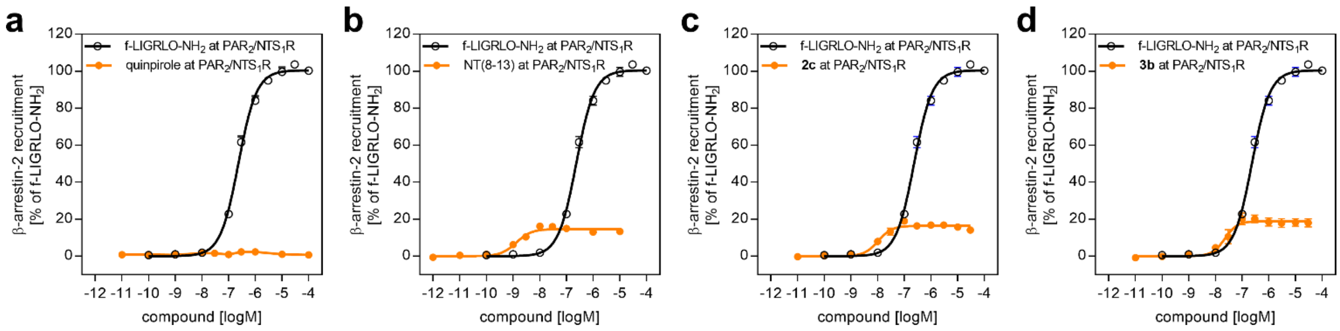


**Supplementary Figure 15: β-arrestin-2 recruitment in $PAR_2/NTS_1R$ coexpressing HEK 293 cells.** β-Arrestin-2 recruitment was determined employing the DiscoveRx Pathhunter assay. HEK 293 cells stably expressing β-arrestin-2 tagged with the enzyme acceptor (EA) were transfected with ProLink™-tagged $PAR_2$ and wild type $NTS_1R$. (**a**) The dopamine receptor agonist quinpirole (orange circles) does not cause any β-arrestin-2 recruitment compared to the $PAR_2$ agonist f-LIGRLO-$NH_2$ (open black circles). (**b-d**) NT(8-13) and the representative bivalent ligands **3b** and **2c** (orange circles) cause a weak recruitment of β-arrestin-2, ranging from 15 % to 19 % of the f-LIGRLO-$NH_2$ (open black circles) effect. Data represent mean ± s.e.m. of 7 independent experiments, each performed in duplicate. Responses were normalized to the effect of the $PAR_2$-agonist f-LIGRLO-$NH_2$ (100%) and vehicle (0%).

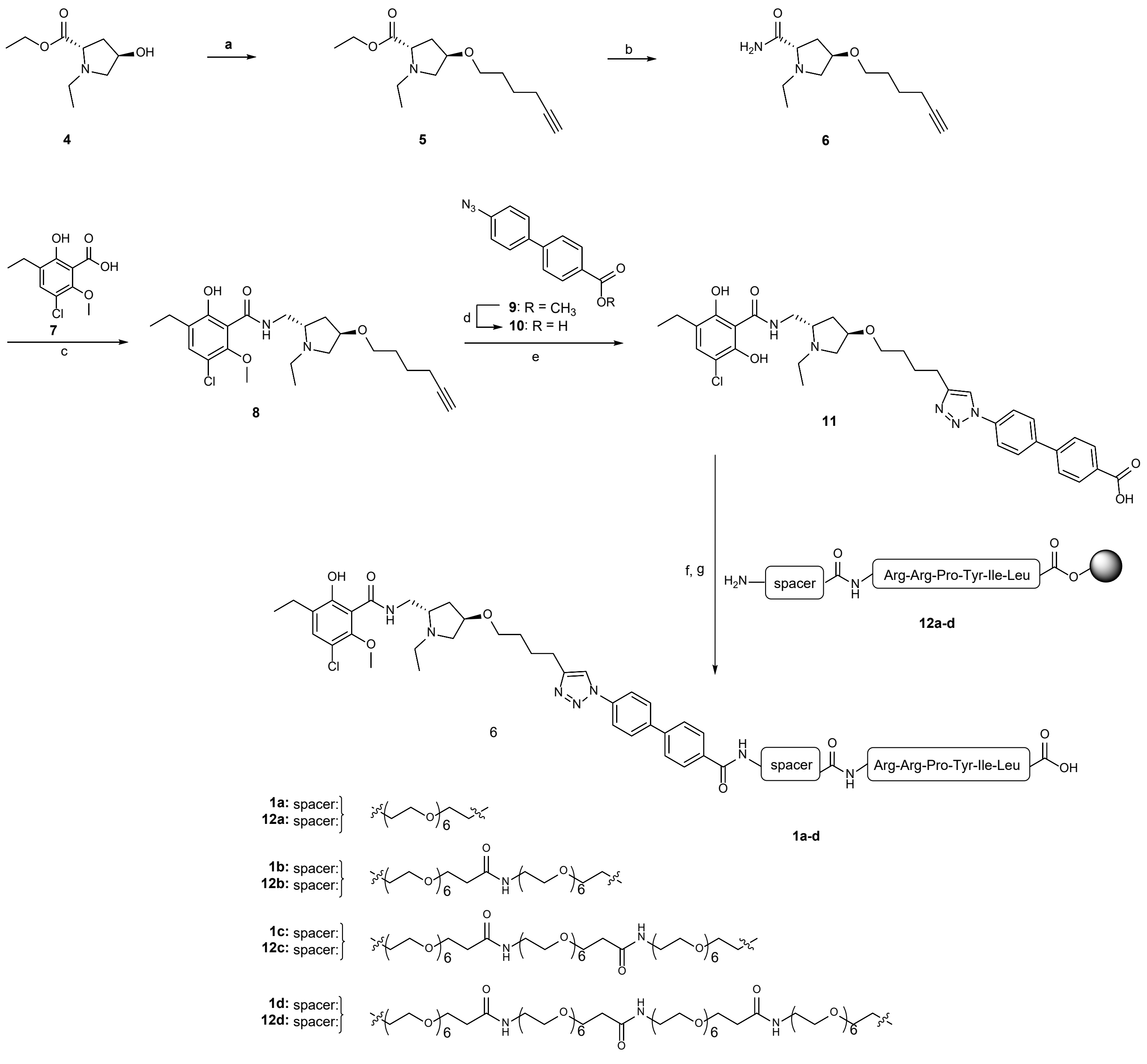


**a**, 6-Iodohex-1-yne, NaH, DMF, 50 °C, 18.5 h, 32-34 %; **b**, $NH_3$ / MeOH, KI, 40 °C, 14 d, 78 %; **c**, $LiAlH_4$, THF, reflux, 16 h, then **7**[2], HOBt, EDC HCl, $CH_2Cl_2$, 0 °C – RT, 17.5 h, then NaOH, MeOH, 40 °C, 1.5 h, 15-59 %; **d**, NaOH, EtOH / $H_2O$, reflux, 1h, quant.; **e**, $CuSO_4 \cdot 5\ H_2O$, Na-ascorbate, isopropanol / $CH_2Cl_2$ / $H_2O$, RT, 17 h, 78-85 %, **f**, PyBOP, HOBt, DIPEA, µ-wave; **g**, TFA / phenol / $H_2O$ / TIS, RT, 3 h.

**Supplementary Figure 16: Synthesis of the bivalent ligands 1a-d.** *O*-alkylation of **4** followed by subsequent amidation, $LiAlH_4$–reduction and coupling to **7**[2] yielded eticlopride derivative **8**, which was converted to triazole derivative **11** by an azide-alkyne cycloaddition. Attachment to compounds **12a-d**, which were synthesized via standard solid phase peptide synthesis on a Wang resin, was achieved employing PyBOP, HOBt and DIPEA. Cleavage of the resin and simultaneous deprotection of the amino acid side chains yielded final bivalent ligands **1a-d**.

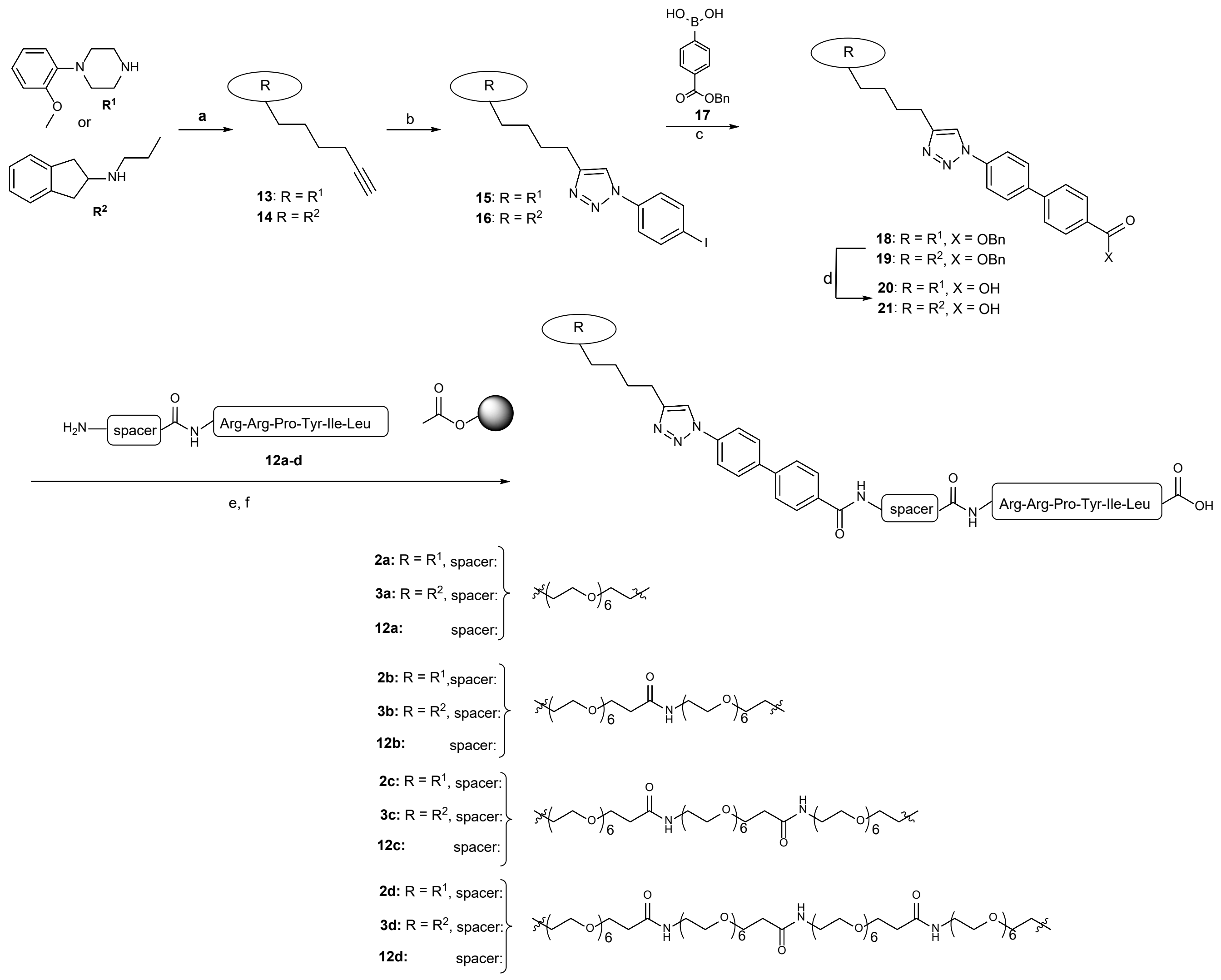


**a**, 6-Chlorohex-1-yne, $K_2CO_3$, KI, DMF, µ-wave: 120 °C, 1.5 h, 80-84 %; **b**, 1-azido-4-iodobenzol, $Cu(CH_3CN)_4PF_6$, $CH_2Cl_2$ / MeOH, µ-wave: 50 °C, 20 min, 90-94 %; **c**, 4-[(benzyloxy-carbonyl)phenyl]boronic acid **17**, PdEnCat30, $K_2CO_3$, DMF, MeOH, µ-wave: 100 °C, 0.5 h, 78- 86 %; **d**, Pd/C, $H_2$, MeOH/ $CH_2Cl_2$, RT, 48 h, 98 %; **e**, HATU, DIPEA, µ-wave; **f**, TFA / phenol / $H_2O$ / TIS, RT, 3 h.

**Supplementary Figure 17: Synthesis of the bivalent ligands 2a-d and 3a-d.** *N*-alkylation of the 1-(2-methoxyphenyl)piperazine (**R¹**) and *N*-propyl-2-aminoindane (**R²**) respectively, followed by a azide-alkyne cycloaddition, yielded the two triazole derivatives **15** and **16**. Following Suzuki-coupling with benzyl-protected 4-carboxyphenylboronic acid **17** and subsequent hydrogenolytic debenzylation gave the two required building blocks **20** and **21**. Attachment to compounds **12a-d**, which were synthesized via standard solid phase peptide synthesis on a Wang resin, was achieved with the coupling agent HATU. Cleavage of the resin and simultaneous deprotection of the amino acid side chains yielded final bivalent ligands **2a-d** and **3a-d**.

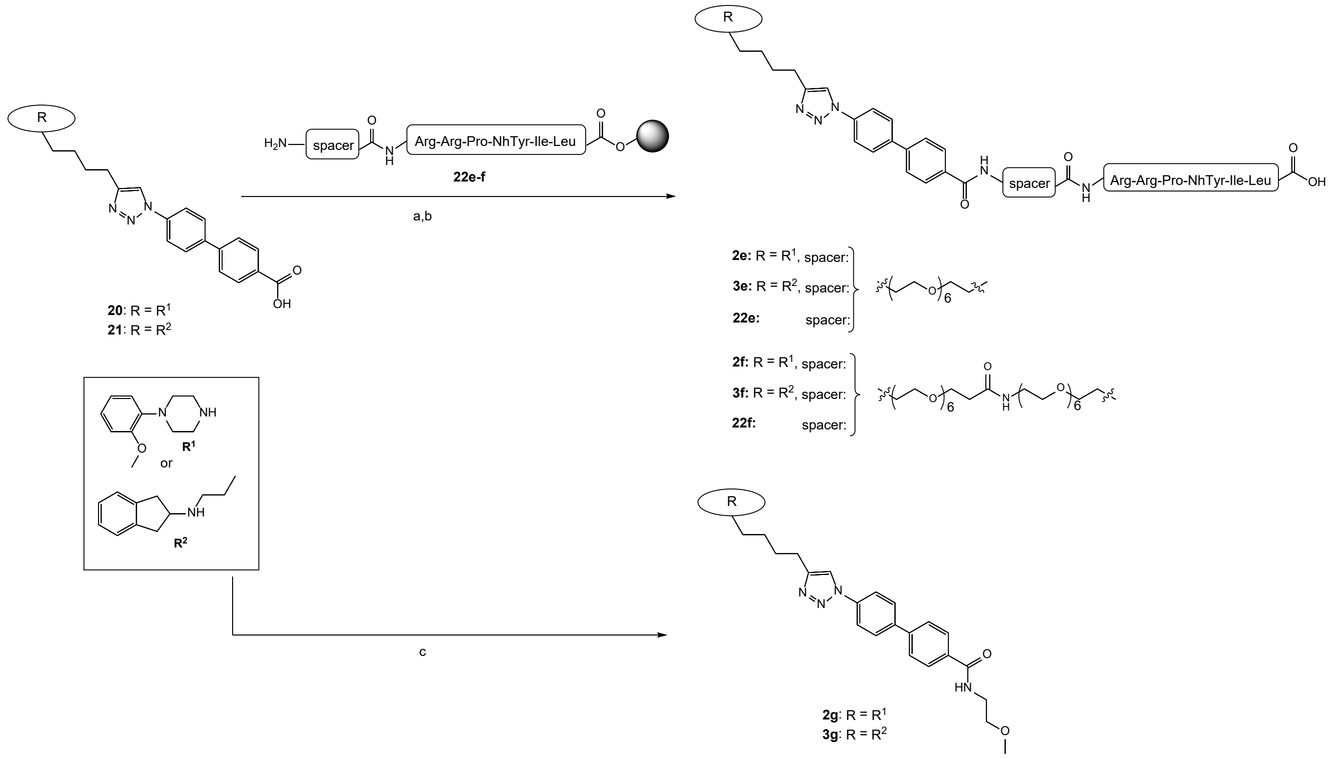


**a**, HATU, DIPEA, µ-wave; **b**, TFA / phenol / $H_2O$ / TIS, RT, 3 h; **c**, 2-methoxyethylamine, DIPEA, HATU, DMF, RT, 3 h.

**Supplementary Figure 18: Synthesis of the bivalent ligands 2e/f and 3e/f and the monovalent analogs 2g/3g.** For **2e**/**f** and **3e**/**f**, synthesis was carried out via standard solid phase peptide synthesis on a Wang resin. Attachment of the building blocks **20** and **21** to compounds **22e**/**f** was achieved with the coupling agent HATU. Cleavage of the resin and simultaneous deprotection of the amino acid side chains yielded final bivalent ligands **2e**/**f** and **3e**/**f**. Attachment of 2-methoxyethylamine to the building blocks **20** and **21** was achieved via amide-bond-formation with the coupling agent HATU yielding the two compounds **2g** and **3g**.

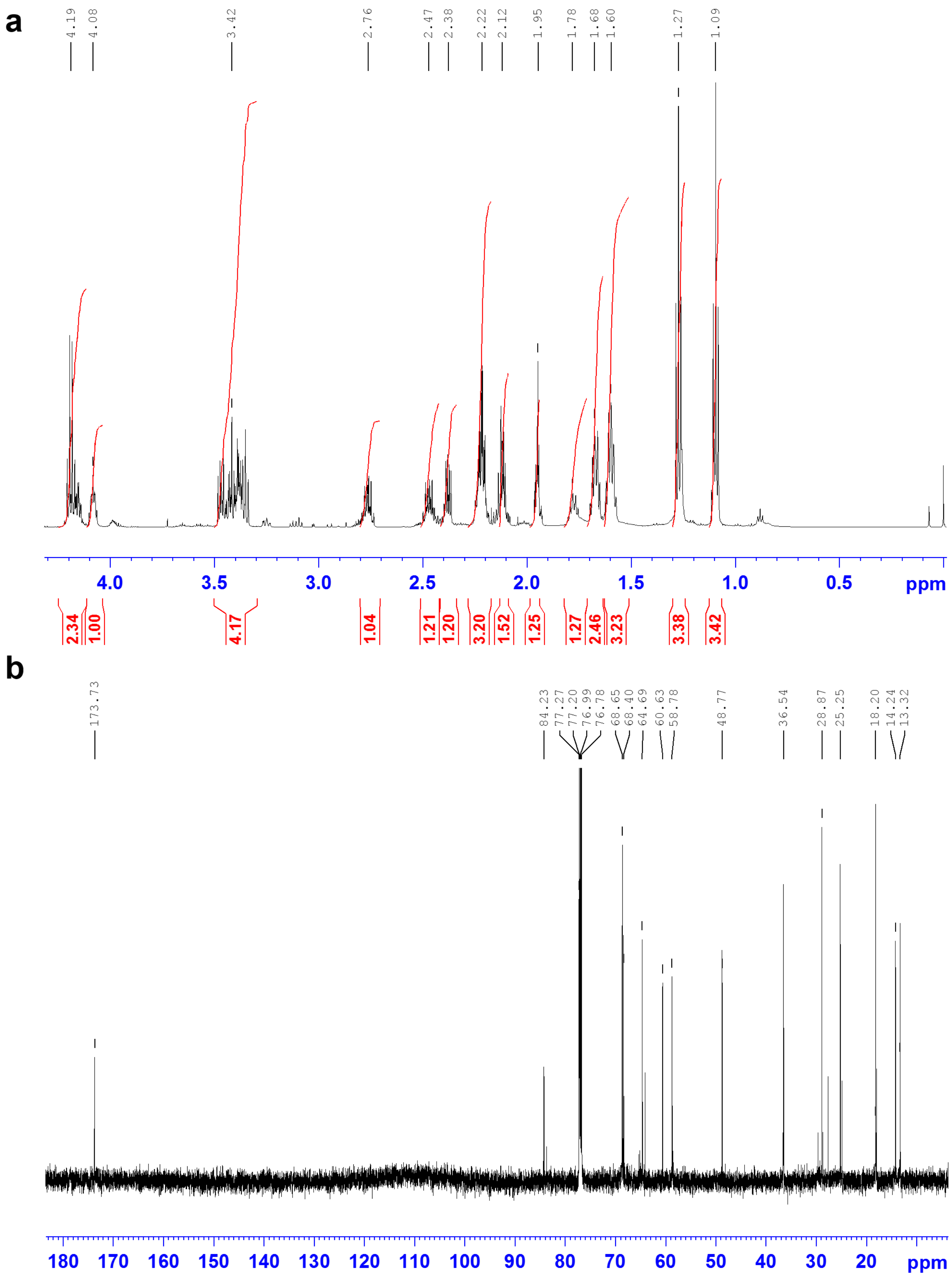


**Supplementary Figure 19: NMR spectra of compound 5.** (**a**) $^{1}H$ ($CDCl_3$, 600 MHz) and (**b**) $^{13}C$ ($CDCl_3$, 150 MHz).

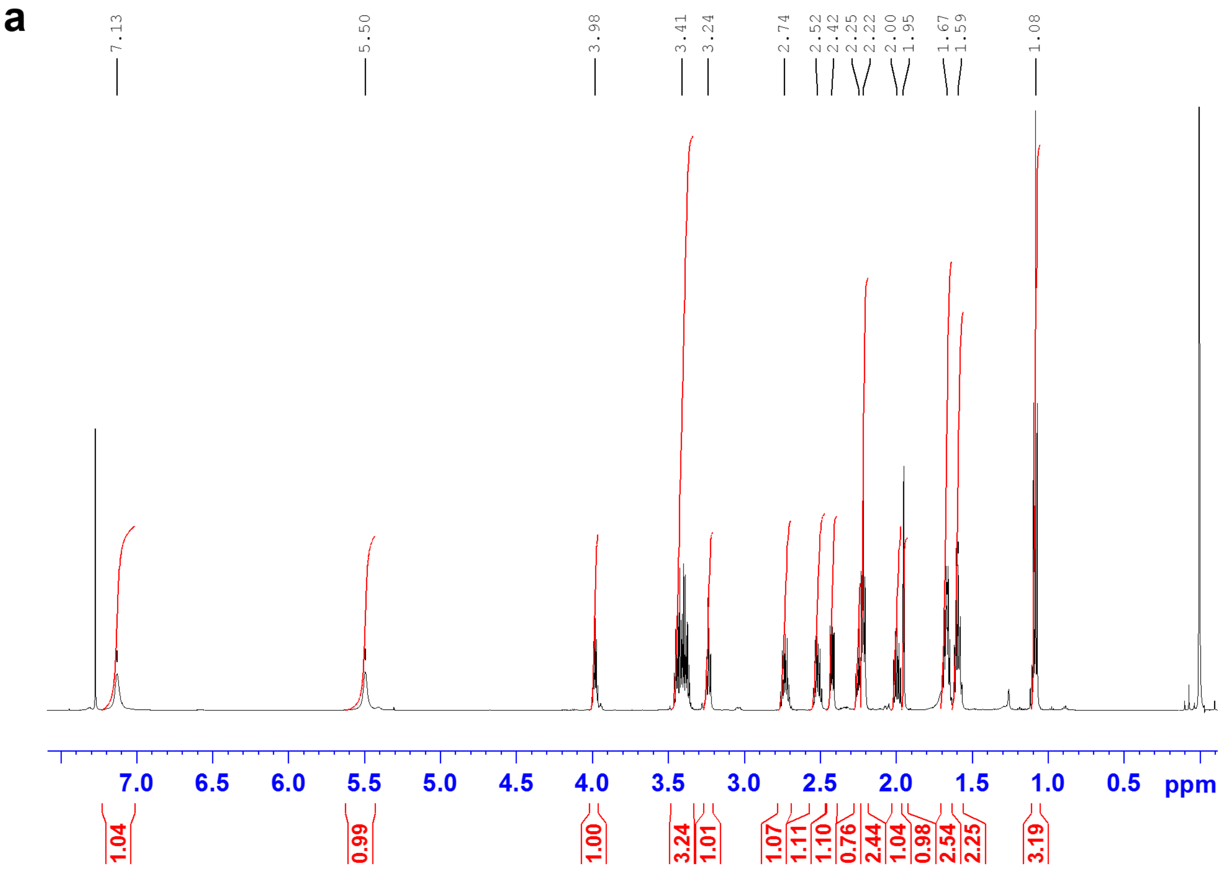


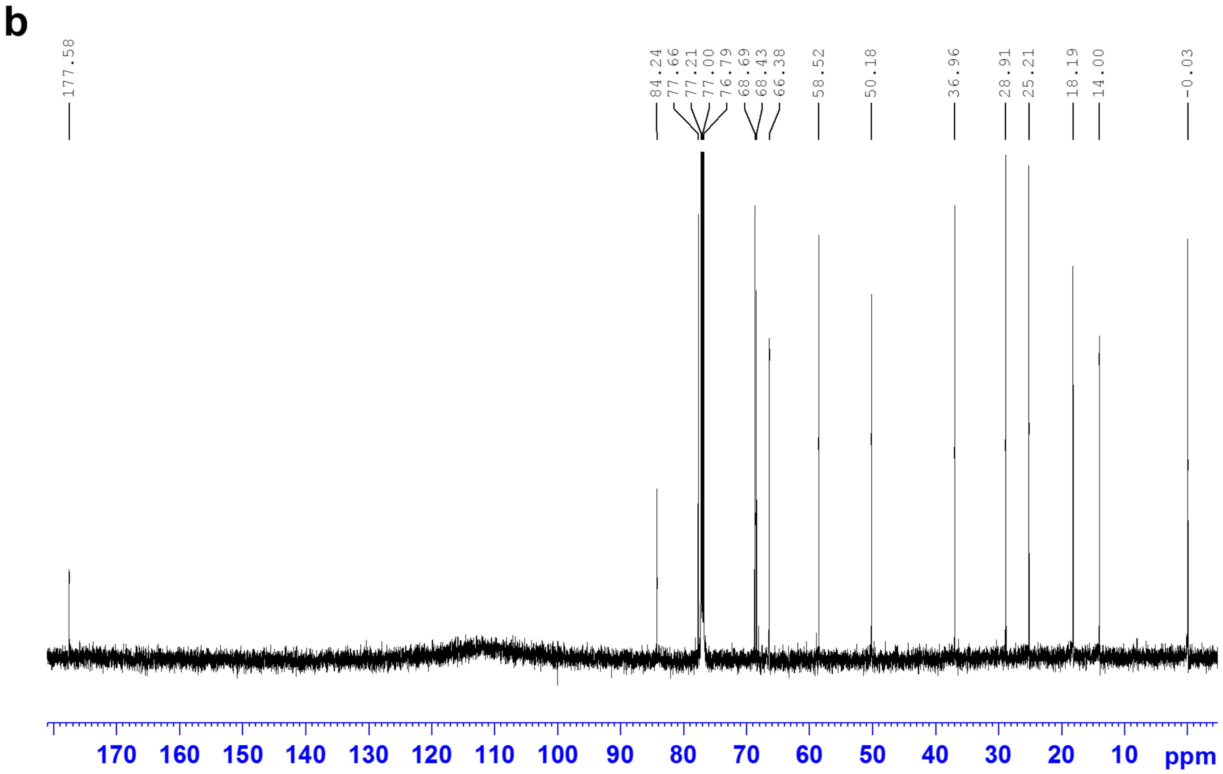


**Supplementary Figure 20: NMR spectra of compound 6.** (**a**) $^{1}H$ ($CDCl_3$, 600 MHz) and (**b**) $^{13}C$ ($CDCl_3$, 150 MHz).

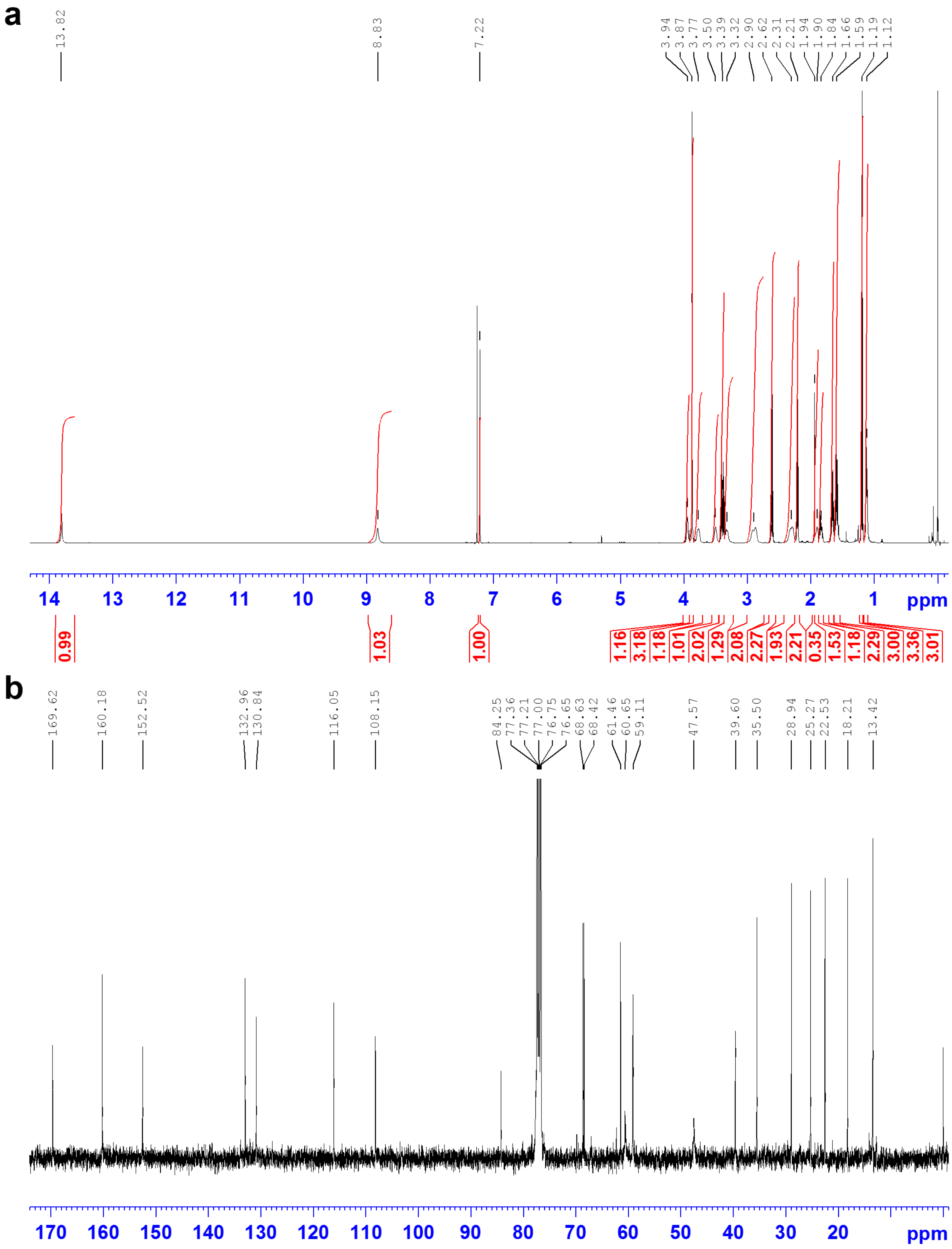


**Supplementary Figure 21: NMR spectra of compound 8.** (**a**) $^{1}H$ ($CDCl_3$, 600 MHz) and (**b**) $^{13}C$ ($CDCl_3$, 90 MHz).

a

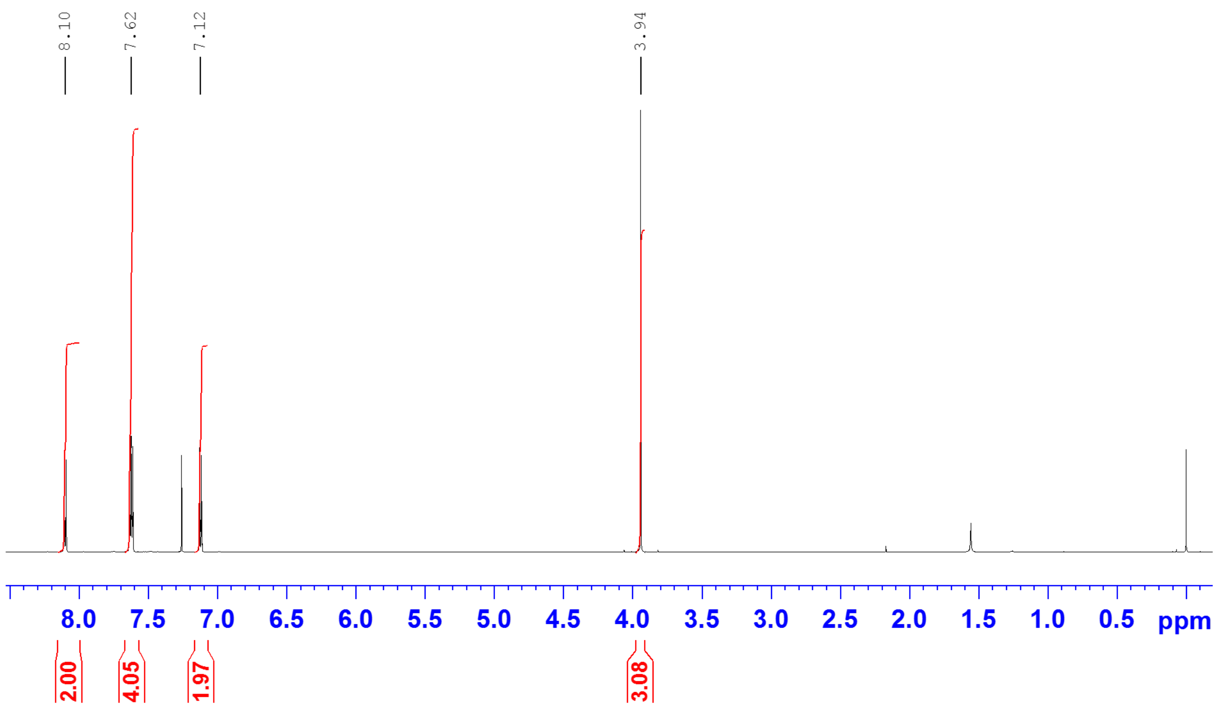


b

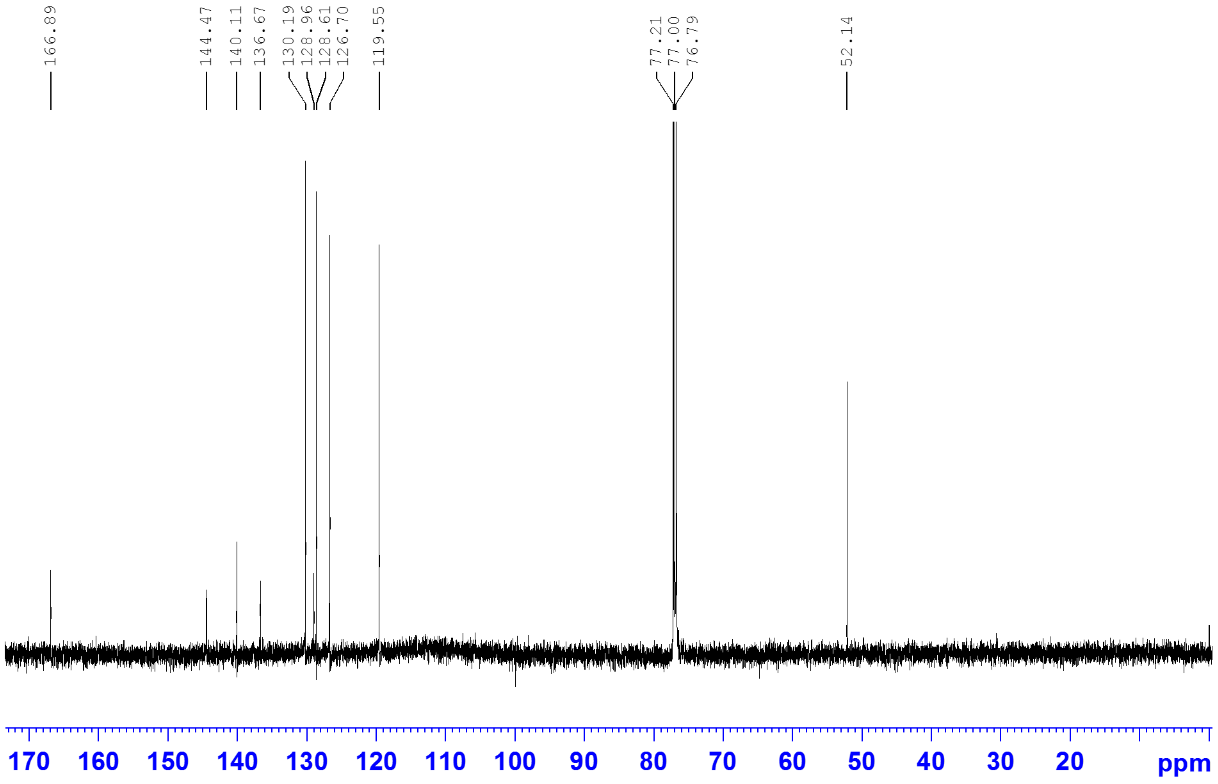


**Supplementary Figure 22: NMR spectra of compound 9.** (**a**) $^{1}H$ ($CDCl_3$, 600 MHz) and (**b**) $^{13}C$ ($CDCl_3$, 150 MHz).

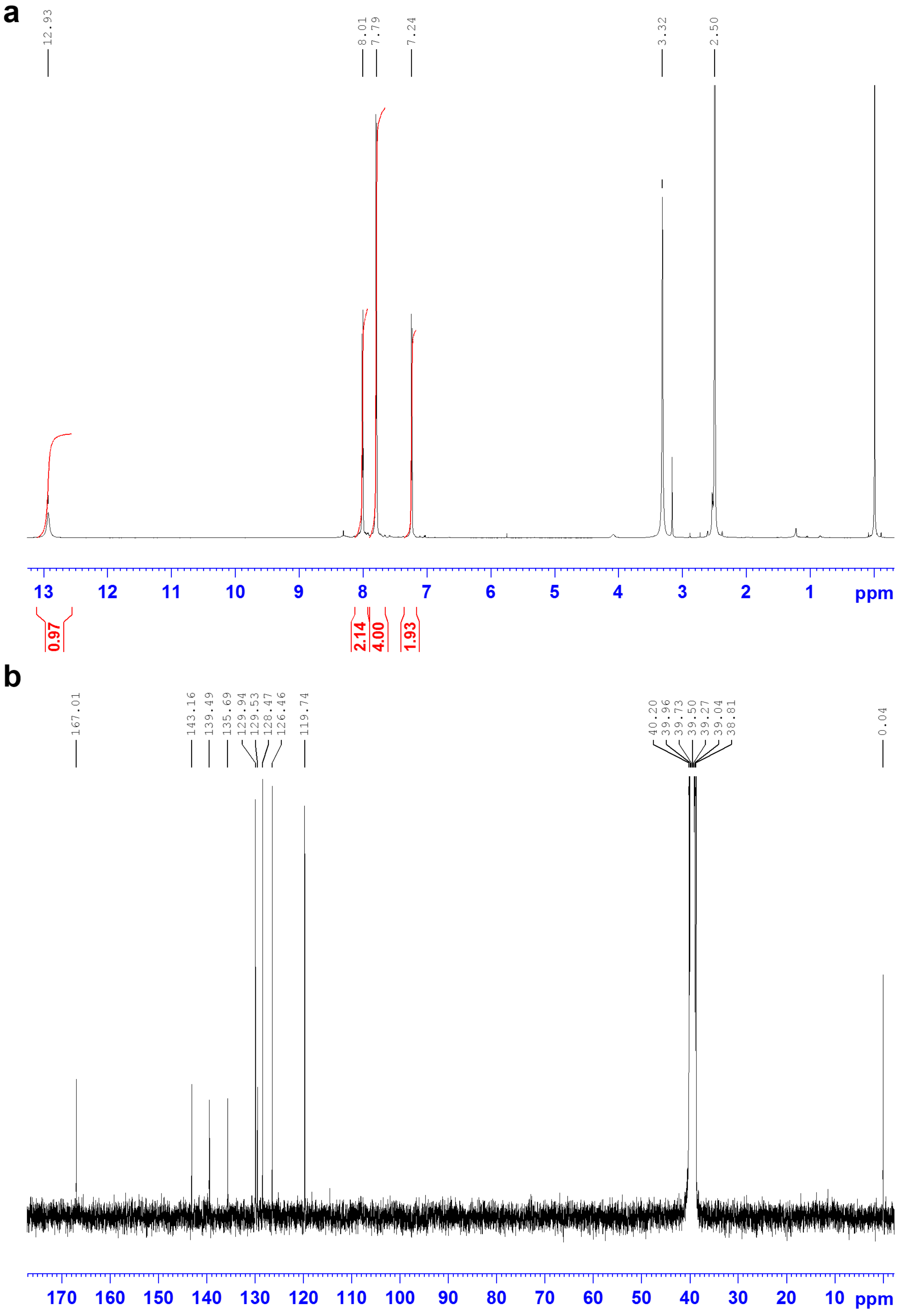


**Supplementary Figure 23: NMR spectra of compound 10.** (**a**) $^{1}H$ (DMSO-$d_6$, 600 MHz) and (**b**) $^{13}C$ (DMSO-$d_6$, 90 MHz).

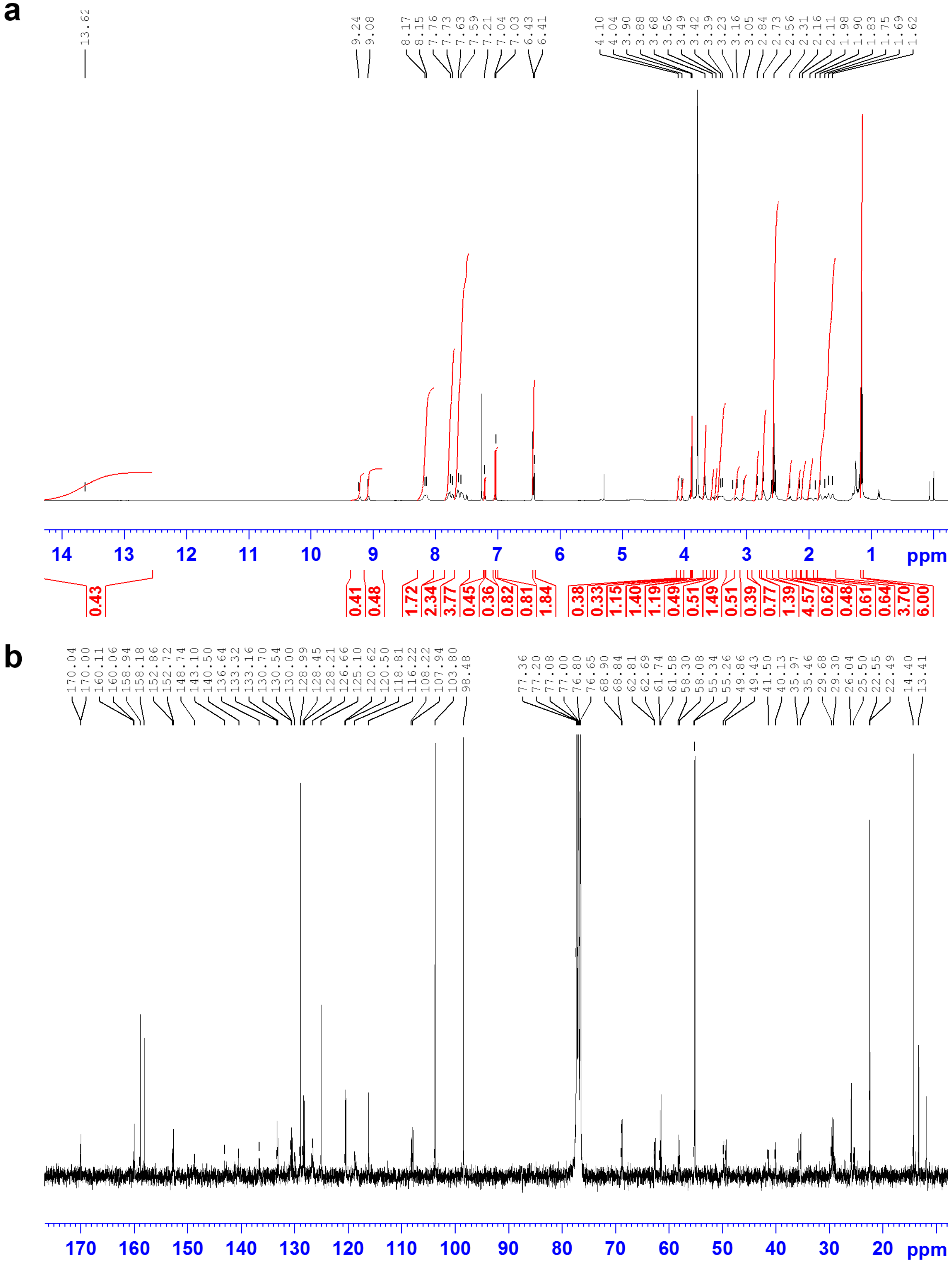


**Supplementary Figure 24: NMR spectra of compound 11.** (**a**) $^{1}H$ ($CDCl_3$, 600 MHz) and (**b**) $^{13}C$ ($CDCl_3$, 90 MHz).

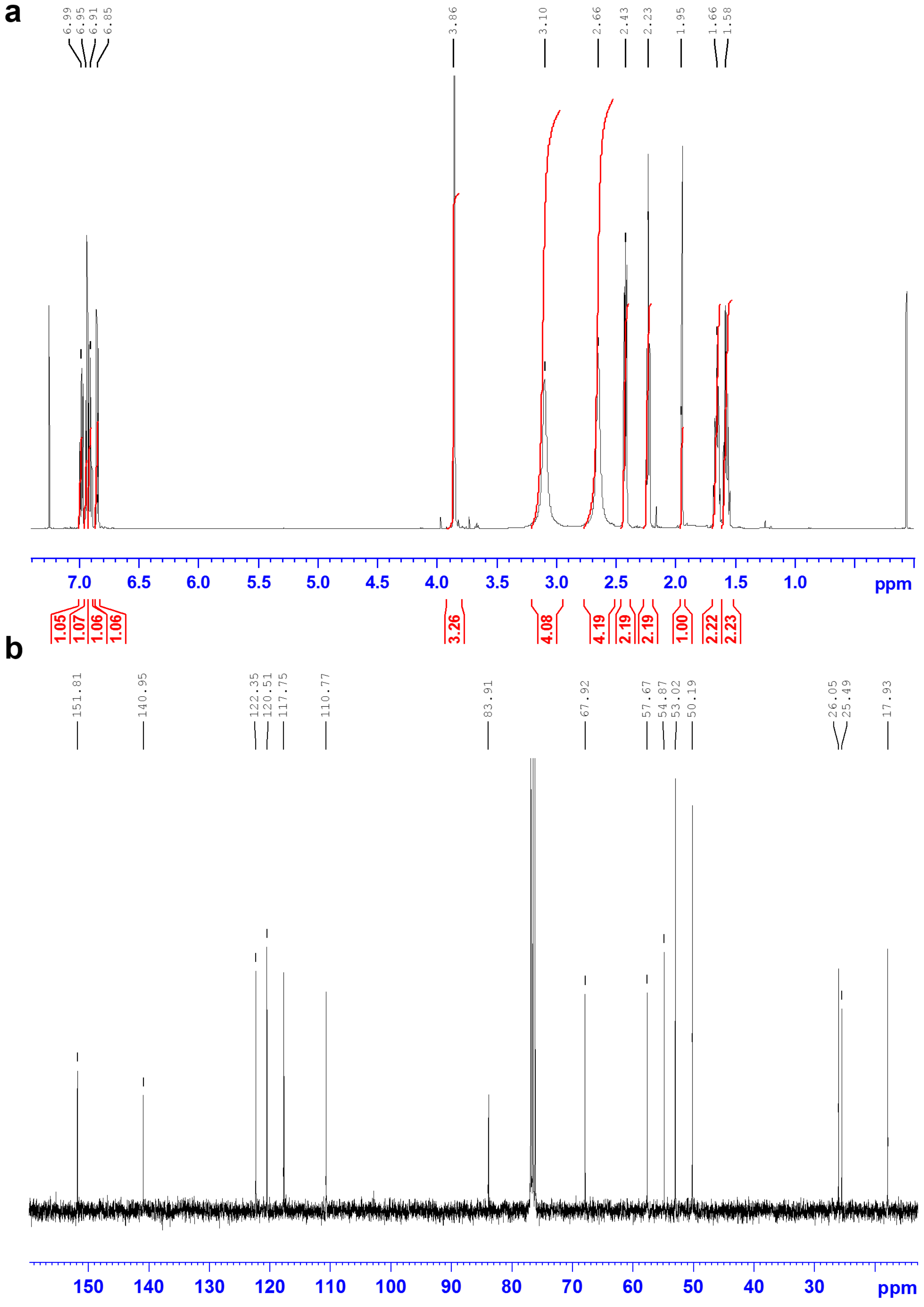


**Supplementary Figure 25: NMR spectra of compound 13.** (**a**) $^{1}H$ ($CDCl_3$, 600 MHz) and (**b**) $^{13}C$ ($CDCl_3$, 90 MHz).

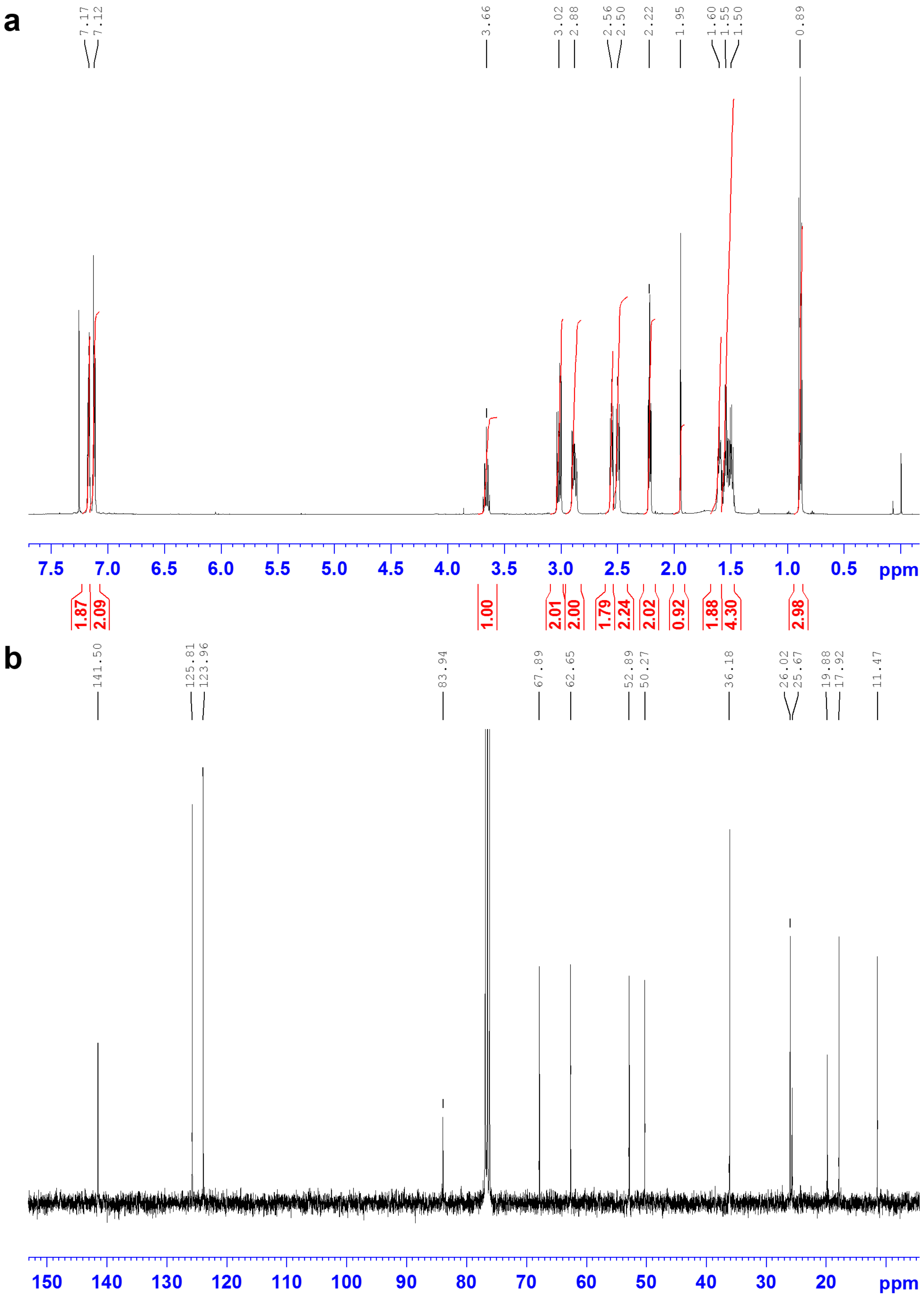


**Supplementary Figure 26: NMR spectra of compound 14.** (**a**) $^{1}H$ ($CDCl_3$, 600 MHz) and (**b**) $^{13}C$ ($CDCl_3$, 90 MHz).

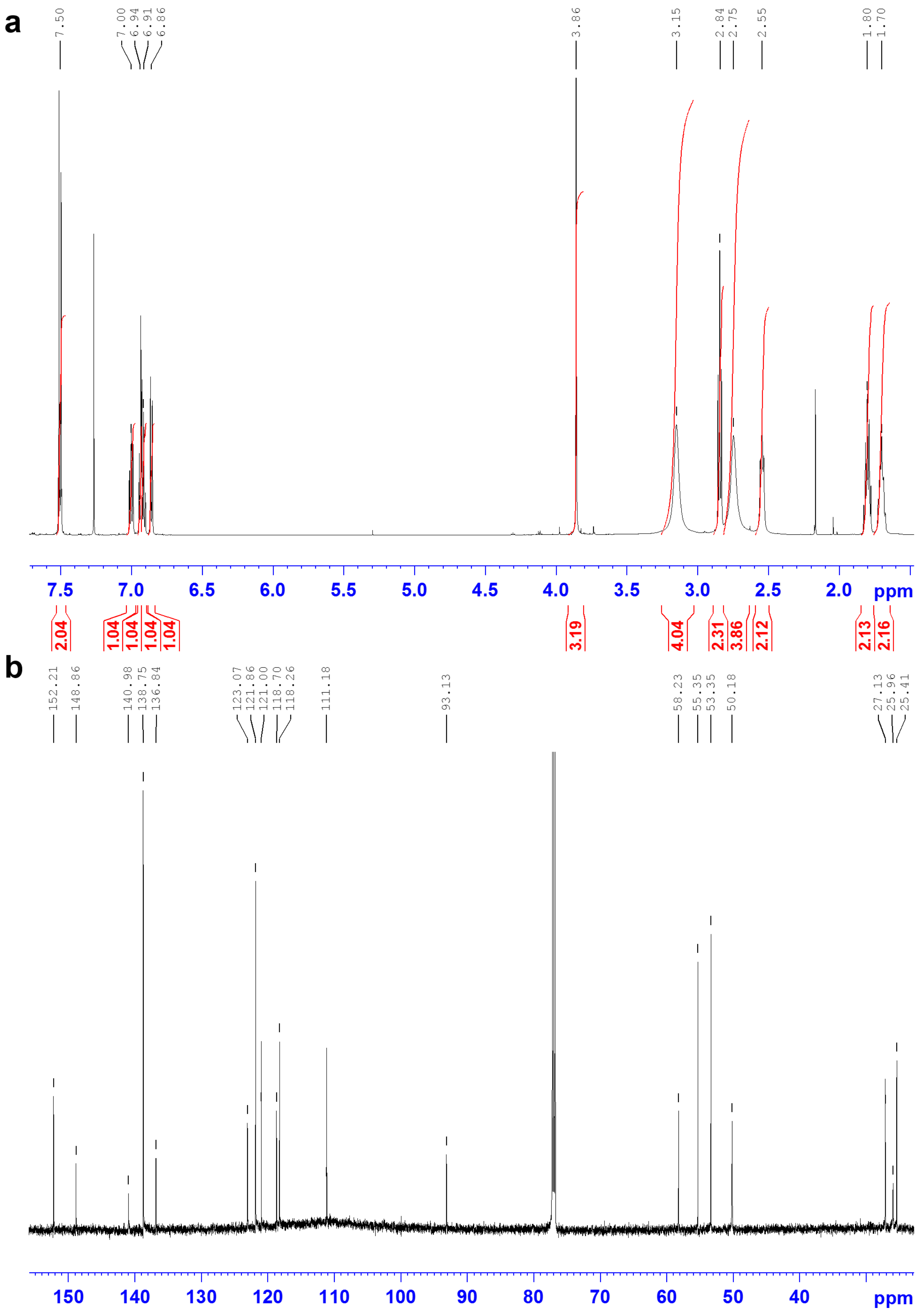


**Supplementary Figure 27: NMR spectra of compound 15.** (**a**) $^{1}H$ ($CDCl_3$, 600 MHz) and (**b**) $^{13}C$ ($CDCl_3$, 150 MHz).

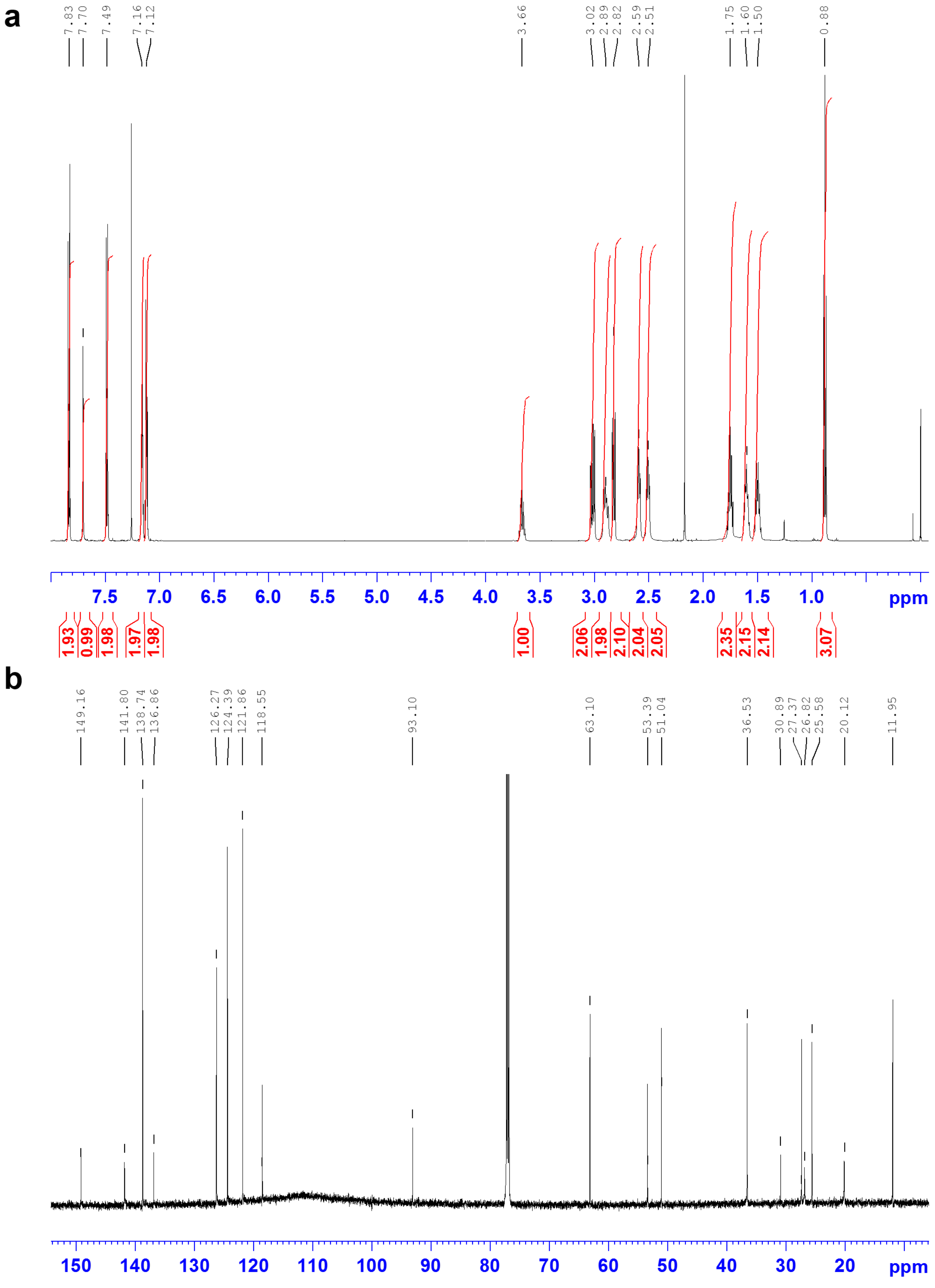


**Supplementary Figure 28: NMR spectra of compound 16.** (**a**) $^{1}H$ ($CDCl_3$, 600 MHz) and (**b**) $^{13}C$ ($CDCl_3$, 150 MHz).

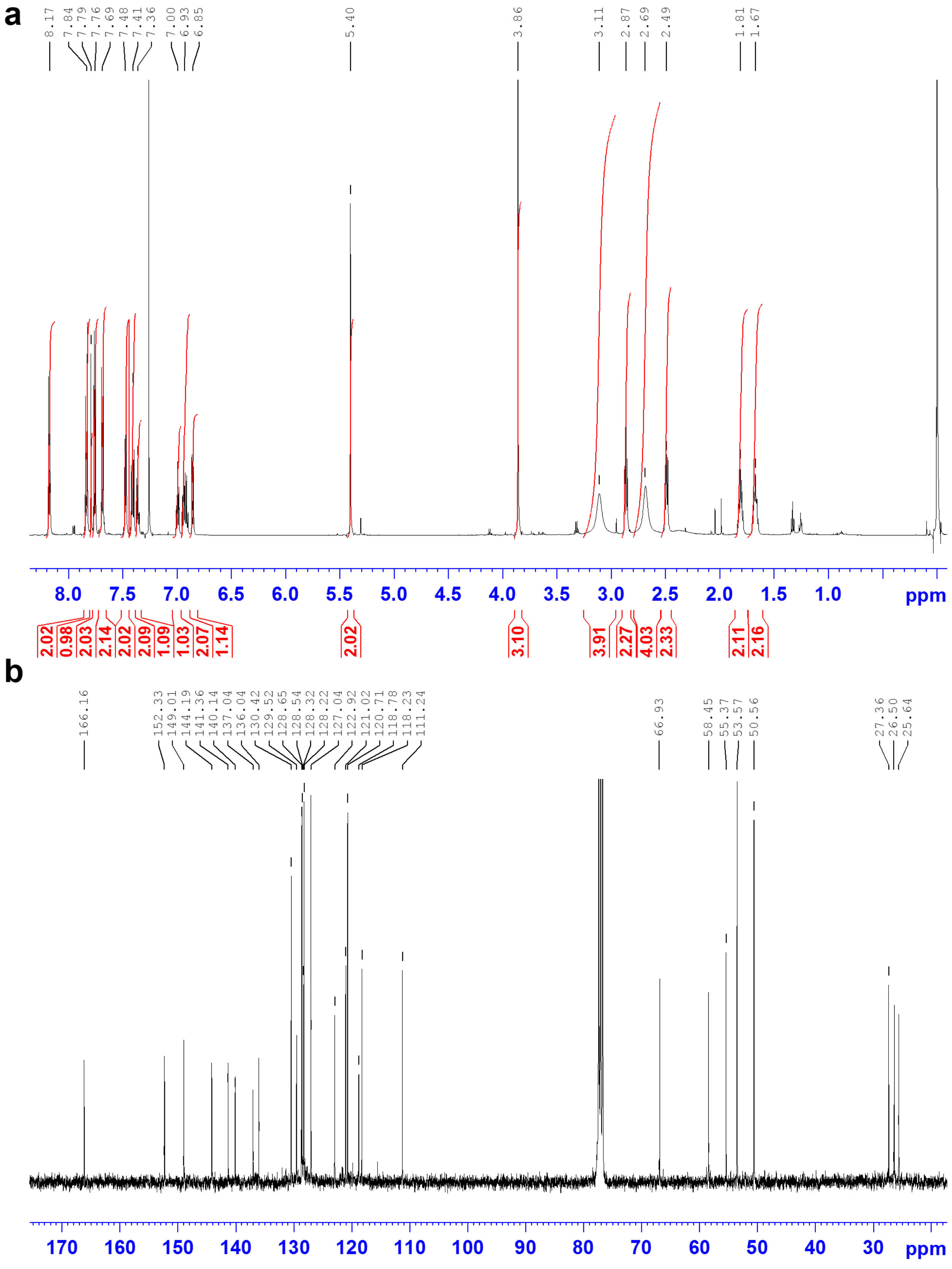


**Supplementary Figure 29: NMR spectra of compound 18.** (**a**) $^{1}H$ ($CDCl_3$, 600 MHz) and (**b**) $^{13}C$ ($CDCl_3$, 90 MHz).

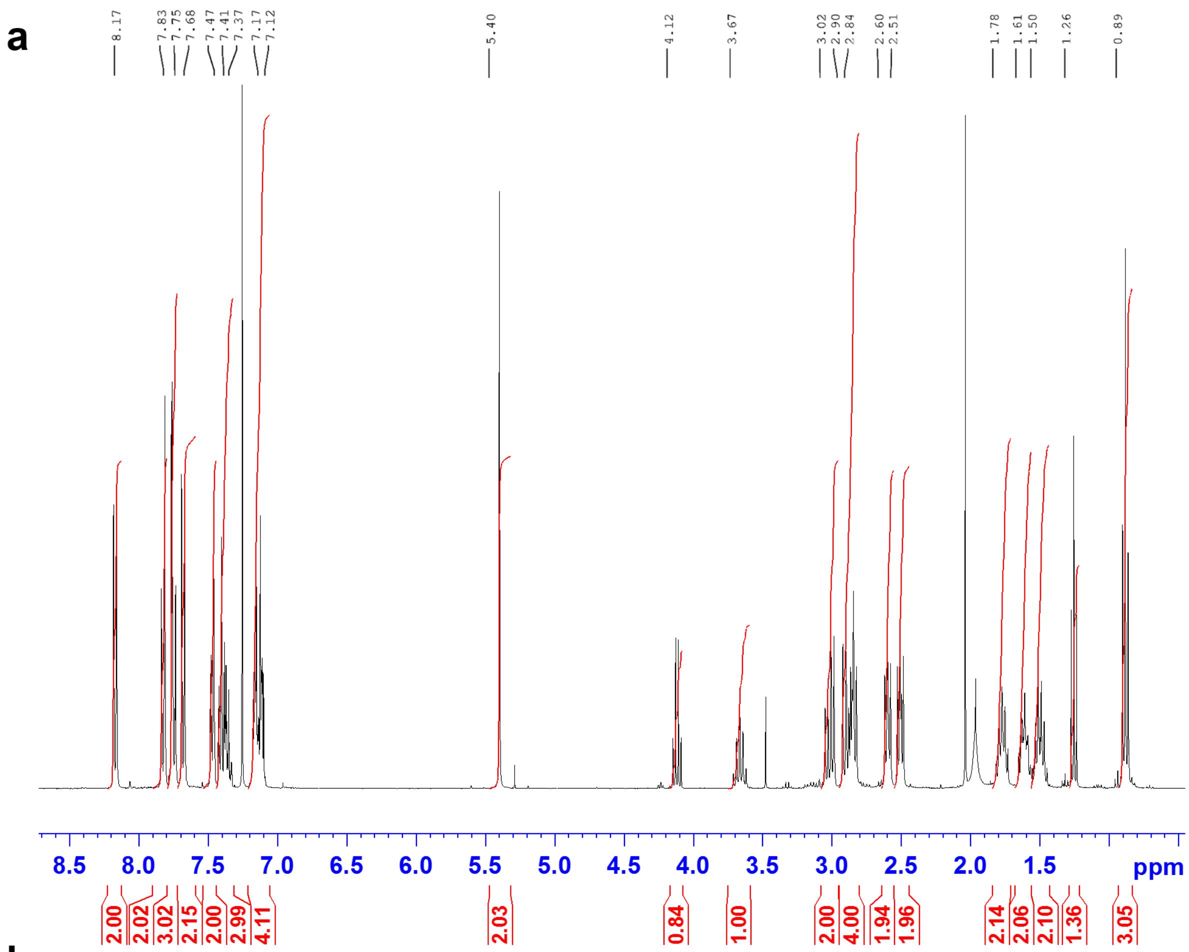


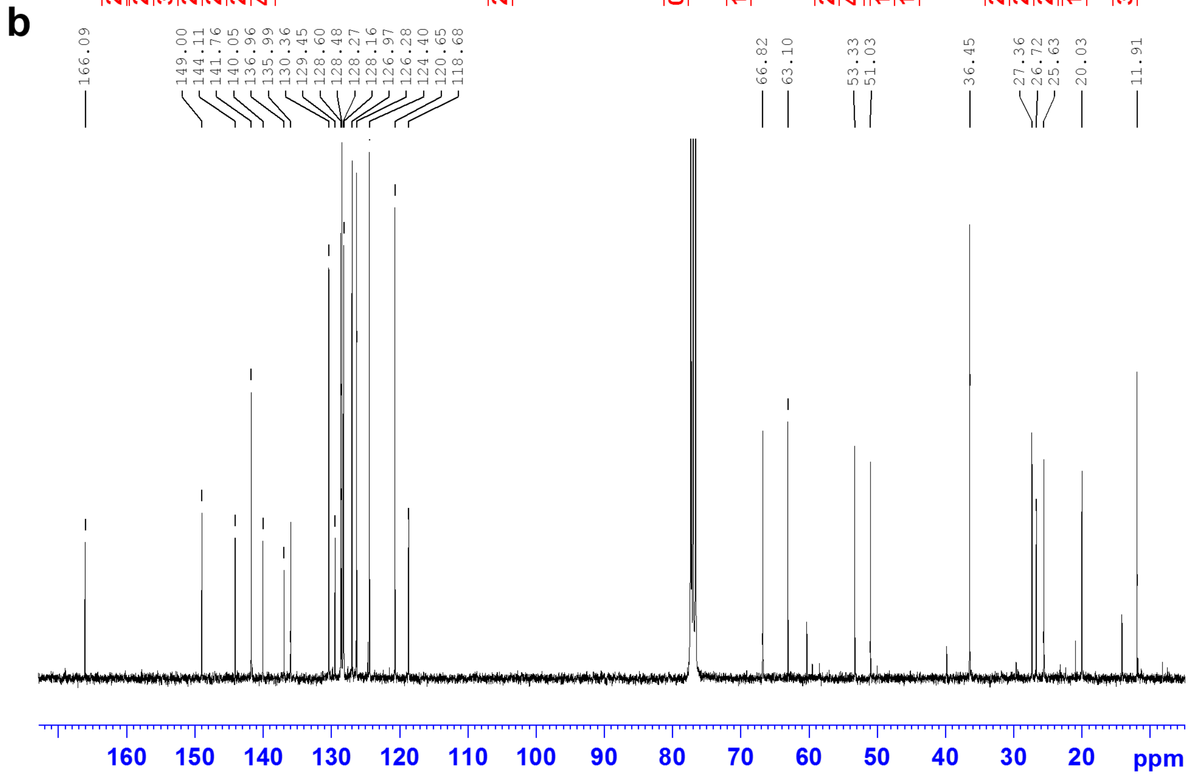


**Supplementary Figure 30: NMR spectra of compound 19.** (**a**) $^{1}H$ ($CDCl_3$, 360 MHz) and (**b**) $^{13}C$ ($CDCl_3$, 90 MHz).

a

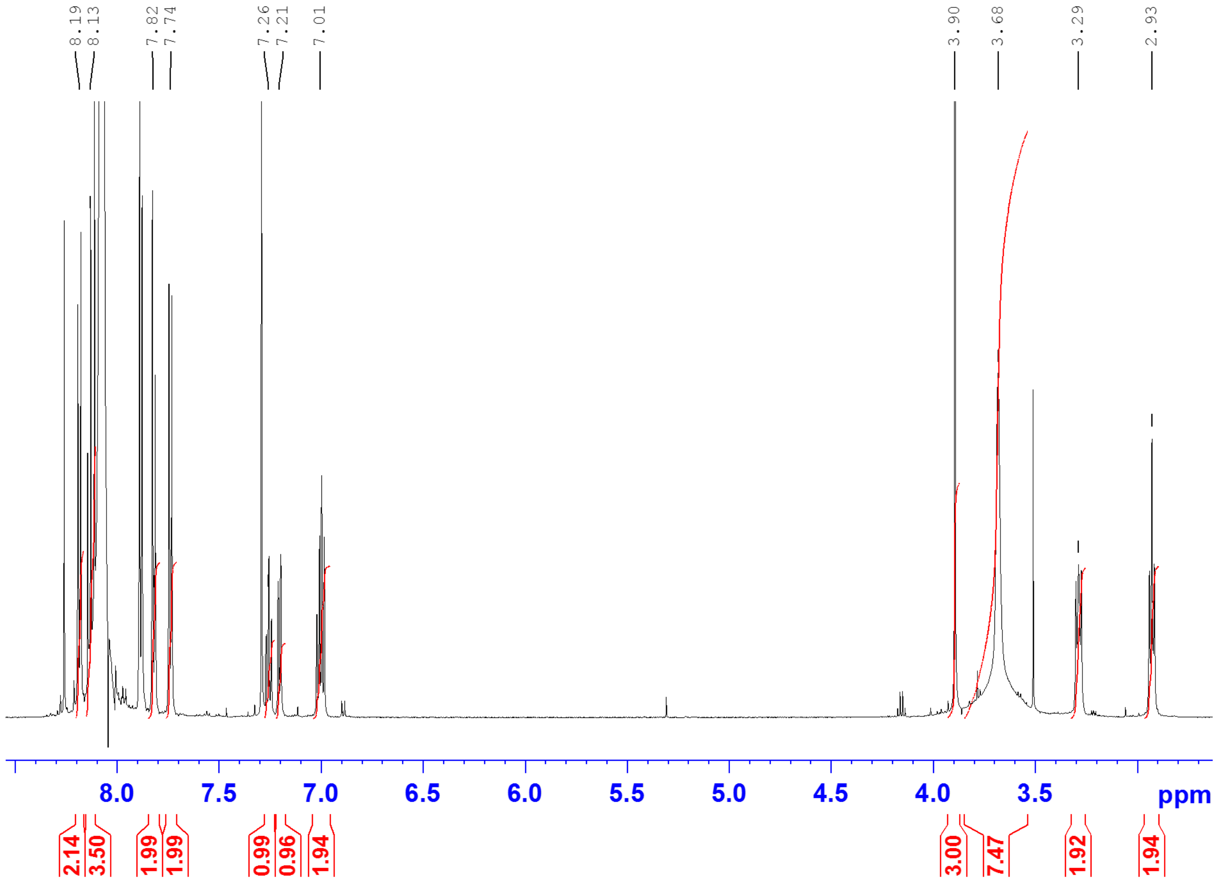


b

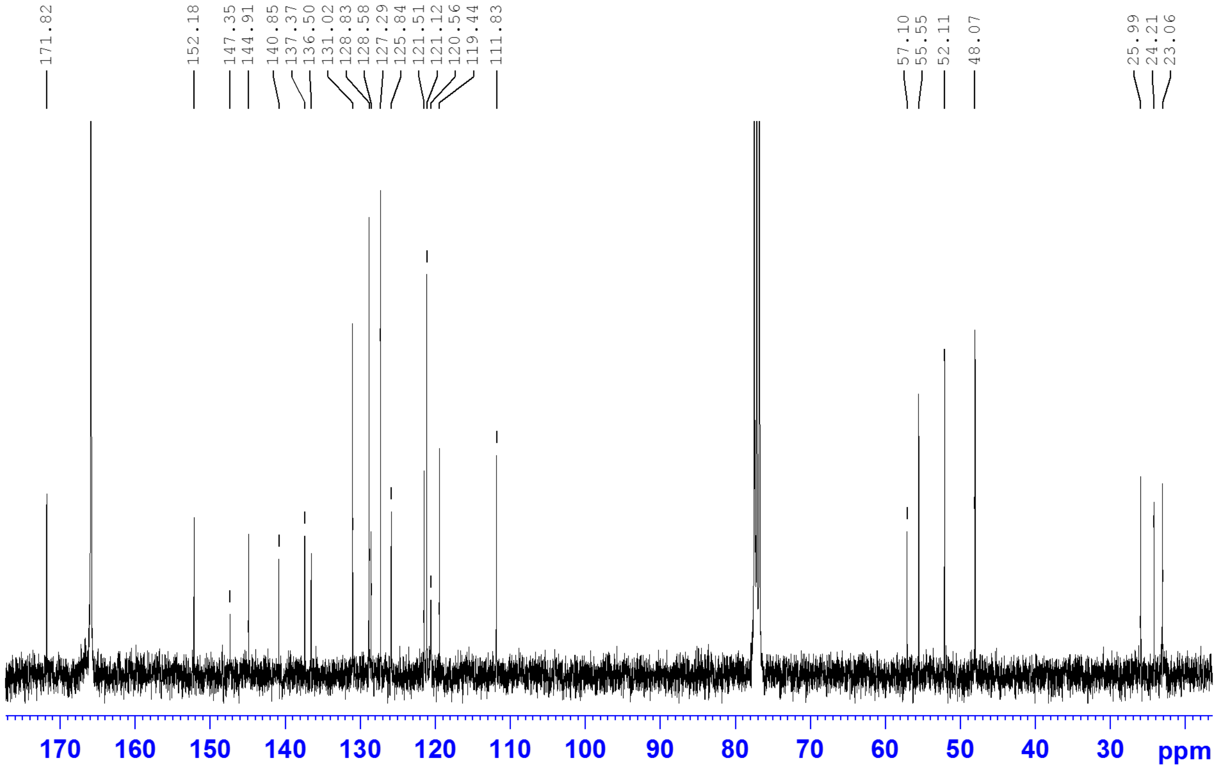


**Supplementary Figure 31: NMR spectra of compound 20.** (**a**) $^{1}H$ ($CDCl_3$ + 2 drops HCOOH, 600 MHz) and (**b**) $^{13}C$ ($CDCl_3$ + 2 drops HCOOH, 90 MHz).

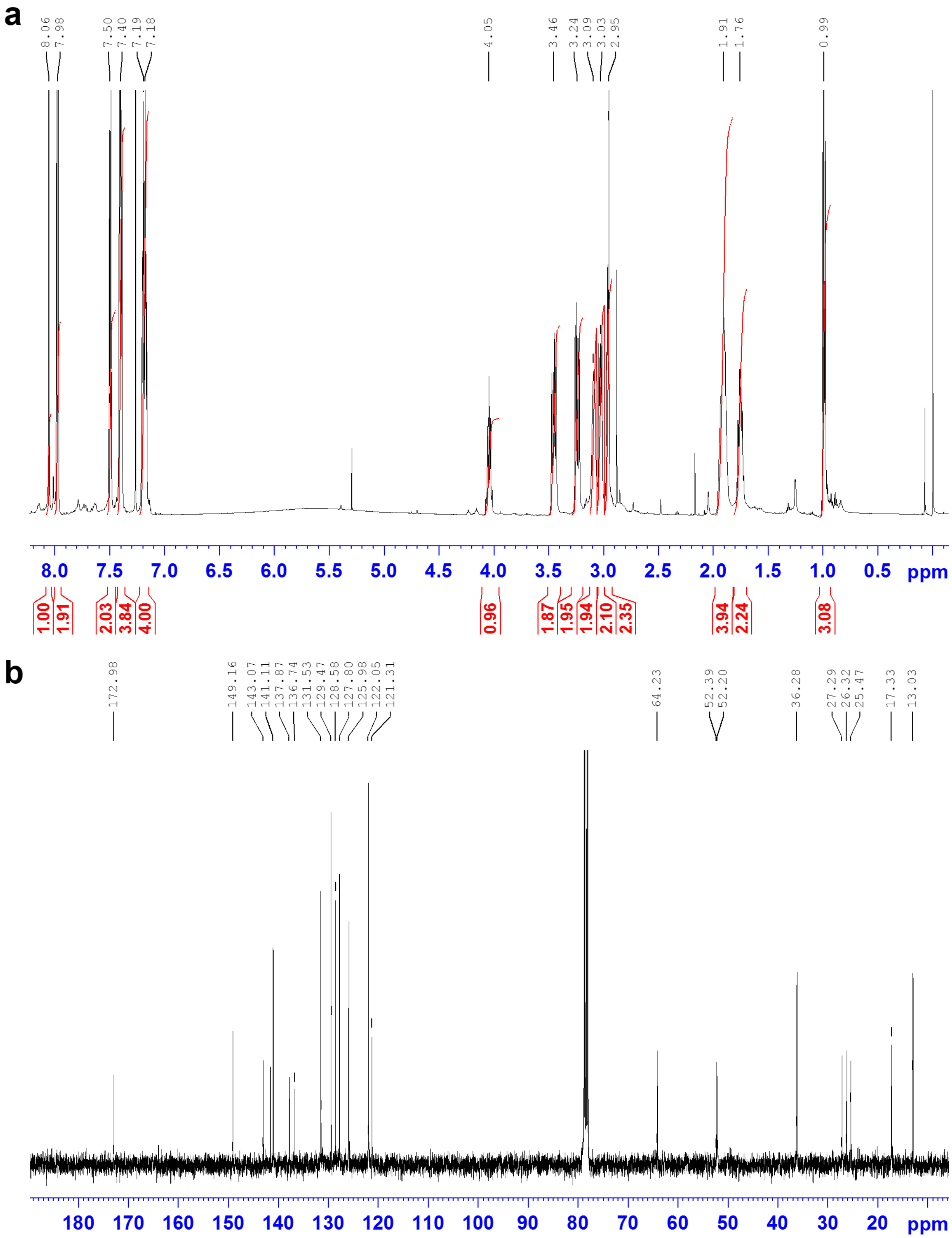


**Supplementary Figure 32: NMR spectra of compound 21.** (**a**) $^{1}H$ ($CDCl_3$, 600 MHz) and (**b**) $^{13}C$ ($CDCl_3$ + 2 drops HCOOH, 90 MHz).

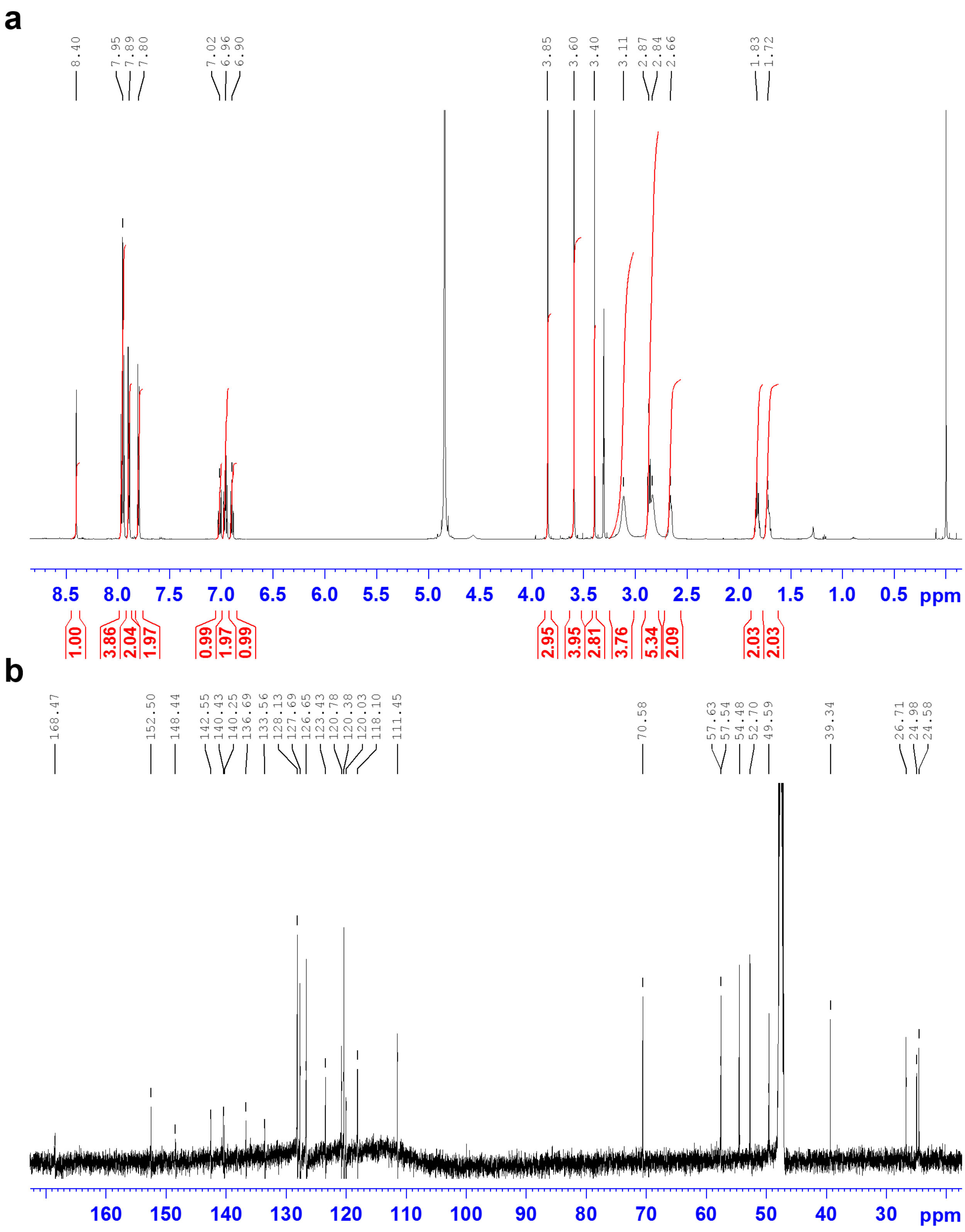


**Supplementary Figure 33: NMR spectra of compound 2g.** (**a**) $^{1}H$ ($CD_3OD$, 600 MHz) and (**b**) $^{13}C$ ($CD_3OD$, 150 MHz).

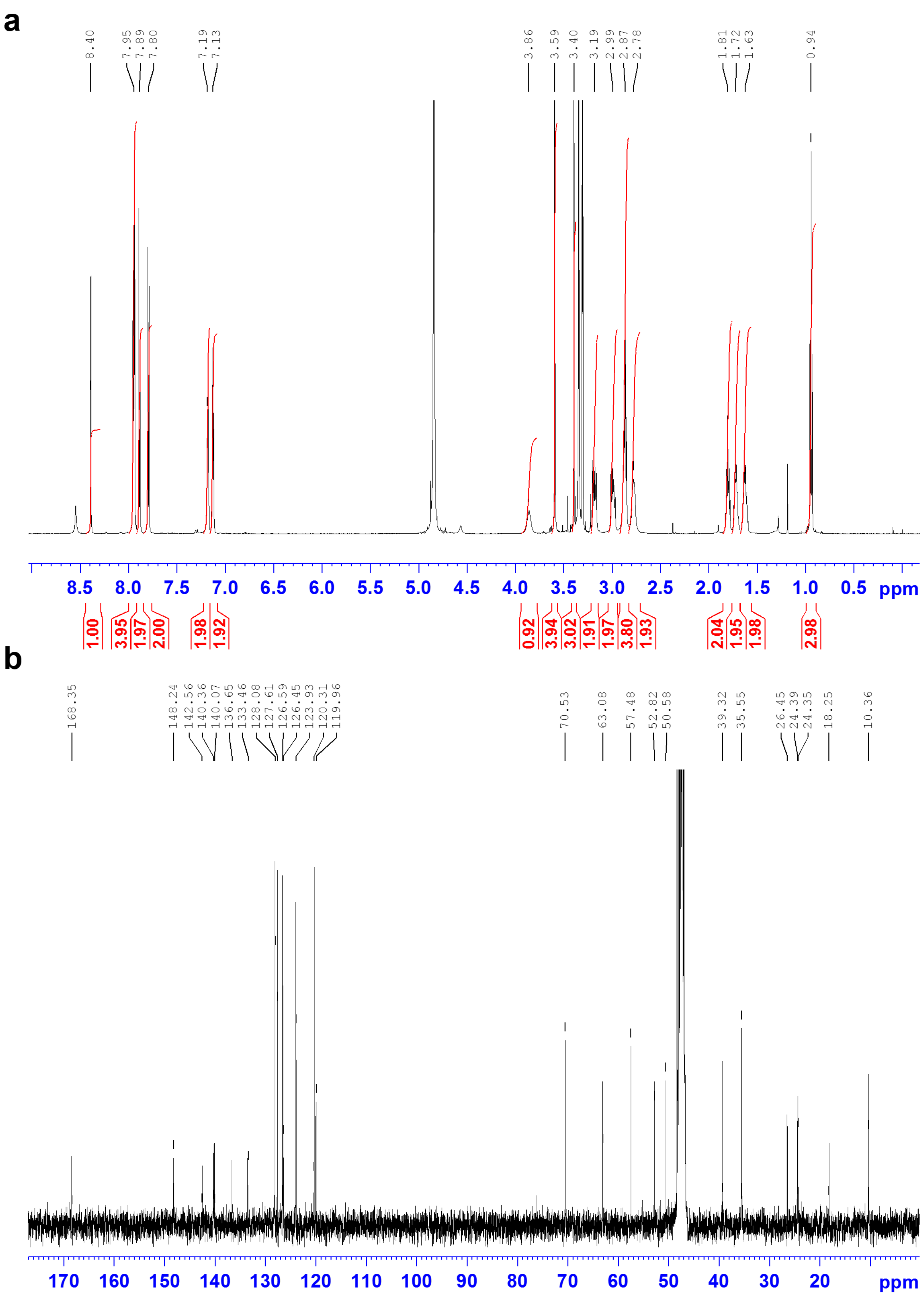


**Supplementary Figure 34: NMR spectra of compound 3g.** (**a**) $^{1}H$ ($CD_3OD$, 600 MHz) and (**b**) $^{13}C$ ($CD_3OD$, 150 MHz).

**Supplementary Table 1. Homo- and heterodimer interactions of $D_2R$**

| DIMER | PROPOSED INTERFACE | METHOD | YEAR | REFERENCE |
|---|---|---|---|---|
| $D_2R$-$D_2R$ | TM4 | Cross-linking experiments | 2003 | [3] |
| $D_2R$-$D_2R$ | TM4 | Co-Immunoprecipitation with receptor fragments | 2003 | [4] |
| $D_2R$-$D_2R$ | TM4 | Cys-mutations & cross-linking experiments | 2005 | [5] |
| $D_2R$-$D_2R$ | TM1/H8, TM4 | Cys-mutations & cross-linking experiments | 2008 | [6] |
| $D_2R$-$D_2R$ | TM1/H8 | Receptor bridging ligands | 2011 | [7] |
| $A_{2A}R$-$D_2R$ | $D_2$-ICL3, $A_{2A}$-CTer | MS & pull-down experiments | 2004 | [8] |
| $A_{2A}R$-$D_2R$ | $A_{2A}$-CTer | FRET & BRET | 2010 | [9] |
| 5-$HT_{2A}R$-$D_2R$ | $D_2$-ICL3, 5$HT_{2A}$-CTer | FRET | 2010 | [10] |
| $A_{2A}R$-$D_2R$ | $D_2$-TM4-5-ICL3, $A_{2A}$-CTer | BRET & treatment with TM-fragments | 2010 | [11] |
| $D_1R$-$D_2R$ | $D_2$-ICL3, $D_1$-CTer | Pull down experiments with GST fusion protein | 2010 | [12] |
| $D_1R$-$D_2R$ | $D_2$-ICL3, $D_1$-CTer | Fluorescence microscopy, Co-translocation | 2012 | [13] |
| $D_5R$-$D_2R$ | $D_2$-ICL3, $D_5$-CTer | Fluorescence microscopy, Co-translocation | 2013 | [14] |
| $D_1R$-$D_2R$ | $D_2$-ICL3, $D_1$-CTer | FRET, treatment with $D_1$ C-terminus fragment | 2014 | [15] |

## Supplementary Table 2. Published crystal structures of GPCR dimers

| RECEPTOR | PDB-ID | INTERFACE | HETERODIMER MODEL* | | | |
|---|---|---|---|---|---|---|
| | | | Protomer Clashes† | High Distance‡ | Low Parallelism§ | |
| $A_{2A}R$ | 4EIY | TM4, 5 , ICL2 | | - - | | Primary lipid mediated contact |
| $β_1$-AR | 4GPO | TM1, 2, H8 | - | | | Interfaces verified by cross-linking-studies |
| | | TM4, 5, ICL2 | | - | | |
| $β_2$-AR | 2RH1 | TM1, 2, H8 | | - - | - | Primary lipid mediated contact |
| CXCR4 | 3OE0 | TM3, 5, ICL2 | | - | - | |
| | 3OE9 | TM5, 6 | | - | - | |
| | 4RWS | TM5, 6 | | - | - | |
| $H_1R$ | 3RZE | TM3, 4 | | | - - | |
| κOR | 4DJH | TM1, 2, H8 | - | | | |
| $M_3R$ | 4U15 | TM1, 2, H8 | | | | Result of T4L modifications |
| μOR | 4DKL | TM1, 2, H8 | - | | - | |
| | | TM5, 6 | - - - | | - | |
| Opsin | 3CAP | TM1, 2, 7, H8 | | | - - | |
| | 3DQB | TM1, H8 | - - | | - - | |
| $P2Y_{12}R$ | 4NTJ | TM5, 6 | | - - | | Primary lipid mediated contact |
| Rhodopsin | 4BEZ | TM1, 2, H8 | - - | | - - | |
| | 2Z73 | TM4, 5, ICL2 | | - - | | |
| SMOR | 4JKV | TM4, 5, ICL2 | - - - | | | |

A summary of crystal structures of GPCRs that have been solved as dimers. *Heterodimer models were built as described in the methods section. Instead of the $β_1$-AR dimer, the crystal structure in question was used as template for structural alignment. Constructed dimer models were assessed after following three criteria for its suitability to function as template for modelling of a $D_2R$ and $NTS_1R$ dimer. †Protomer clashes were rated by excess of clashes between protomer 1 and protomer 2. Clash of side-chain atoms (-), clash of side-chain with backbone-atoms (- -), clash of backbone atoms (- - -). ‡For the distance rating between both protomers we looked at interactions along the dimer interface, interactions along less than half of interface (-), interaction mainly mediated by membrane lipids or cholesterol (- -). §The parallelism of both protomers was used to rate the possibility of the dimer model to exist in a membrane environment in this orientation. Therefore we looked at the parallelism of both helix bundles as well as the individual receptor position relative to a potential membrane by measuring the angles between both protomers (> 30° (-)) and between helices eight and a theoretical membrane plane (25° < x ≤ 35° (-), > 35° (- -)).

## Supplementary Table 3: Binding affinities of ligands 1a-d,2a-g and 3a-g at the human receptors $D_{2L}R$, $D_{2S}R$, $D_3R$, $D_{4.4}R$, $NTS_1R$ and $NTS_2R$

| comp. | $K_i$ values [nM][a] | | | | | |
|---|---|---|---|---|---|---|
| | $hD_{2L}R$ | $hD_{2S}R$ | $hD_3R$ | $hD_{4.4}R$ | $hNTS_1R$ | $hNTS_2R$ |
| | [3H]spiperone | | | | [3H]NT | [3H]NT(8-13) |
| **1a** | 1.7 ± 0.6 (*4*) | 1.5 ± 1.4 (*4*) | 1.0 ± 0.4 (*4*) | 470 ± 420 (*4*) | 0.23 ± 0.06 (*2*) | 16 ± 13 (*4*) |
| **1b** | 15 ± 5 (*3*) | 7.6 ± 4.6 (*4*) | 1.7 ± 0.8 (*4*) | 850 ± 750 (*4*) | 0.36 ± 0.05 (*2*) | 22 ± 16 (*4*) |
| **1c** | 3.5 ± 2.3 (*5*) | 2.1 ± 2.0 (*5*) | 1.4 ± 1.0 (*3*) | 420 ± 100 (*2*) | 0.20 ± 0.01 (*2*) | 4.2 ± 2.2 (*4*) |
| **1d** | 9.9 ± 4.5 (*2*) | 12 ± 10 (*2*) | 1.6 ± 0.4 (*2*) | 440 ± 190 (*2*) | 0.48 ± 0.27[b] (*2*) | 1.7 ± 0.20 (*2*) |
| **2a** | 52 ± 8 (*2*) | 20 ± 6 (*2*) | 2.7 ± 0.1 (*2*) | 62 ± 4 (*2*) | 2.6 ± 1.7 (*2*) | 4.7 ± 1.9 (*2*) |
| **2b** | 210 ± 80 (*2*) | 73 ± 7 (*2*) | 5.5 ± 0.9 (*2*) | 180 ± 20 (*2*) | 3.6 ± 0.8 (*2*) | 9.3 ± 6.7 (*2*) |
| **2c** | 98 ± 46 (*2*) | 74 ± 9 (*2*) | 6.6 ± 0.5 (*2*) | 430 ± 220 (*2*) | 0.24 ± 0.02 (*2*) | 5.6 ± 2.8 (*2*) |
| **2d** | 500 ± 40 (*2*) | 190 ± 10 (*2*) | 18 ± 6 (*2*) | 370 ± 50 (*2*) | 0.83 ± 0.09 (*2*) | 7.1 ± 4 (*2*) |
| **2e** | 56 ± 13 (*2*) | 23 ± 2 (*2*) | 5.8 ± 1.3 (*2*) | 140 ± 40 (*2*) | 18000 ± 8000 (*2*) | 20 ± 0 (*2*) |
| **2f** | 160 ± 100 (*2*) | 90 ± 57 (*2*) | 13 ± 12 (*2*) | 260 ± 170 (*2*) | 14000 ± 2000 (*2*) | 24 ± 4 (*2*) |
| **2g** | 37 ± 5 (*2*) | 22 ± 5 (*2*) | 3.4 ± 0.2 (*2*) | 33 ± 1 (*2*) | 5300 ± 1300 (*2*) | 4200 ± 900 (*2*) |
| **3a** | 25 ± 3 (*2*) | 3.3 ± 0.0 (*2*) | 0.43 ± 0.07 (*2*) | 25 ± 10 (*2*) | 1.5 ± 0.1 (*2*) | 9.7 ± 0.3 (*2*) |
| **3b** | 130 ± 50 (*2*) | 24 ± 1 (*2*) | 1.0 ± 0.1 (*2*) | 63 ± 14 (*2*) | 0.86 ± 0.79 (*4*) | 13 ± 9 (*4*) |
| **3c** | 19 ± 8 (*2*) | 6.6 ± 6.2 (*2*) | 0.45 ± 0.12 (*2*) | 80 ± 26 (*2*) | 0.34 ± 0.29 (*4*) | 6.8 ± 4.2 (*4*) |
| **3d** | 130 ± 20 (*2*) | 35 ± 18 (*2*) | 0.64 ± 0.06 (*2*) | 180 ± 40 (*2*) | 0.57 ± 0.12 (*2*) | 2.0 ± 1.0 (*2*) |
| **3e** | 19 ± 1 (*2*) | 6.4 ± 0.4 (*2*) | 0.62 ± 0.03 (*2*) | 40 ±6 (*2*) | 1300 ± 500 (*4*) | 25 ± 12 (*4*) |
| **3f** | 72 ± 29 (*2*) | 24 ± 4 (*2*) | 1.1 ± 0.1 (*2*) | 71 ± 4 (*2*) | 28000 ±3000 (*2*) | 20 ± 0 (*2*) |
| **3g** | 7.6 ± 1.7 (*2*) | 3.3 ± 0.8 (*2*) | 0.53 ± 0.25 (*2*) | 13 ± 1 (*2*) | 180 ± 40 (*2*) | 300 ± 250 (*2*) |

Receptor binding affinities were determined by radioligand displacement studies performed with the indicated radioligands as described in the supplementary methods section. [a] Mean values ± standard deviation (s.d.) are derived from (*n*) individual experiments, each performed in triplicate. [b] radioligand [3H]NT(8-13).

**Supplementary Table 4: cAMP accumulation assay (CAMYEL-bisoensor) in $D_2R$, $NTS_1R$ and $D_2R/NTS_1R$ expressing cells**

| comp. | $D_{2S}R$ | | $NTS_1R$ | | $D_{2S}R/NTS_1R$ | |
|---|---|---|---|---|---|---|
| | $EC_{50}$ [nM] | Effect [%][a] | $EC_{50}$ [nM] | Effect [%][a] | $EC_{50}$ [nM] | Effect [%][a] |
| quinpirole | 2.3 ± 0.6 (*11*) | -64 ± 1 | n.d. (*11*) | n.d. | 3.0 ± 0.4 (*11*) | -57 ± 1 |
| NT(8-13) | n.d. (*11*) | n.d. | 2.6 ± 0.2 (*11*) | 73 ± 1 | 5.0 ± 0.5 (*11*) | 24 ± 1 |
| **2b** | n.d. (*5*) | n.d. | 20.7 ± 3.2 (*5*) | 74 ± 2 | 39.3 ± 5.6 (*5*) | 31 ± 3 |
| **2c** | n.d. (*3*) | n.d. | 32.5 ± 3.5 (*3*) | 71 ± 2 | 45.8 ± 2.3 (*3*) | 36 ± 2 |
| **2g** | n.d. (*3*) | n.d. | n.d. (*3*) | n.d. | n.d. (*3*) | n.d. |
| **3b** | 5.0 ± 1.4 (*4*) | -71 ± 2 | 30.6 ± 6.1 (*4*) | 70 ± 3 | 70.0 ± 5.1 (*4*) | 23 ± 2 |
| **3c** | 10.3 ± 1.3 (*3*) | -73 ± 2 | 29.7 ± 1.5 (*3*) | 71 ± 2 | 46.9 ± 2.2 (*3*) | 37 ± 3 |
| **3g** | 2.8 ± 0.4 (*5*) | -65 ± 1 | n.d. (*5*) | n.d. | 3.5 ± 1.4 (*5*) | -63 ± 2 |

[a] Effect = $E_{max}$ relative to the effect of 10 μM forskolin (=100 %) and the unstimulated effect by buffer (= 0 %) subtracted by the effect of 10 μM forskolin. Negative values reflect the activation of a $G_{i/o}$ system leading to the reduction of cAMP concentration. Positive numbers reflect the activation of a $G_s$ system leading to an additional increase of cAMP. Data represent mean ± s.e.m. derived from (*n*) individual experiments each performed in triplicate. n.d.: no effect determined.

**Supplementary Note 1: Selection of the dimeric $\beta_1$-AR crystal structure as a scaffold for the generation of the $D_2R/NTS_1R$ heterodimer models**

Aiming to build a reliable dopamine $D_2$ receptor/neurotensin receptor 1 ($D_2R/NTS_1R$) heterodimer model, we planned to arrange both receptors in a way that is compatible with experimental studies on direct interactions of the individual receptors. As, to our knowledge, experimentally derived information providing a molecular basis for the architecture of a $D_2R/NTS_1R$ heterodimer has not been described, we focused on studies of $D_2Rs$ in homodimers and in heterodimers with other GPCRs (Supplementary Table 1). For $NTS_1R$, no studies on specific interactions of homo- or heterodimers were reported, yet. Guo et al. showed $D_2Rs$ can use two distinct homodimer interfaces[6]: Thus, a dimer interface may be formed between transmembrane helix 1 (TM1) and helix 8 (H8) of each protomer. Alternatively, dimerization involving TM4 has been described. Employing bivalent receptor-bridging ligands, our lab suggested protomer interaction involving TM1 and H8[7]. Here, the proposed binding modes of the pharmacophores used for bivalent ligands, in combination with a relatively short linker suggested that simultaneous binding to the two receptors may only be possible in a $D_2R$ dimer, in which TM1 and H8 form the interface. For $D_2R$/adenosine $A_{2A}R$ heterodimers, TM4 and TM5 of the $D_2R$ were suggested to be involved in the formation of heterodimers[11]. Furthermore, different groups reported that the key interaction between $D_2R$ and other GPCRs features a salt bridge, which may be formed between two adjacent arginine residues in the intracellular loop (IL) 3 (Arg274 and Arg275) of the $D_2R$ and two adjacent aspartate or glutamate residues within the interacting GPCR (5-$HT_{2A}R$[10], $A_{2A}R$[8], $D_1R$[15] and $D_5R$[14]). Because the amino acid sequence of the $NTS_1R$ does not contain the acidic counterpart to the two adjacent arginine residues of $D_2R$, we only considered GPCR crystal structures as a template if the particular dimer involves either TM4 and TM5 or TM1 and helix 8.

To date, 16 crystal structures of 12 different GPCRs showed the formation of homodimers with 18 more or less different receptor orientations (Supplementary Table 2). We created models of $D_2R/NTS_1R$ heterodimers by structurally aligning the $NTS_1R$ crystal structure (PDB-ID 4BUO)[16] and our recently described $D_2R$ homology model[1] with the crystal structures of all 18 GPCR dimers. Models were not considered further if they showed substantial clashes between the two receptors, as well as models revealing a high distance between the protomers or showing a low parallelism of the two protomers. Taking into account this evaluation and showing relatively high sequence similarity with $D_2R$, a crystal structure of the $\beta_1$ adrenergic receptor (PDB-ID 4GPO)[17] was used as a template, featuring the above described interface involving TM1 and helix 8. This architecture can also be considered biologically relevant because the interface was verified by *in vitro* cross-linking studies. The crystal displayed a second crystallographic asymmetric unit featuring a dimer with an interface between TM4 and 5. The respective dimer, however, did not allow formation of a stable GPCR- G protein complex, when the $\beta_2$-AR active-state crystal structure was used as a template[17]. Taking further into account our previous studies on $D_2R$ homodimers[7], in which the interface was suggested to be formed via TM1 and helix 8,

we selected the heterodimer that showed an interface between TM1 and H8 of each protomer. Thus, superimposing our $D_2R$ homology model and the $NTS_1R$ crystal structure with the $\beta_1$-AR dimer template yielded a $D_2R/NTS_1R$ heterodimer model (Supplementary Fig. 1) that we used for the structure guided-design of heterobivalent ligands.

**Supplementary Note 2: Structure-guided design**

Our heterobivalent ligands comprise a $D_2R$ pharmacophore (eticlopride, a phenylpiperazine or an aminoindane-type scaffold) coupled to an affinity-generating lipophilic appendage (consisting of a biphenyltriazole moiety)[18, 19]. The appendage adopting an extension of the binding pocket directs to the extracellular region. The appendage is connected to the $NTS_1R$ agonist NT(8-13) via a flexible spacer of varying length to enable bridging of the binding pockets of $D_2R$ and $NTS_1R$. As a spacer element, we used $\omega$-amino-acid functionalized PEG-units. Possible attachment points of eticlopride and NT(8-13) were identified using the crystal structures of $D_3R$ (which shows high homology to $D_2R$) and $NTS_1R$, bound to the before mentioned pharmacophores, displaying that the 4'-position of eticlopride and the N-terminus of NT(8-13) are accessible from the extracellular side. In an effort to determine a reasonable linker length, we first manually docked eticlopride into our "X-ray based" heterodimer model. The model revealed a distance of 42 Å between the attachment points of eticlopride and NT(8-13). Because the length of the lipophilic appendage is approximately 20 Å, this would allow bridging of both pharmacophores with one $\omega$-amino-acid functionalized PEG-unit (22 atoms) featuring a length of 26 Å in a fully extended conformation. However, docking of eticlopride with the lipophilic appendage into the heterodimer model revealed two reasons why a significantly longer spacer length was required. First, the binding pocket of $D_2R$ restricts the appendage in a position not facing strait towards $NTS_1R$ (Supplementary Fig. 2a). Second, the way is partially blocked by the extracellular loop 1 of $D_2R$ and the N-terminus of $NTS_1R$. The measurement of the shortest possible connection resulted in a total distance of about 55 Å. We concluded that at least two spacer units should be necessary to enable a bivalent binding-mode, while a structure with only one spacer unit should lack the ability to bridge both binding sites (Supplementary Fig. 2b). To check for an optimum spacer length, we considered compounds **1b**, **1c** and **1d** bearing two, three and four units of the functionalized PEG-spacer, respectively. **1a** with one linker unit should be used as a control compound.

Attachment points for the phenylpiperazine and aminoindane-type scaffolds were identified based on docking studies (Supplementary Fig. 3). In both cases the basic nitrogen was selected to link the pharmacophore with the lipophilic appendage. In analogy to the structure-based strategy above, we designed compounds **2a-d** (phenylpiperazine-type) and **3a-d** (aminoindane-type) with one, two, three and four PEG-units, respectively.

**Supplementary Note 3: Molecular Dynamics simulations of a 1b-, 2b- and 3b-bound $D_2R/NTS_1R$ dimer model**

We used the crystal structure of the rat $NTS_1R$ in complex with NT(8-13) (PDB ID: 4BUO)[16] as a template to create a homology model of the human $NTS_1R$ as described previously[20]. One final $NTS_1R$ model was selected manually. The $D_2R/NTS_1R$ dimer model was created as described within the dimer modelling section. Due to the technical difficulties of docking bivalent ligands into a heterodimer model with two separate binding pockets we applied a modelling approach with multiple stages. First, the bivalent ligands were divided into reasonable parts (at the four amide bonds). This led to four parts, the $D_2R$ pharmacophore with attachment, the $NTS_1R$ pharmacophore and two ω-amino-acid functionalized PEG-units. Subsequently, the $D_2R$ pharmacophores were docked into the $D_2R$ binding site as described in the methods section. For the $NTS_1R$ pharmacophore, we superimposed the dimer model with the $NTS_1R$ crystal structure and afterwards transferred the coordinates of NT(8-13) to the dimer model. The initial conformation of the linker moiety of **1b**, **2b** and **3b** (connecting both pharmacophores) was modeled in a way that avoids steric clashes of the linker and the receptor dimer. The final ligand-receptor complexes were submitted to energy minimization using the SANDER module of AMBER10 as described[1]. The all-atom force field ff99SB[21] was used for standard amino-acid residues. For the bivalent ligands **2b** and **3b**, we used ff99SB for the NT(8-13)-part of the ligand and the general AMBER force field (GAFF)[22] for the linker and $D_2R$ pharmacophores. Charges for the non-peptidic parts were calculated using Gaussian09[23] at HF/6-31G(d) level and a manually conducted RESP[24] procedure, in which we adapted the charges of the linker and $D_2R$ pharmacophores while keeping the charges of the NT(8-13) atoms at their values taken from the ff99SB force field. A formal charge of +2 was defined for all ligands.

Eticlopride carries an aromatic chlorine atom, which shows an anisotropic charge distribution resulting in a σ-hole, a small positive area along the C-Cl axis and opposite of C[25]. Similar to hydrogens in a hydrogen bond, this area can interact with negative interaction sites[26]. As shown by Rendine *et al.*[27], it is advantageous to consider halogen bonding for Molecular Dynamics simulations with halogenated molecules to obtain correct binding positions. To include the σ-hole in our MD calculations on compound **1b**, we added a single point charge (a pseudo atom) along the C-Cl axis of the eticlopride pharmacophore and included the pseudo atom in the RESP fit procedure, in analogy to a method previously suggested by several groups[27-29]. Subsequently we applied the same procedures as described for compounds **2b** and **3b**.

For the membrane simulations, the AMBER topology and coordinate files for the minimized complex were converted into GROMACS[30, 31] input files. The ligand−bound dimer complex was inserted into a pre-equilibrated membrane of dioleoylphosphatidylcholine (DOPC) lipids by means of the GROMACS tool g_membed[32]. A pre-equilibrated system bearing a hydrated membrane with 72 DOPC lipids[33] was used as a starting point, which had to be enlarged in the x, y and z dimensions as described earlier[34] to

fully surround the dimer model. The charges of the simulation systems were neutralized by adding 19 chloride atoms each. In total, the systems consisted of 274,657 atoms (600 amino acids, 572 DOPC lipids, 19 chloride atoms and 61,910 water molecules) for the system with compound **1b**, 268,916 atoms (600 amino acids, 534 DOPC lipids, 19 chloride atoms and 61,751 water molecules) for the system with compound **2b** and 273,356 atoms (600 amino acids, 570 DOPC lipids, 19 chloride atoms and 61,575 water molecules) for the system with compound **3b**. Within the membrane simulations, GAFF was used for DOPC molecules and the force field ff99SB for the protein residues. Parameters for compounds **1b**, **2b** and **3b** were used as described above. The SPC/E water model[35] was applied. After insertion into the membrane, the simulation systems were submitted to an equilibration run of 10 ns, in which we applied restraints of 1.0 kcal $mol^{-1}$ $Å^{-2}$ on all atoms of the ligand-dimer complex for the first 1 ns, on all atoms of the receptors for the next 2 ns, and on the main chain atoms of the receptors for further 2 ns. Subsequently, productive molecular dynamics simulation runs of 400 ns each were performed, using the GROMACS 4.5.2 simulation package as described earlier[34]. The analysis of the trajectories was performed with PTRAJ module of AMBER10. The figures were prepared using UCSF Chimera package 1.10.[36]

## Supplementary Methods

### Determination of binding affinities for a panel of $D_2$-like receptors and the receptor subtypes $NTS_1R$ and $NTS_2R$

Receptor binding studies were carried out as described previously[37]. In brief, competition binding experiments with the human $D_{2L}R$, $D_{2S}R$[38], $D_3R$[39], and $D_{4.4}R$[40] receptors were perfomed using preparations of membranes from CHO cells stably expressing the corresponding receptor together with [$^3$H]spiperone (specific activity = 81 Ci mmol$^{-1}$, PerkinElmer, Rodgau, Germany) at a final concentration of 0.10-0.50 nM. The assays were carried out at a protein concentration of 1-8 μg per assay tube, $K_D$ values of 0.052-0.12 nM, 0.032-0.092 nM, 0.13-0.18 nM, and 0.17-0.35 nM and corresponding $B_{max}$ values of 500-1500 fmol mg$^{-1}$, 1500-8500 fmol mg$^{-1}$, 2200-5000 fmol mg$^{-1}$, and 1000-4500 fmol mg$^{-1}$ for the $D_{2L}R$, $D_{2S}R$, $D_3R$, and $D_{4.4}R$ receptors, respectively. Homogenates from CHO cells stably expressing the human $NTS_1R$ (cDNA purchased from Missouri S&T cDNA Resource Center (UMR), Rolla, MO) at a $B_{max}$ of 2500-6200 fmol mg$^{-1}$ and with $K_D$ values of 0.37-0.69 nM were incubated at 2 μg per assay tube with the radioligand [$^3$H]neurotensin (NT, final concentratrion 0.50 nM, specific activity 101 Ci mmol$^{-1}$; PerkinElmer, Rodgau, Germany). Human $NTS_2R$ binding was achieved using homogenates from HEK 293T cells, which were transiently transfected with pcDNA3.1 (UMR) by the calcium phosphate method[41]. Membranes were incubated at a final concentration of 4 μg per well with $B_{max}$ values of 350-450 fmol mg$^{-1}$ and $K_D$ values of 0.80-1.6 nM using the radioligand [$^3$H]NT(8-13) (final concentratrion 0.50 nM, specific activity 136 Ci mmol$^{-1}$; custom synthesis of [leucine-$^3$H]NT(8-13) by GE Healthcare, Freiburg, Germany). Unspecific binding was determined in the presence of haloperidol (10 μM for $D_{2L}R$-$D_{4.4}R$) or NT(8-13) (10 μM for $NTS_1R$ and $NTS_2R$). Protein concentration was established by the method of Lowry using bovine serum albumin as standard[42].

### General materials and methods for organic synthesis

Reagents and dry solvents were obtained from commercial sources and were used as received. Reactions were conducted under dry $N_2$. Evaporations of product solutions were done *in vacuo* with a rotary evaporator. Column chromatography was performed using 60 μm silica gel. For thin layer chromatography (TLC) silica gel 60 μm plates were used (ultraviolet (UV), $I_2$, $KMnO_4$ or ninhydrin detection). Preparative RP-HPLC was performed employing Agilent 1100 preparative series, column Zorbax Eclipse XDB-C8, 21.2 mm × 150 mm, 5 μm particles [C8], employing solvent systems, linear gradient and flow rate [FR] as specified below or VP 250/32 NUCLEODUR C18 HTec 5 μm particles [C18] employing solvent systems, linear gradient and flow rate [FR] as specified below. Melting temperatures are uncorrected. Infrared (IR) spectra were registered from a thin film on a NaCl crystal. Nuclear magnetic resonance (NMR) data were acquired with the help of a 360 MHz or a 600 MHz spectrometer in the indicated solvents; $^{13}$C NMR spectra were recorded at 90 MHz or 150 MHz in the indicated solvents. Chemical shifts are given in $\delta$ relative to tetramethylsilane (TMS) in parts per million

(ppm) relative to TMS = 0. Mass spectra were acquired using electronic ionization (EI), atmospheric pressure chemical ionization (APCI) or electron spray ionization (ESI) techniques. EI high resolution mass spectra were measured with a JOEL GCmate II spectrometer. ESI-ToF high mass accuracy and resolution experiments were performed on a Bruker maXis MS (Bruker Daltonics, Bremen, Germany) in the laboratory of the Chair of Bioinorganic Chemistry (Prof. Dr. Ivana Ivanović-Burmazović), FAU. High performance liquid chromatography (HPLC) analysis revealed a purity > 95 % for all SAR compounds. Purity was assessed by analytical HPLC (Agilent 1100 analytical series, equipped with QuatPump and VWD detector, column: Zorbax Eclipse XDB-C8 analytical column, 4.6 × 150 mm, 5 μm, flow rate: 0.5 mL $min^{-1}$); System 1 (S1): x-y % $CH_3OH$ in $H_2O$ + 0.1 % $HCO_2H$ (S1A: 4-15 % in 18 min, 15-95 % in 2 min, 95-95 % in 2 min, S1B: 10-90 % in 18 min, 90-95 % in 2 min, 95-95 % in 2 min), S1C: 10-100 % in 15 min, 100-100 % in 6 min), System 2 (S2): x-y % $CH_3CN$ in $H_2O$ + 0.1 % $HCO_2H$ (S2A: 3-85 % in 26 min, 85-95 % in 2 min, 95-95 % in 2 min), S2B: 10-100 % in 15 min, 100-100 % in 6 min).

**Syntheses of bivalent ligands**

The synthesis was performed according to standard protocols as described below and was performed started from commercially available Fmoc-Leu-Wang resin. α-Amino acids as well as the polyethylenglycol (PEG)-Spacer were incorporated as their commercially available derivatives: Fmoc-Ile-OH, Fmoc-Tyr(O*ᵗ*Bu)-OH, Fmoc-Pro-OH, Fmoc-Arg(Pbf)-OH and Fmoc-NH-$PEG_6$-propionic acid (Fmoc-21-amino-4,7,10,13,16,19-hexaoxaheneicosanoic acid, Iris Biotech, Marktredwitz, Germany). Fmoc-N-[2-(4-tert-butoxyphenyl)ethyl]glycin (Fmoc-NhTyr(O$^t$Bu)-OH) was prepared according to a recently published protocol[43]. Elongation of the chain was done by repetitive cycles of Fmoc deprotection and subsequent coupling of the amino acid derivative, PEG-spacer and dopamine receptor ligands respectively with the help of microwave irradiation (Discover® microwave oven, CEM Corp.), performed in silanized glass tubes. Remark: the development of overpressure was avoided by using DMF as the solvent and intermittent cooling. Fmoc deprotection was performed by treatment of the resin (1×) with 20 % piperidine in DMF (microwave irradiation: 8× 5 s, 100 W), followed by washings with DMF (5×). Coupling steps were done following Method 1. After the last acylation the resin was rinsed with $CH_2Cl_2$ (5×) and dried *in vacuo*. The cleavage from the resin was performed using a mixture of trifluoroacetic acid (TFA) / phenol / $H_2O$ / triisopropylsilane (TIS) 88:5:5:2 for 3 h, followed by filtration of the resin. After evaporation of the solvent *in vacuo* and precipitation in *tert*-butylmethylether, the crude peptide derivatives were purified using preparative RP-HPLC: Agilent 1100 preparative series, column: VP 250/32 NUCLEODUR C18 HTec 5 μm particles employing solvent systems, linear gradient and flow rate [FR] as specified below.

After separation, the peptide derivatives were lyophilized and product purity and identity were assessed by analytical HPLC (Agilent 1100 analytical series, equipped with QuatPump and VWD detector, column: Zorbax Eclipse XDB-C8 analytical column, 4.6 × 150 mm, 5 μm, flow rate: 0.5 mL $min^{-1}$) coupled

to a Bruker Esquire 2000 mass detector equipped with an ESI-trap. System 1 (S1): x-y % $CH_3OH$ in $H_2O$ + 0.1 % $HCO_2H$ (S1A: 10-55 % in 18 min, 55-95 % in 2 min, 95-95 % in 2 min; S1B: 10-80 % in 18 min, 80-95 % in 2 min, 95-95 % in 2 min; S1C: 10-65 % in 18 min, 65-95 % in 2 min, 95-95 % in 2 min; S1D: 10-100 % in 15 min, 100-100 % in 6 min), System 2 (S2): x-y % $CH_3CN$ in $H_2O$ + 0.1 % $HCO_2H$ (S2A: 3-40 %, in 26 min, 40-95 % in 2 min, 95-95 % in 2 min; S2B: 5-50 %, in 18 min, 50-95 % in 2 min, 95-95 % in 2 min; S2C: 3-55 %, in 20 min, 55-95 % in 2 min, 95-95 % in 2 min, S2D: 10-100 % in 15 min, 100-100 % in 15 min). System 3 (S3): x-y % $CH_3OH$ in $H_2O$ + 0.1 % $F_3CCO_2H$ (S3A: 10-10 % in 3 min, 10-100 % in 60 min, 100-100 % in 3 min), System 4 (S4): x-y % $CH_3CN$ in $H_2O$ + 0.1 % $F_3CCO_2H$ (S4A: 10-10 % in 3 min, 10-100 % in 60 min, 100-100 % in 3 min).

**Method 1:** Peptide coupling was done employing 5 equiv. of each Fmoc-amino acid / PyBOP / DIPEA and 7.5 equiv 1-hydroxybenzotriazole (HOBt) dissolved in a minimum amount of DMF (irradiation: 20 × 10s, 50W). For introduction of the Fmoc-NH-$PEG_6$-propionic acid (2.5 equiv) also 5 equiv of PyBOP and DIPEA and 7.5 equiv HOBt were dissolved in a minimum amount of DMF. After microwave irradiation (20 × 10s, 50W) the mixture was shaken for additional 2 h. The dopamine receptor ligands **11**, **20** and **21** were coupled employing 2 equiv of **11**/ **20**/ **21**, 2 equiv of HATU and 4 equiv of DIPEA. After microwave irradiation (20 × 10s, 50W) the mixture was shaken for another 12 h. In between each irradiation step cooling of the reaction mixture to a temperature of -10 °C was achieved by sufficient agitation in an ethanol-ice bath.

**Experimental procedures for chemical synthesis**

(2*S*,4*R*)-1-Ethyl-4-(hex-5-yn-1-yloxy)-2-ethoxycarbonylpyrrolidine (**5**)

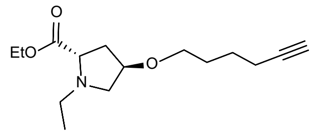

To a solution of (2*S*,4*R*)-1-ethyl-4-hydroxy-2-ethoxycarbonylpyrrolidine[44] (**4,** 0.30 g, 1.60 mmol) in DMF (10 mL) over 3 Å molecular sieves NaH (60 % suspension in mineral oil, 0.14 g, 3.58 mmol) was added at 0 °C. The resulting mixture was stirred at RT for 30 min. 6-Iodohex-1-yne (0.55 mL, 4.18 mmol) was added and the mixture was stirred at 50 °C for 18 h. After quenching with water at 0 °C, the mixture was partitioned between aqueous sat. $NH_4Cl$ solution and EtOAc. The aqueous phase was extracted with EtOAc (3x), and the combined organic phases were washed with brine (1x), dried ($MgSO_4$) and concentrated *in vacuo*. Flash column chromatography (hexane / EtOAc 1:2 + 0.2 % $NH_3$ 25 % aq.) afforded **5** as yellowish oil (0.14 g, 32 %): IR (film, NaCl): 3419, 3293, 2938, 2870, 2809, 2116, 1743, 1702, 1638, 1455, 1371, 1349, 1270, 1186, 1096, 1032, 637 $cm^{-1}$; $^1H$ NMR ($CDCl_3$, 600 MHz): $\delta$ 4.19 (q, 2H, $J$ = 7.2 Hz); 4.11-4.05 (m, 1H), 3.50–3.32 (m, 4H), 2.76 (dq, 1H, $J^1$ = 11.9 Hz, $J^2$ = 7.3 Hz), 2.47 (dq, 1H, $J^1$ = 11.9 Hz, $J^2$ = 7.2 Hz), 2.38 (dd, 1H, $J$ = 5.2 Hz), 2.25-2.19 (m, 3H), 1.95 (t, 1H, $J$ = 2.7 Hz),

1.82–1.75 (m, 1H), 1.70– 1.64 (m, 2), 1.63–1.56 (m, 2H), 1.27 (t, 3H, $J$ = 7.2 Hz), 1.09 (t, 3H, $J$ = 7.3 Hz); $^{13}$C NMR ($CDCl_3$, 150 MHz): δ 173.8, 84.3, 77.3, 68.7, 68.4, 64.8, 60.6, 58.8, 48.8, 36.5, 28.9, 25.4, 18.2, 14.2, 13.3; ESI-MS: (m/z): 268.5 $[M+H]^+$.

(2S,4R)-1-Ethyl-4-(hex-5-yn-1-yloxy)pyrrolidine-2-carboxamide (**6**)

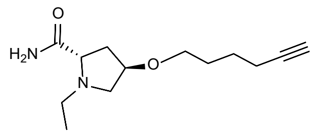

To a saturated solution of $NH_3$ in MeOH (20 mL) (2*S*,4*R*)-1-ethyl-4-(hex-5-yn-1-yloxy)-2-ethoxycarbonylpyrrolidine (**5**, 0.69 g, 2.60 mmol) and KI (0.043g, 0.26 mmol) were added. The reaction mixture was stirred at 40 °C for 14 days. Evaporation of volatiles and flash column chromatography (hexane / EtOAc 1:1 + 0.2 % $NH_3$ 25 % to EtOAc + 0.2 % $NH_3$ 25 % aq.) yielded **6** as a white solid (0.48 g, 78 %): mp 79 °C; $[\alpha]_D$ = -61,0° (c = 0.24 / MeOH, T = 27.5 °C); IR (film, NaCl): 3054, 2987, 2685, 2306, 1654, 1422, 1262, 869, 750 $cm^{-1}$; $^1$H NMR ($CDCl_3$, 600 MHz): δ 7.12 (s, 1H), 5,49 (s, 1H), 4.00–3.95 (m, 1H), 3.47–3.34 (m, 3H), 3.23 (dd, 1H, $J^1$ = $J^2$ = 8.1 Hz), 2.73 (dq, 1H, $J^1$ = 12.1 Hz, $J^2$ = 7.3 Hz), 2.51 (dq, 1H, $J^1$ = 12.5 Hz, $J^2$ = 7.0 Hz), 2.42 (dd, 1H, $J^1$ = 10.2 Hz, $J^2$ = 5.3 Hz), 2.24 (ddd, 1H, $J^1$ = 13.9 Hz, $J^2$ = 8.9 Hz, $J^3$ = 4.9 Hz), 2.21 (dt, 2H, $J^1$ = 12.5 Hz, $J^2$ = 2.5 Hz), 1.99 (ddd, 1H, $J^1$ = 13.5 Hz, $J^2$ = $J^3$ = 6.9 Hz), 1.95 (t, 1H, $J$ = 2.5 Hz), 1.70–1.63 (m, 2H), 1.63–1.55 (m, 2H), 1.08 (t, 3H, $J$ = 7.2 Hz); $^{13}$C NMR ($CDCl_3$, 150 MHz): δ 177.6, 84.2, 77.7, 68.7, 68.4, 66.4, 58.5, 50.2, 37.0, 28.9, 25.2, 18.2, 14.0; ESI-MS (*m/z*): 239.5 $[M+H]^+$.

(2'*S*,4'*R*)-3-Chloro-5-ethyl-*N*-((1-ethyl-4-(hex-5-yn-1-yloxy)pyrrolidin-2-yl)methyl)-6-hydroxy-2-methoxybenzamide (**8**)

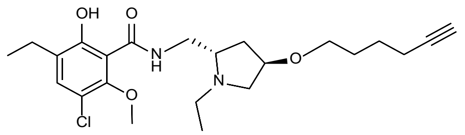

To a solution of (2*S*,4*R*)-1-ethyl-4-(hex-5-yn-1-yloxy)pyrrolidine-2-carboxamide (**6**, 0.47 g, 1.95 mmol) in THF (15 mL) $LiAlH_4$ in THF (1 M, 8.2 mL, 8.20 mmol) was added dropwise at 0 °C. The mixture was stirred under reflux conditions for 16 h, then cooled to 0°C. After dropwise addition of water, the mixture was filtered through a layered pad of celite and $MgSO_4$. Flushing the pad with MeOH (3x) and concentrating the combined organic phases *in vacuo* yielded a semi-solid crude which was extracted with $CH_2Cl_2$ under sonication (3x). Filtration and concentration of the combined extracts yielded a crude oil that was used in the next reaction without further purification.

To a solution of 3-chloro-5-ethyl-6-hydroxy-2-methoxybenzoic acid[2] (7, 0.45 g, 1.95 mmol) in $CH_2Cl_2$ (10 mL) were added HOBt (0.17 g, 2.15 mmol) and 3-(ethyliminomethyleneamino)-N,N-dimethylpropan-1-

amine hydrochloride (EDC*HCl, 0.24 g, 2.15 mmol) at 0 °C. After stirring at 0 °C for 1.5 h and at RT for 1 h, a solution of the crude oil in $CH_2Cl_2$ (10 mL) was added and the mixture was stirred at RT for 15 h. After concentrating the mixture *in vacuo*, the residue was dissolved in MeOH (5 mL) and 1M NaOH (0.5 mL) and stirred at 40 °C for 1.5 h. The mixture was partitioned between sat. $NaHCO_3$ solution and $CH_2Cl_2$, the aqueous phase was extracted with $CH_2Cl_2$ (2x) and the combined organic phases were dried ($MgSO_4$) and concentrated *in vacuo*. Flash column chromatography ($CH_2Cl_2$ / MeOH 100:1 + 0.2 % $NH_3$ 25 % aq. to 50:1 + 0.2 % $NH_3$ 25 % aq.) yielded **8** as a colorless oil (0.51 g 1.16 mmol, 59 %): $[\alpha]_D$ = +117,6 ° (c = 0.19 / MeOH, T = 28.0 °C); IR (film, NaCl):3356, 3303, 2972, 2936, 2873, 2800, 1635, 1599, 1577, 1526, 1434, 1370, 1296, 1220, 1117, 1081, 952 $cm^{-1}$; $^1H$ NMR ($CDCl_3$, 600 MHz): $\delta$ 13.81 (s, 1H), 8.83 (s, 1H), 7.22 (s, 1H), 3.98–3.92 (m, 1H), 3.87 (s, 3H), 3.82–3.72 (m, 1H), 3.56–3.45 (m, 1H), 3.44–3.35 (m, 2H), 3.35–3.26 (m, 1H), 2.99–2.82 (m, 2H), 2.62 (q, 2H, *J* = 7.6 Hz), 2.40–2.25 (m, 2H), 2.21 (dt, 2H, $J^1$ = 7.0 Hz, $J^2$ = 2.6 Hz), 1.94 (t, 1H, *J* = 2.6 Hz), 1.93–1.87 (m, 1H), 1.87–1.81 (m, 1H), 1.70–1.63 (m, 2H), 1.63–1.55 (m, 2H), 1.20 (t, 3H, *J* = 7.6 Hz), 1.12 (t, 3H, *J* = 7.0 Hz); $^{13}C$ NMR ($CDCl_3$, 90 MHz): $\delta$ 169.6, 160.2, 152.5, 133.0, 130.8, 116.1, 108.2, 84.2, 76.7, 68.6, 68.4, 67.0, 61.5, 60.7, 59.1, 47.4, 39.6, 35.5, 28.9, 25.3, 22.5, 18.2, 13.4; purity: S1C: 98.8 % ($t_R$ = 10.7 min); S2B: 98.2 % ($t_R$ = 13.0 min); ESI-MS (*m/z*): 437.8 $[M+H]^+$.

Methyl 4'-azido-(1,1'-biphenyl)-4-carboxylate (**9**)

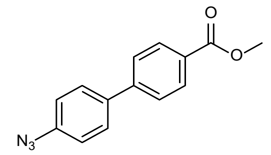


To a solution of 1-azido-4-iodobenzene (0.48 g, 1.95 mmol) and {4-[(benzyloxy)carbonyl]-phenyl}boronic acid (**17**, 0.50 g, 1.95 mmol) in DMF (10 mL) and MeOH (0.1 mL), $K_2CO_3$ (0.81 g, 5.86 mmol) and PdEnCat (0.15 g, 0.03 eq) were added. The mixture was stirred at 100 °C under microwave irradiation for 0.5 h, filtered and the solvent was evaporated *in vacuo.* Flash column chromatography (hexane / EtOAc 30:1 to 10:1) afforded **9** as a yellowish solid (0.40 g, 1.58 mmol, 81 %): mp 61 °C;$^1H$ NMR ($CDCl_3$, 600 MHz): $\delta$ 8.12–8.07 (m, 2H), 7.65–7.58 (m, 4H), 7.15–7.09 (m, 2H), 3.94 (s, 3H); $^{13}C$ NMR ($CDCl_3$, 150 MHz): $\delta$ TMS, ppm): 166.8, 144.5, 140.1, 136.7, 130.2, 129.0, 128.6, 126.7, 119.5, 52.0; ESI-MS (*m/z*): 226.3 $[M+H-N_2]^+$.

4'-Azido-(1,1'-biphenyl)-4-carboxylate (**10**)

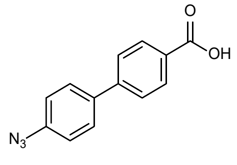

To a solution of methyl 4'-azido-(1,1'-biphenyl)-4-carboxylate (**9**, 0.15 g, 0.59 mmol) in ethanol (8 mL) was added NaOH (0.06 g, 1.59 mmol) in water (5 mL). After stirring under reflux conditions for 1 h, the mixture was allowed to cool to RT. Addition of aqueous HCl (37 %, 2mL) led to precipitation of **10** as a beige solid which was then filtered off and washed with water (0.14 g, 0.59 mmol, quant.): mp: n. d. (decomposition); IR (film, NaCl): 3447, 1655, 1634, 751 $cm^{-1}$; $^1H$ NMR (DMSO-$d_6$, 600 MHz): δ: 12.94 (s, 1H), 8.06–7.99 (m, 2H), 7.84–7.76 (m, 4H), 7.28–7.22 (m, 2H); $^{13}C$ NMR (DMSO-$d_6$, 90 MHz): δ 167.0, 143.1, 139.5, 135.7, 129.9, 129.5, 128.4, 126.4, 119.4; ESI-MS (*m/z*): 545.7 [2*M*+K]$^+$.

(3'*R*,5'*S*)-4'-(4-(4-((5-((3-Chloro-5-ethyl-6-hydroxy-2-methoxybenzamido)methyl)-1-ethylpyrrolidin-3-yl)oxy)butyl)-1*H*-1,2,3-triazol-1-yl)-[1,1'-biphenyl]-4-carboxylic acid (**11**)

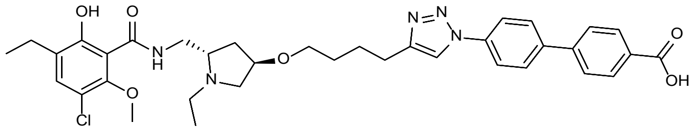


To a mixture of $H_2O$ (2 mL), $CH_2Cl_2$ (4 mL) and isopropanol (4 mL) (2'*S*,4'*R*)-3-chloro-5-ethyl-*N*-((1-ethyl-4-(hex-5-yn-1-yloxy)pyrrolidin-2-yl)methyl)-6-hydroxy-2-methoxybenzamide (**8**, 61.2 mg, 0.14 mmol), 4'-azido-(1,1'-biphenyl)-4-carboxylic acid (**10**, 50.0 mg, 0.21 mmol), sodium ascorbate (83.2 mg, 0.42 mmol) and $CuSO_4 \cdot 5H_2O$ (41.9 mg, 0.17 mmol) were subsequently added. After stirring at RT for 17 h, 0.1M ETDA solution (0.35 mL) was added and the mixture was concentrated to dryness *in vacuo*. Flash column chromatography ($CH_2Cl_2$ / MeOH 10:1 to 5:1) afforded **11** as a yellow oil (74.2 mg, 0.11 mmol, 78 %): $[\alpha]_D$ = -2.5° (c = 0.29 / $CH_2Cl_2$, T = 25.9 °C); IR (film, NaCl): 2962, 2934, 2871, 2833, 1611, 1589, 1577, 1529, 1507, 1453, 1437, 1381, 1344, 1289, 1260, 1208, 1156, 1134, 1038, 1048, 914, 837, 787 $cm^{-1}$; $^1H$ NMR ($CDCl_3$, 600 MHz, mixture of rotamers = 0.5 : 0.5): δ 14.6–13.0 (s, 0.5H), 9.21 (s, 0.5H), 9.08 (s, 0.5H), 8.29–8.08 (m, 2H), 8.69–7.87 (m, 3H), 7.69–7.53 (m, 4H), 7.22 (s, 0.5H), 7.21 (s, 0.5H), 7.04 (s, 1H), 7.03 (s, 1H), 6.44–6.41 (m, 2H), 4.14–4.08 (m, 0.5H), 4.08–4.01 (m, 0.5H), 3.89–3.90 (s, 1.5H), 3.89–3.87 (s, 1.5H), 3.71–3.62 (m, 1H), 3.59–3.52 (m, 0.5H), 3.52–3.47 (m, 0.5H), 3.47–3.35 (m, 2H), 3.20–3.12 (m. 0.5H), 3.10–3.00 (m, 0.5H), 2.89–2.80 (m, 1H), 2.79–2.68 (m, 1H), 2.59–2.53 (m, 4H), 2.38–2.26 (m, 1H), 2.20–2.14 (m, 0.5H), 2.14–2.07 (m, 0.5H), 2.04–1.93 (m, 1H), 1.86–1.57 (m, 3H), 1.17–1.13 (m, 6H); $^{13}C$ NMR ($CDCl_3$, 90 MHz, mixture of rotamers): δ 170.0 (1C), 160.1 (0.5C), 160.0 (0.5C), 158.9 (0.5C), 158.0 (0.5C), 152.9 (0.5C), 152.7 (0.5C), 148.7 (br, 1C), 143.0 (br, 1C), 140.5 (br, 1C), 136.7 (br, 1C),133.3 (0.5C), 133.2 (0.5C), 130.7 (0.5C), 130.5 (0.5C), 129.0 (0.5C), 128.4, (0.5C), 128.2 (0.5C), 126.7 (br, 1C),125.1 (0.5C), 120.6 (0.5C), 120.5 (0.5C), 118.8 (br, 1C), 116.2 (1C), 108.2 ($C_{min}$), 107.9 (0.5C), 103.8 (0.5C), 98.5 (0.5C), 77.1 (0.5C), 77.1 (0.5C), 76.7 (0.5C), 68.9 (0.5C), 68.7 (0.5C), 62.8 (0.5C), 62.7 (0.5C), 61.7 (0.5C), 61.6 (0.5C), 58.3 (0.5C), 58.1 (0.5C), 55.3 (0.5C), 55.3 (0.5C), 49.9 (0.5C), 49.4 (0.5C), 41.5 (0.5C), 40.1 (0.5C), 36.0 (0.5C), 35.5 (0.5C), 29.5 (0.5C), 29.3 (0.5C), 26.0 (0.5C), 25.5 (0.5C), 22.5 (0.5C), 22.4 (0.5C), 14.4 (0.5C), 13.4 (0.5C); purity: S1C: 93.3 % ($t_R$ = 16.3 min); ESI-MS (*m/z*): 676.9 [*M*+H]$^+$.

1-(Hex-5-ynyl)-4-(2-methoxyphenyl)piperazine (**13**)

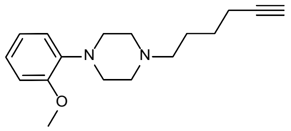

To a solution of 1-(2-methoxyphenyl)piperazine (0.5 g, 2.61 mmol) in DMF (10 mL) $K_2CO_3$ (0.72 g, 5.22 mmol), KI (0.22 g, 1.31 mmol) and 6-chlorohex-1-yne (0.48 mL, 3.92 mmol) were added. The mixture was stirred at 120 °C under microwave irradiation for 1.5 h. After dilution with $CH_2Cl_2$ and water the organic phase was washed with $H_2O$ (3×) and brine (1×), dried ($MgSO_4$) and concentrated *in vacuo*. Flash column chromatography (isopropanol / hexane 1:9 + 2 % $NEt_3$) afforded **13** as yellow solid (0.57 g, 81 %): mp 52.7–54.7 °C; IR (film, NaCl) 3295, 2941, 2814, 2116, 1593, 1500, 1451, 1240, 1136, 1029, 930 $cm^{-1}$; $^1H$ NMR ($CDCl_3$, 600 MHz): $\delta$ 7.04–6.82 (m, 4H), 3.86 (s, 3H), 3.17–3.05 (m, 4H), 2.69–2.60 (m, 4H), 2.46–2.38 (m, 2H), 2.23 (td, 2H, $J^1$ = 6.8 Hz, $J^2$ = 2.6 Hz), 1.95 (t, 1H, $J$ = 2.6 Hz), 1.72–1.50 (m, 4H); $^{13}C$ NMR ($CDCl_3$, 90 MHz): $\delta$ 151.8, 141.0, 122.4, 120.5, 117.8, 110.8, 83.9, 67.9, 57.8, 54.9, 53.0, 50.2, 26.0, 25.5, 17.9; ESI-MS (*m/z*): 273.2 $[M+H]^+$.

*N*-(Hex-5-yn-1-yl)-*N*-propyl-2,3-dihydro-1*H*-inden-2-amine (**14**)

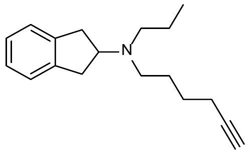

To a solution of *N*-propyl-2,3-dihydro-1*H*-inden-2-amine (0.6 g, 3.4 mmol) in DMF (10 mL), $K_2CO_3$ (0.95 g, 6.9 mmol), KI (0.28 g, 1.7 mmol) and 6-chlorohex-1-yne (0.62 mL, 5.1 mmol) were added. The mixture was stirred at 120 °C under microwave irradiation for 1.5 h and afterwards diluted with $CH_2Cl_2$ and brine. The aq. phase was extracted with $CH_2Cl_2$ (3×), dried ($MgSO_4$) and concentrated *in vacuo*. Flash column chromatography (EtOAc / hexane 3:7 + 2 % $NEt_3$) afforded **14** as brown oil (0.65 g, 75 %): IR (film, NaCl): 3308, 3022, 2953, 2870, 2806, 1697, 1460, 1377, 1254, 1139, 1083, 1025 $cm^{-1}$; $^1H$ NMR ($CDCl_3$, 600 MHz): $\delta$ 7.21–7.07 (m, 4H), 3.79–3.55 (m, 1H), 3.02 (dd, 2H , $J^1$ = 15.3 Hz, $J^2$ = 7.7 Hz), 2.89 (dd, 2H, $J^1$ = 15.4 Hz, $J^2$ = 8.8 Hz), 2.56 (t, 2H, $J$ = 8.8 Hz), 2.52–2.47 (m, 2H), 2.22 (td, 2H, $J^1$ = 11.1 Hz, $J^2$ = 9.9 Hz, $J^3$ = 6.1 Hz), 1.95 (t, 1H, $J$ = 2.6 Hz), 1.64–1.46 (m, 6H), 0.89 (t, 3H, $J$ = 7.4 Hz); $^{13}C$ NMR ($CDCl_3$, 90 MHz): $\delta$ 141.5, 125.8, 124.0, 83.9, 67.9, 62.7, 52.9, 50.3, 36.2, 26.0, 25.7, 19.9, 17.9, 11.5; APCI-MS (*m/z*): 256.2 $[M+H]^+$.

1-{4-[1-(4-Iodophenyl)triazol]butyl}-4-(2-methoxyphenyl)piperazine (**15**)

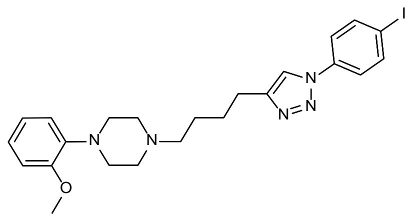

To a solution of 1-(hex-5-ynyl)-4-(2-methoxyphenyl)piperazine (**13**, 0.2 g, 0.73 mmol) in a 2:1 solution of $CH_2Cl_2$ / MeOH (6 mL) 1-azido-4-iodobenzene (0.2 g, 0.8 mmol) and $Cu(CH_3CN)_4PF_6$ (0.027 g, 0.07 mmol) were added. The mixture was stirred at 50 °C under microwave irradiation for 20 min, before the solvent was evaporated. Flash column chromatography (isopropanol / hexane 1:9 + 2 % $NEt_3$) afforded **15** as yellow solid (0.32 g, 84 %): mp 118.9–120.0 °C; IR (film, NaCl): 3140, 3061, 2939, 2814, 1592, 1497, 1450, 1398, 1306, 1240, 1134, 1027, 984, 912, 823.$cm^{-1}$; $^{1}H$ NMR ($CDCl_3$, 600 MHz): $\delta$ 7.89–7.77 (m, 2H), 7.76 (s, 1H), 7.54–7.42 (m, 2H), 7.04–6.77 (m, 4H), 3.86 (s, 3H), 3.27–3.04 (m, 4H), 2.84 (t, 2H, $J$ = 7.5 Hz), 2.84–2.66 (m, 4H), 2.59–2.50 (m, 2H), 1.85–1.76 (m, 2H), 1.74–1.65 (m, 2H); $^{13}C$ NMR ($CDCl_3$, 150 MHz): $\delta$ 152.3, 149.1, 141.3, 138.8, 136.9, 122.9, 121.5, 121.5, 118.6, 118.2, 111.2, 93.1, 58.4, 55.3, 53.5, 50.2, 27.3, 26.0, 25.4; APCI-MS (*m/z*): 518.2 $[M+H]^+$.

*N*-{4-[1-(4-Iodophenyl)-1*H*-1,2,3-triazol-4-yl]butyl}-*N*-propyl-2,3-dihydro-1*H*-inden-2-amine (**16**)

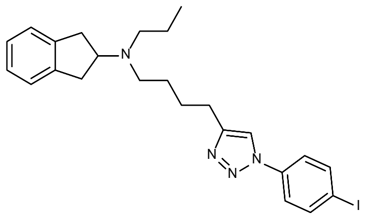

To a solution of *N*-(hex-5-yn-1-yl)-*N*-propyl-2,3-dihydro-1*H*-inden-2-amine (**14**, 0.5 g, 2.0 mmol) in a 2:1 solution of $CH_2Cl_2$ / MeOH (12 mL) 1-azido-4-iodobenzene (0.53 g, 2.2 mmol) and $Cu(CH_3CN)_4PF_6$ (0.07 g, 0.2 mmol) were added. The mixture was stirred at 50 °C under microwave irradiation for 20 min and afterwards the solvent was evaporated *in vacuo.* Flash column chromatography (EtOAc / hexane 1:2 + 2 % $NEt_3$) afforded **16** as beige solid (0.92 g, 94 %): mp 73.5–76.1 °C; IR (film, NaCl): 3141, 2935, 2867, 2805, 1587, 1496, 1397, 1230, 1042, 984, 823 $cm^{-1}$; $^{1}H$ NMR ($CDCl_3$, 600 MHz): $\delta$ 7.86–7.79 (m, 2H), 7.69 (s, 1H), 7.50–7.46 (m, 2H), 7.19–7.14 (m, 2H), 7.14-7.10 (m, 2H) 3.72–3.61 (m, 1H), 3.02 (dd, 2H , $J^1$ = 15.3 Hz, $J^2$ = 7.7 Hz), 2.89 (2H, dd, $J^1$ = 15.3 Hz, $J^2$ = 8.8 Hz), 2.82 (t, 2H, $J$ = 7.7 Hz), 2.62–2.56 (m, 2H), 2.54–2.47 (m, 2H), 1.78-1.71 (m, 2H), 1.64–1.56 (m, 2H), 1.54–1.46 (m, 2H), 0.88 (t, 3H, $J$ = 7.4 Hz); $^{13}C$ NMR ($CDCl_3$, 150 MHz): $\delta$ 149.2, 141.9, 138.8, 136.9, 126.3, 124.4, 121.9, 118.5, 93.1, 63.2, 53.4, 51.1, 36.6, 27.4, 26.9, 25.6, 20.2, 12.0; APCI-MS (*m/z*): 501.2 $[M+H]^+$.

{4-[(Benzyloxy)carbonyl]phenyl}boronic acid (**17**)[45]

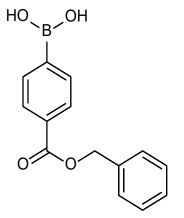


To a solution of 4-carboxyphenylboronic acid (2 g, 12 mmol) in DMF (150 mL) $KHCO_3$ (3 g, 0.03) was added and the mixture was heated under reflux conditions for 2 h, before benzyl bromide (1.8 mL, 14 mmol) was added and the mixture was stirred at 60 °C for 13 h. After evaporation of the solvent, the residue was diluted with water and extracted with EtOAc (3×). $K_2CO_3$ (1 g, 7 mmol) was added to the combined organic extracts, which were afterwards washed with water (2×), dried ($MgSO_4$) and concentrated *in vacuo*. Flash column chromatography (EtOAc / hexane 1:1) afforded **17** as colorless solid (1.32 g, 42 %): mp 205.5–207.5 °C; IR (film, NaCl): 2924, 2360, 2340, 1715, 1404, 1271, 1114, 1018, 697 $cm^{-1}$; $^1$H NMR ($CD_3OD$ / DMSO-$d_6$, 360 MHz): δ 8.10–7.99 (m, 2H), 7.99–7.90 (m, 2H), 7.53–7.33 (m, 5H), 5.36 (s, 2H); $^{13}$C NMR ($CD_3OD$ / DMSO-$d_6$, 90 MHz): δ 165.0, 134.7, 132.0, 131.6, 126.7, 126.6, 126.4, 126.3, 113.9, 64.9; ESI-MS (*m/z*): 256.2 [*M*+H]$^+$.

Benzyl-4'-(4-{4-[4-(2-methoxyphenyl)piperazine-1-yl]butyl}-1*H*-1,2,3-triazol-1-yl]-(1,1'-biphenyl)-4-carboxylate (**18**)

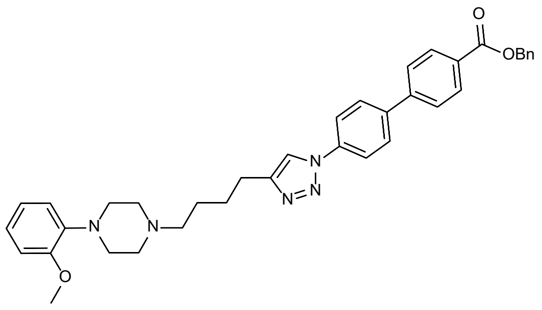


To a solution of 1-{4-[1-(4-iodophenyl)triazol]butyl}-4-(2-methoxyphenyl)piperazine (**15**, 0.5 g, 0.97 mmol) and {4-[(benzyloxy)carbonyl]-phenyl}boronic acid (**17**, 0.25 g, 0.97 mmol) in DMF (7 mL) and MeOH (10 drops), $K_2CO_3$ (0.4 g, 2.9 mmol) and PdEnCat (0.05 g, 0.03 eq) were added. The mixture was stirred at 100 °C under microwave irradiation for 0.5 h, filtered and the solvent was evaporated *in vacuo.* Flash column chromatography (EtOAc / hexane 3:1 + 2 % $NEt_3$) afforded **18** as yellowish solid (0.5 g, 86 %): mp 123.6–125.9 °C; IR (film, NaCl): 2940, 2816, 1716, 1607, 1529, 1499, 1454, 1377, 1272, 1241, 1181, 1110, 1028, 988, 913, 835 $cm^{-1}$; $^1$H NMR ($CDCl_3$, 360 MHz): δ 8.21–8.14 (m, 2H), 7.86–7.81 (m, 2H), 7.79 (s, 1H), 7.78-7.74 (m, 2H), 7.72–7.67 (m, 2H), 7.50–7.45 (m, 2H), 7.43–7.39 (m, 2H), 7.39–7.34 (m, 1H), 7.02–6.97 (m, 1H), 6.97–6.89 (m, 2H), 6.86 (dd, 1H, $J^1$ = 8.0 Hz, $J^2$ = 1.1 Hz), 5.40 (s, 2H), 3.86 (s, 3H), 3.14–3.07 (m, 4H), 2.87 (t, 2H, *J* = 7.6 Hz), 2.71-2.61 (m, 4H), 2.51–2.43

(m, 2H), 1.85–1.76 (m, 2H), 1.74–1.60 (m, 2H); $^{13}$C NMR ($CDCl_3$, 90 MHz): δ 166.1, 152.3, 148.9, 144.1, 141.3, 140.1, 137.0, 136.0, 130.4, 129.5, 128.6, 128.5, 128.3, 128.2, 127.0, 122.9, 121.0, 120.7, 118.7, 111.2, 66.8, 58.4, 55.3, 53.4, 50.5, 27.3, 26.4, 25.6; APCI-MS (*m/z*): 602.4 [*M*+H]$^+$.

Benzyl-4‘-(4-{4-[(2,3-dihydro-1*H*-inden-2-yl)(propyl)amino]butyl}-1*H*-1,2,3-triazol-1-yl)-(1,1‘-biphenyl)-4-carboxylate (**19**)

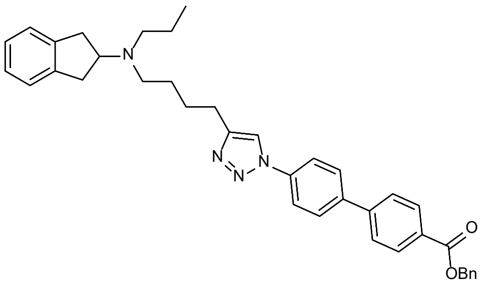


To a solution of *N*-{4-[1-(4-iodophenyl)-1*H*-1,2,3-triazol-4-yl]butyl}-*N*-propyl-2,3-dihydro-1*H*-inden-2-amine (**16**, 0.2 g, 0.4 mmol) and {4-[(benzyloxy)carbonyl]-phenyl}boronic acid (**17**, 0.1 g, 0.4 mmol) in DMF (7 mL) and MeOH (10 drops), $K_2CO_3$ (0.17 g, 1.2 mmol) and PdEnCat (0.03 g, 0.02 eq) were added. The mixture was stirred at 100 °C under microwave irradiation for 30 min, filtered and the solvent was evaporated *in vacuo.* Flash column chromatography (EtOAc / hexane 1:1 + 2 % $NEt_3$) afforded **19** as brown oil (0.12 g, 65 %): IR (film, NaCl): 2935, 1718, 1608, 1529, 1498, 1455, 1376, 1272, 1182, 1101, 1041, 988, 913, 834 cm$^{-1}$; $^1$H NMR ($CDCl_3$, 360 MHz): δ 8.21–8.12 (m, 2H), 7.88–7.80 (m, 2H), 7.77 (s, 1H), 7.76–7.73 (m, 2H), 7.71–7.62 (m, 2H), 7.51–7.44 (m, 2H), 7.44–7.31 (m, 3H), 7.22–7.05 (m, 4H), 5.40 (s, 2H), 3.74–3.56 (m, 1H), 3.02 (dd, 2H , $J^1$ = 15.4 Hz, $J^2$ = 7.7 Hz), 2.89 (dd, 2H, $J^1$ = 16.0 Hz, $J^2$ = 9.3 Hz), 2.84 (t, 2H, *J* = 7.6 Hz), 2.64–2.56 (m, 2H), 2.56–2.46 (m, 2H), 1.84–1.70 (m, 2H), 1.70–1.56 (m, 2H), 1.55–1.43 (m, 2H), 0.88 (t, 3H, *J* = 7.3 Hz); $^{13}$C NMR ($CDCl_3$, 90 MHz): δ 166.1, 149.0, 144.2, 141.8, 140.1, 137.0, 136.0, 130.4, 129.5, 128.6, 128.5, 128.3, 128.2, 127.0, 126.3, 124.4, 120.7, 118.7, 66.8, 63.1, 53.3, 51.0, 36.5, 27.4, 26.7, 25.6, 20.0, 11.8; APCI-MS (*m/z*): 585.4 [*M*+H]$^+$.

4‘-(4-{4-[4-(2-Methoxyphenyl)piperazine-1-yl]butyl}-1*H*-1,2,3-triazole-1-yl)-(1,1‘-biphenyl)-4-carboxylic acid (**20**)

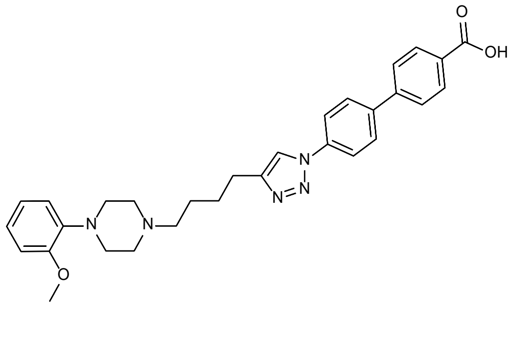

To a solution of benzyl-4‘-(4-{4-[4-(2-methoxyphenyl)piperazine-1-yl]butyl}-1*H*-1,2,3-triazol-1-yl]-(1,1‘-biphenyl)-4-carboxylate (**18**, 0.48 g, 9.8 mmol) in MeOH (15 mL) and $CH_2Cl_2$ (10 mL), Pd / C (0.06 g, 10wt-%) was added. The flask was evacuated, flushed with $H_2$ (3×) and the mixture was stirred at RT for 30 min. The mixture was filtered through a pad of celite which was then washed thoroughly with MeOH and $CH_2Cl_2$. Evaporation of all volatiles yielded **20** as beige solid (0.46 g, quant.), which was used in the next step without further purification: mp 179.9–182.0 °C; IR (film, NaCl): 2947, 1693, 1607, 1530, 1501, 1244, 1118, 1023, 913, 834 $cm^{-1}$. $^1H$ NMR ($CDCl_3$ / HCOOH, 360 MHz): $\delta$ 8.21–8.16 (m, 2H), 8.15–8.10 (m, 3H), 7.84–7.80 (m, 2H), 7.76–7.72 (m, 2H), 7.28–7.23 (m, 1H), 7.21 (dd, 1H, $J^1$ = 7.9 Hz, $J^2$ = 1.2 Hz), 7.04–6.96 (m, 2H), 3.90 (s, 3H), 3.84–3.54 (m, 8H), 3.32–3.26 (m, 2H), 2.93 (t, 2H, *J* = 7.3 Hz), 1.98–1.90 (m, 2H), 1.90–1.82 (m, 2H);$^{13}C$ NMR ($CDCl_3$ / HCOOH, 90 MHz): $\delta$ 171.7, 152.1, 147.3, 144.9, 140.8, 137.3, 136.4, 130.9, 128.7, 128.5, 127.2, 125.8, 121.4, 121.0, 120.5, 119.3, 111.7, 57.0, 55.5, 52.1, 48.0, 25.9, 24.1, 23.0; APCI-MS (*m/z*): 512.4 $[M+H]^+$.

4‘-(4-{4-[(2,3-Dihydro-1*H*-inden-2-yl)(propyl)amino]butyl}-1*H*-1,2,3-triazol-1-yl)-(1,1‘-biphenyl)-4-carboxylic acid (**21**)

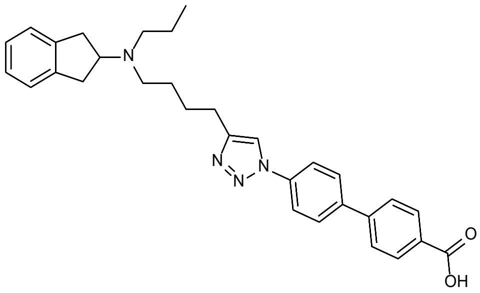


To a solution of benzyl-4‘-(4-{4-[(2,3-dihydro-1*H*-inden-2-yl)(propyl)amino]butyl}-1*H*-1,2,3-triazol-1-yl)-(1,1‘-biphenyl)-4-carboxylate (**19**, 1.0 g, 1.7 mmol) in MeOH (40 mL) and $CH_2Cl_2$ (5 mL), Pd / C (0.2 g, 10wt-%) was added. The flask was evacuated, flushed with $H_2$ (3×) and the mixture was stirred at RT for 8 h. The mixture was filtered through a pad of celite which was then washed thoroughly with MeOH and $CH_2Cl_2$. Evaporation of all volatiles yielded **21** as brown solid (0.82 g, quant), which used in the next step without further purification: mp 111–113 °C; IR (film, NaCl): 3410, 2928, 1702, 1604, 1529, 1375, 1231, 1044, 989, 838 $cm^{-1}$; $^1H$ NMR ($CDCl_3$, 600 MHz): $\delta$ 8.06 (s, 1H), 7.98 (d, 2H, *J* = 8.31 Hz), 7.50 (d, 2H, *J* = 8.7 Hz), 7.43–7.39 (m, 4H), 7.23–7.16 (m, 4H), 4.05 (quin, 1H, *J* = 8.1 Hz), 3.46 (dd, 2H , $J^1$ = 15.5 Hz, $J^2$ = 8.3 Hz), 3.25 (dd, 2H, $J^1$ = 15.7 Hz, $J^2$ = 8.1 Hz), 3.14–3.00 (m, 4H), 2.99–2.90 (m, 2H), 1.97–1.86 (m, 4H), 1.81–1.72 (m, 2H), 1.00 (t, 3H, *J* = 7.4 Hz); $^{13}C$ NMR ($CDCl_3$, 150 MHz): $\delta$ 173.0, 149.1, 143.0, 141.6, 141.1, 137.8, 136.7, 131.5, 129.5, 128.6, 127.8, 125.9, 122.0, 121.3, 64.2, 52.3, 52.2, 36.3, 27.2, 26.3, 25.5, 17.4, 13.0; APCI-MS (*m/z*) 495.3 $[M+H]^+$.

*N*-(2-methoxyethyl)-4'-(4-{4-[4-(2-methoxyphenyl)piperazin-1-yl]butyl}-1*H*-1,2,3-triazol-1-yl)-(1,1'-biphenyl)-4-carboxamide (**2g**)

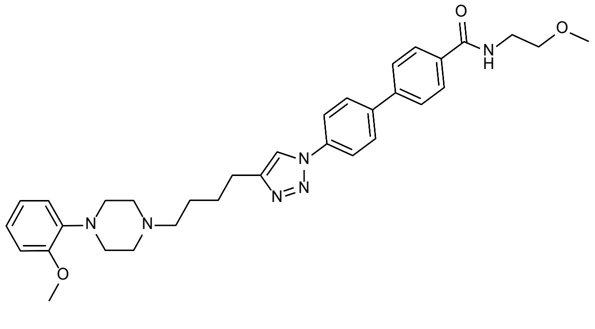

To a solution of 4'-(4-{4-[4-(2-methoxyphenyl)piperazine-1-yl]butyl}-1*H*-1,2,3-triazole-1-yl)-(1,1'-biphenyl)-4-carboxylic acid (**20**, 0.01 g, 0.02 mmol) and 2-methoxyethylamine (5 μL, 0.06 mmol) in DMF (2 mL) DIPEA (8 μL, 0.05 mmol) and HATU (0.01 g, 0.02 mmol) were added. The mixture was stirred at RT for 3 h. After evaporation of the solvent, the residue was diluted with water and extracted with $CHCl_3$ (2×). The combined organic extracts were dried ($MgSO_4$) and concentrated *in vacuo*. Purification via prep. HPLC [C18]: eluent: MeOH (A) and 0.1 % $HCO_2H$ in $H_2O$ (B) applying a linear gradient starting from 10 % A in 90 % B to 80 % A in 20 % B in 15.0 min, FR: 32.0 mL $min^{-1}$ afforded **2g** as a colorless oil (0.046 g, 43 %): IR (film, NaCl) IR (film, NaCl) 3300, 3150, 1633, 1607, 1550, 1501, 1310, 1118, 1023, 913, 834 $cm^{-1}$. $^1H$ NMR ($CD_3OD$, 360 MHz) δ 8.40 (s, 1H), 7.98–7.92 (m, 4H), 7.92–7.87 (m, 2H), 7.84–7.78 (m, 2H), 7.04–6.99 (m, 1H), 6.99–6.93 (m, 2H), 6.92–6.87 (m, 1H), 3.85 (s, 3H), 3.61–3.50 (m, 2H), 3.45–3.34 (m, 2H), 3.20–3.07 (m, 4H), 2.96–2.77 (m, 6H), 2.76–2.61 (m, 2H), 1.88–1.77 (m, 2H), 1.77–1.67 (m, 2H). $^{13}C$ NMR ($CD_3OD$, 150 MHz) δ 168.4, 152.5, 148.5, 142.5, 140.4, 140.3, 136.7, 133.5, 128.1, 127.7, 126.6, 123.4, 120.7, 120.3, 120.0, 118.1, 111.4, 70.6, 57.6, 57.5, 54.5, 52.7, 49.6, 39.3, 26.7, 25.0, 24.6. MS (ESI) *m/z* 570.2 $[M+H]^+$.

4'-(4-{4-[(2,3-dihydro-1*H*-inden-2-yl)(propyl)amino]butyl}-1*H*-1,2,3-triazol-1-yl)-*N*-(2-methoxyethyl)-(1,1'-biphenyl)-4-carboxamide (**3g**)

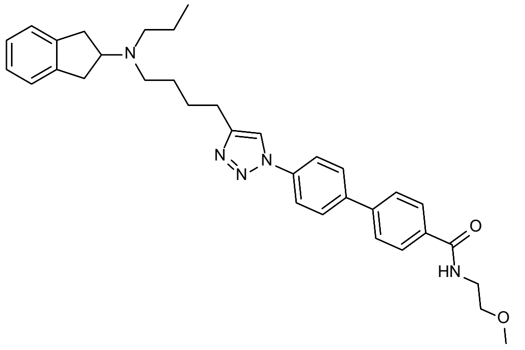

To a solution of 4'-(4-{4-[(2,3-dihydro-1*H*-inden-2-yl)(propyl)amino]butyl}-1*H*-1,2,3-triazol-1-yl)-(1,1'-biphenyl)-4-carboxylic acid (**21**, 0.01 g, 0.02 mmol) and methoxyethylamine (5 μL, 0.06 mmol) in DMF

(2 mL), DIPEA (8 μL, 0.05 mmol) and HATU (0.01 g, 0.02 mmol) were added. The mixture was stirred at RT for 3 h. After evaporation of the solvent, the residue was diluted with water and extracted with $CHCl_3$ (2×). The combined organic extracts were dried ($MgSO_4$) and concentrated *in vacuo*. Purification via prep. HPLC [C18]: eluent: MeOH (A) and 0.1 % $HCO_2H$ in $H_2O$ (B) applying a linear gradient starting from 5 % A in 95 % B to 60 % A in 40 % B in 12.0 min, FR: 32.0 mL $min^{-1}$ afforded **3g** as a colorless oil (0.044 g, 41 %): IR (film, NaCl) 3290, 3101, 1682, 1604, 1529, 1310, 1231, 1044, 989, 838 $cm^{-1}$. $^1H$ NMR ($CD_3OD$, 600 MHz) δ 7.92–7.87 (m, 2H), 7.85–7.80 (m, 2H), 7.80–7.73 (m, 3H), 7.73–7.66 (m, 2H), 7.20–7.08 (m, 4H), 6.58 (t, 1H, J = 5.3 Hz), 3.73–3.65 (m, 3H), 3.63–3.56 (m, 2H), 3.42 (s, 3H), 3.03 (dd, 2H, $J^1$ = 15.4 Hz, $J^2$ = 7.7 Hz), 2.90 (dd, 2H, $J^1$ = 14.0 Hz, $J^2$ = 7.3 Hz), 2.85 (t, 2H, J = 6.1 Hz), 2.65–2.56 (m, 2H), 2.56–2.46 (m, 2H), 1.84–1.72 (m, 2H), 1.72–1.57 (m, 2H), 1.55–1.43 (m, 2H), 0.89 (t, 3H, J = 7.3 Hz). $^{13}C$ NMR ($CD_3OD$, 90 MHz) δ 168.3, 148.3, 142.5, 140.3, 140.1, 136.6, 133.5, 128.1, 127.6, 126.6, 126.4, 123.9, 120.3, 120.0, 70.6, 63.0, 57.0, 52.8, 50.6, 39.3, 35.6, 26.5, 24.4, 24.3, 18.2, 10.4. MS (ESI) (*m/z*) 553.3 [*M*+H]$^+$.

<u>[1-(4'-{4-[4-({(3R,5S)-5-[(3-chloro-5-ethyl-6-hydroxy-2-methoxybenzamido)methyl]-1-ethylpyrrolidin-3-yl}oxy)butyl]-1H-1,2,3-triazol-1-yl}-(1,1'-biphenyl)-4-yl)-1-carboxamido-(4,7,10,13,16,19-hexaoxaheneicosanoylamido)]-L-arginyl-L-arginyl-L-prolyl-L-tyrosyl-L-isoleucyl-L-leucine (**1a**)</u>

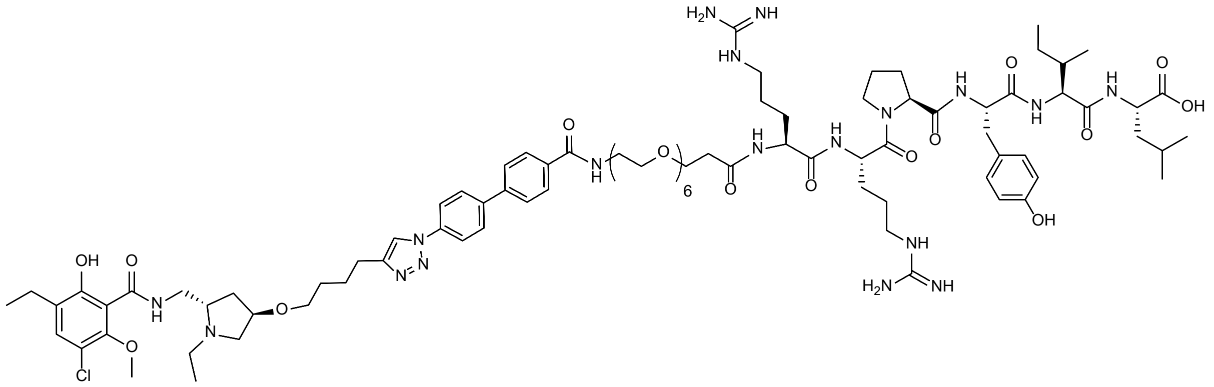

The heterobivalent ligand was synthesized according to method 1 using 1x Fmoc-NH-PEG$_6$-propionic acid and **11**. Purification: eluent: $CH_3CN$ (A) and 0.1 % HCOOH in $H_2O$ (B) applying a linear gradient starting from 5 % A in 95 % B to 42 % A in 58 % B in 15.0 min. FR: 32.0 mL $min^{-1}$, $t_R$: 14.2 min; purity: S1D: 98.8 % ($t_R$ = 14.0 min); S2D: >99 % ($t_R$ = 10.2 min); ESI-MS (*m/z*): 604.9 [(*M*+3H)/3]$^{3+}$; HRMS-ESI (*m/z*): [*M* +H]$^+$: calcd. for $C_{89}H_{133}ClN_{18}O_{20}$: 1809.9705, found: 1809.9698.

[1-(4'-{4-[4-({(3R,5S)-5-[(3-chloro-5-ethyl-6-hydroxy-2-methoxybenzamido)methyl]-1-ethylpyrrolidin-3-yl}oxy)butyl]-1H-1,2,3-triazol-1-yl}-(1,1'-biphenyl)-4-yl)-1-carboxamido-[bis-(4,7,10,13,16,19-hexaoxaheneicosanoylamido)]]-L-arginyl-L-arginyl-L-prolyl-L-tyrosyl-L-isoleucyl-L-leucine (**1b**)

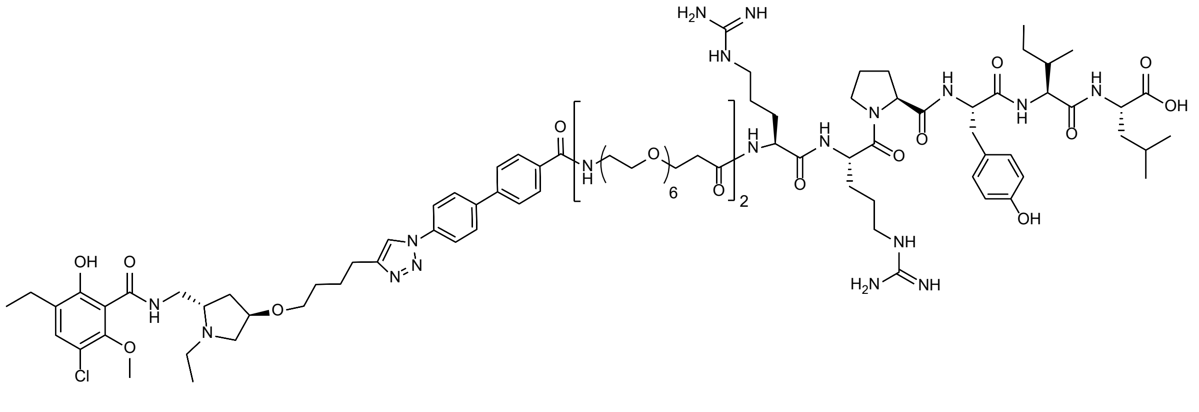

The heterobivalent ligand was synthesized according to method 1 using 2× Fmoc-NH-PEG$_6$-propionic acid and **11**. Purification: eluent: $CH_3CN$ (A) and 0.1 % HCOOH in $H_2O$ (B) applying a linear gradient starting from 5 % A in 95 % B to 42 % A in 58 % B in 15.0 min. FR: 32.0 mL min$^{-1}$. $t_R$: 13.6 min; purity: S1D: 98.8 % ($t_R$ = 14.5 min); S2D: 98.6 % ($t_R$ = 10.6 min); ESI-MS (*m/z*): 716.8 [(*M*+3H)/3]$^{3+}$; HRMS-ESI (*m/z*): [*M* +H]$^+$: calcd. for $C_{104}H_{162}ClN_{19}O_{27}$: 2145.1649, found: 2145.1674.

[1-(4'-{4-[4-({(3R,5S)-5-[(3-chloro-5-ethyl-6-hydroxy-2-methoxybenzamido)methyl]-1-ethylpyrrolidin-3-yl}oxy)butyl]-1H-1,2,3-triazol-1-yl}(1,1'-biphenyl)-4-yl)-1-carboxamido-[tris-(4,7,10,13,16,19-hexaoxaheneicosanoylamido)]]-L-arginyl-L-arginyl-L-prolyl-L-tyrosyl-L-isoleucyl-L-leucine (**1c**)

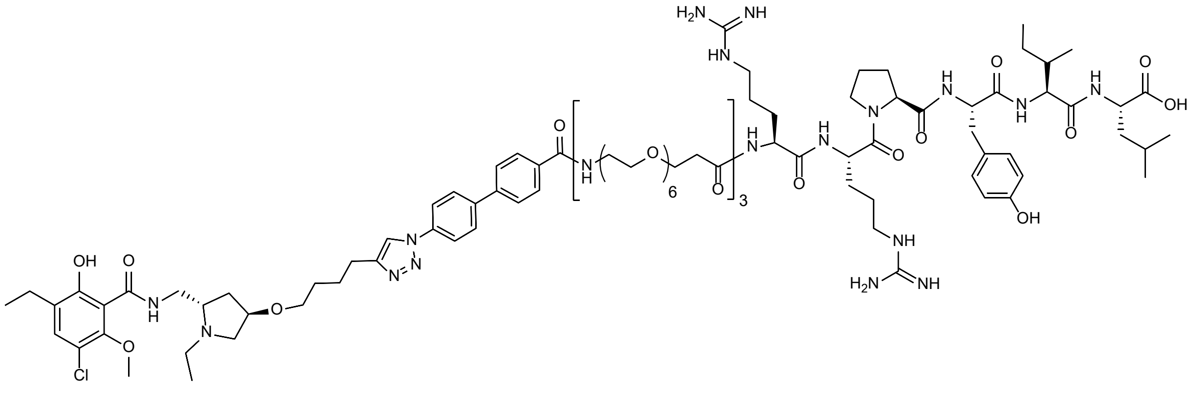

The heterobivalent ligand was synthesized according to method 1 using 3× Fmoc-NH-PEG$_6$-propionic acid and **11**. Purification: eluent: $CH_3CN$ (A) and 0.1 % HCOOH in $H_2O$ (B) applying a linear gradient starting from 5 % A in 95 % B to 42 % A in 58 % B in 15.0 min. FR: 32.0 mL min$^{-1}$. $t_R$: 14.4 min; purity: S1D: 97 % ($t_R$ = 14.2 min); S2D: 97 % ($t_R$ = 10.4 min); ESI-MS (*m/z*): 621.8 [(*M*+4H)/4]$^{4+}$; HRMS-ESI (*m/z*): [*M* +H]$^+$: calcd. for $C_{104}H_{192}ClN_{20}O_{34}$: 2480.3593, found: 2480.3573.

[1-(4'-{4-[4-({(3R,5S)-5-[(3-chloro-5-ethyl-6-hydroxy-2-methoxybenzamido)methyl]-1-ethylpyrrolidin-3-yl}oxy)butyl]-1H-1,2,3-triazol-1-yl}-(1,1'-biphenyl)-4-yl)-1-carboxamido-[tetrakis-(4,7,10,13,16,19-hexaoxaheneicosanoylamido)]]-L-arginyl-L-arginyl-L-prolyl-L-tyrosyl-L-isoleucyl-L-leucine (**1d**)

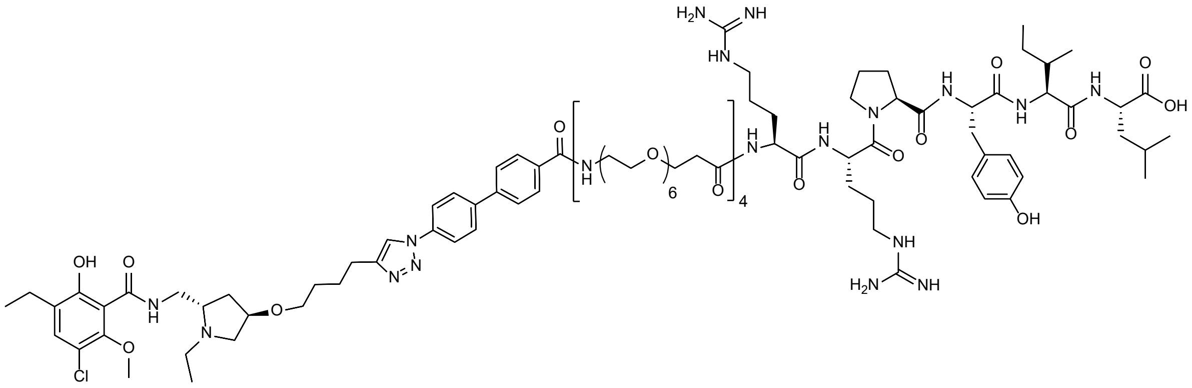

The heterobivalent ligand was synthesized according to method 1 using 4× Fmoc-NH-PEG$_6$-propionic acid and **11**. Purification: eluent: $CH_3CN$ (A) and 0.1 % HCOOH in $H_2O$ (B) applying a linear gradient starting from 5 % A in 95 % B to 42 % A in 58 % B in 15.0 min. FR: 32.0 mL min$^{-1}$. $t_R$: 14.9 min; purity: S3A: 98.8 % ($t_R$ = 47.3 min); S4A: 99.7 % ($t_R$ = 30.5 min); ESI-MS (*m/z*): 705.04 [(*M*+4H)/4]$^{4+}$; HRMS-ESI (*m/z*): [*M* +H]$^+$: calcd. for $C_{134}H_{220}ClN_{21}O_{41}$: 2815.55369, found: 2815.55602.

{1-[4'-(4-{4-[4-(2-methoxyphenyl)piperazin-1-yl]butyl}-1H-1,2,3-triazol-1-yl)-(1,1'-biphenyl)-4-yl]-1-carboxamido-(4,7,10,13,16,19-hexaoxaheneicosanoylamido)}-L-arginyl-L-arginyl-L-prolyl-L-tyrosyl-L-isoleucyl-L-leucine (**2a**)

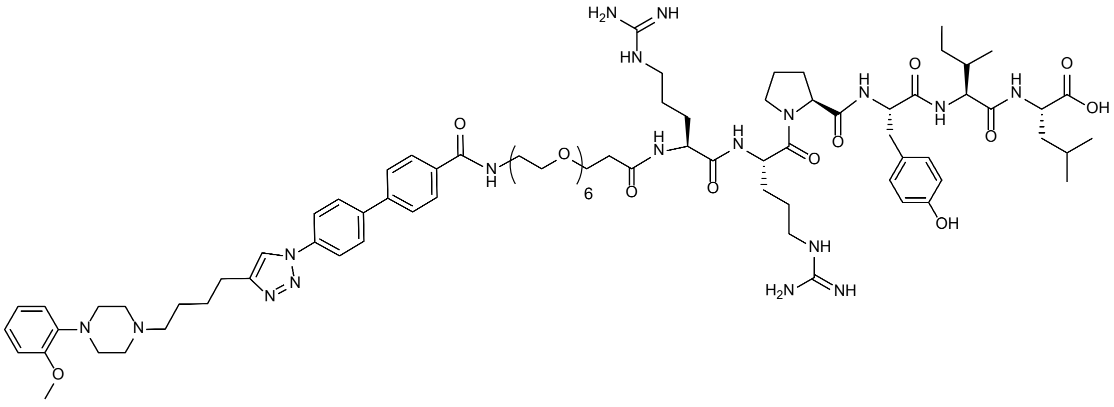

The heterobivalent ligand was synthesized according to method 1 using Fmoc-NH-PEG$_6$-propionic acid and **20**. Purification: eluent: $CH_3CN$ (A) and 0.1 % HCOOH in $H_2O$ (B) applying a linear gradient starting from 5 % A in 95 % B to 42 % A in 58 % B in 15.0 min. FR: 32.0 mL min$^{-1}$. $t_R$: 13.4 min; purity: S1A: >99 % ($t_R$ = 21.5 min); S2A: >99 % ($t_R$ = 22.3 min); ESI-MS (*m/z*): 549.6 [(*M*+3H)/3]$^{3+}$; HRMS-ESI (*m/z*) [(*M*+Na+H)/2]$^{2+}$ calcd. for $C_{83}H_{124}N_{18}O_{17}$: 834.4678, found: 834.4684.

{1-[4'-(4-{4-[4-(2-methoxyphenyl)piperazin-1-yl]butyl}-1H-1,2,3-triazol-1-yl)-(1,1'-biphenyl)-4-yl]-1-carboxamido-[bis-(4,7,10,13,16,19-hexaoxaheneicosanoylamido)]}-L-arginyl-L-arginyl-L-prolyl-L-tyrosyl-L-isoleucyl-L-leucine (**2b**)

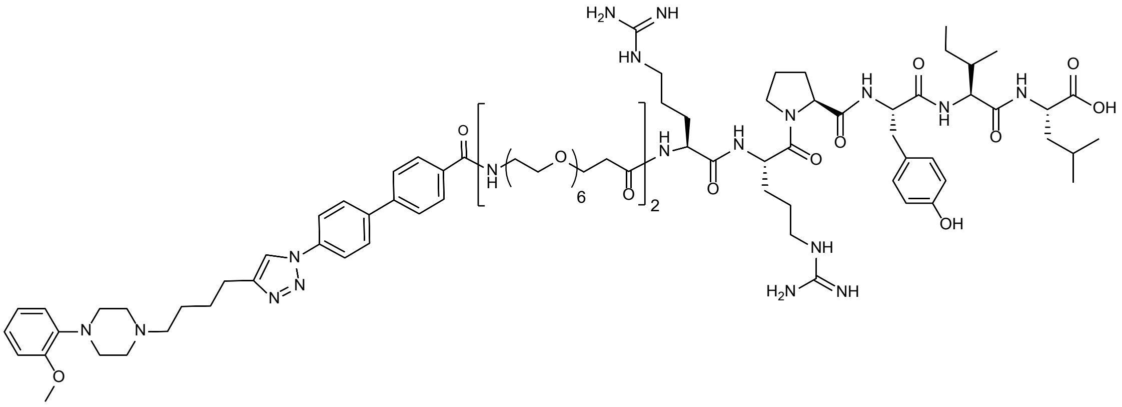

The heterobivalent ligand was synthesized according to method 1 using 2× Fmoc-NH-PEG$_6$-propionic acid and **20**. Purification: eluent: $CH_3CN$ (A) and 0.1 % HCOOH in $H_2O$ (B) applying a linear gradient starting from 5 % A in 95 % B to 42 % A in 58 % B in 15.0 min. FR: 32.0 mL min$^{-1}$. $t_R$: 13.3 min; purity: S1A: >99 % ($t_R$ = 22.2 min); S2A: >99 % ($t_R$ = 22.9 min); ESI-MS (*m/z*): 661.5 [(*M*+3H)/3]$^{3+}$; HRMS-ESI (*m/z*): [(*M*+Na+H)/2]$^{2+}$ calcd. for $C_{98}H_{153}N_{19}O_{24}$: 1002.5666, found: 1002.5668.

{1-[4'-(4-{4-[4-(2-methoxyphenyl)piperazin-1-yl]butyl}-1H-1,2,3-triazol-1-yl)-(1,1'-biphenyl)-4-yl]-1-carboxamido-[tris-(4,7,10,13,16,19-hexaoxaheneicosanoylamido)]}-L-arginyl-L-arginyl-L-prolyl-L-tyrosyl-L-isoleucyl-L-leucine (**2c**)

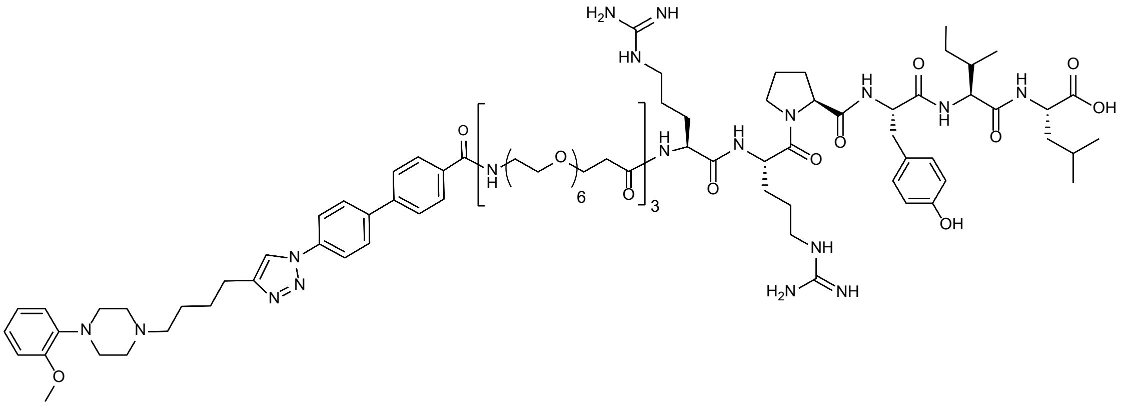

The heterobivalent ligand was synthesized according to method 1 using 3× Fmoc-NH-PEG$_6$-propionic acid and **20**. Purification: eluent: $CH_3CN$ (A) and 0.1 % HCOOH in $H_2O$ (B) applying a linear gradient starting from 5 % A in 95 % B to 42 % A in 58 % B in 15.0 min. FR: 32.0 mL min$^{-1}$. $t_R$: 12.7 min; purity: S1B: >99 % ($t_R$ = 17.3 min); S2B: >99 % ($t_R$ = 18.1 min); ESI-MS (*m/z*): 774.1 [(*M*+3H)/3]$^{3+}$; HRMS-ESI (*m/z*): [*M*+H]$^{+}$ calcd. for $C_{113}H_{182}N_{20}O_{31}$: 2317.3383, found: 2317.3294.

{1-[4'-(4-{4-[4-(2-methoxyphenyl)piperazin-1-yl]butyl}-1H-1,2,3-triazol-1-yl)-(1,1'-biphenyl)-4-yl]-1-carboxamido-[tetrakis-(4,7,10,13,16,19-hexaoxaheneicosanoylamido)]}-L-arginyl-L-arginyl-L-prolyl-L-tyrosyl-L-isoleucyl-L-leucine (**2d**)

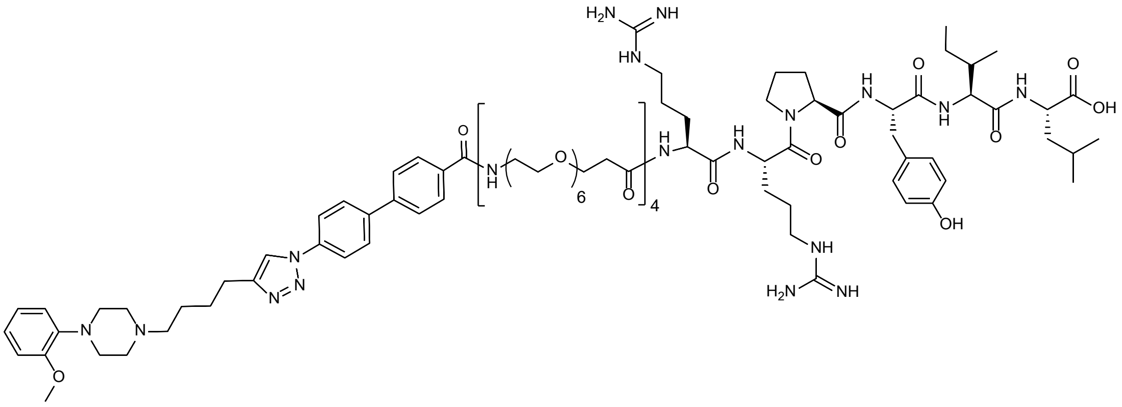

The heterobivalent ligand was synthesized according to method 1 using 4× Fmoc-PEG$_5$-OH and **20**. Purification: eluent: $CH_3CN$ (A) and 0.1 % HCOOH in $H_2O$ (B) applying a linear gradient starting from 5 % A in 95 % B to 38 % A in 62 % B in 15.0 min. FR: 32.0 mL min$^{-1}$. $t_R$: 13.5 min; purity: S1C: >99 % ($t_R$ = 18.4 min); S2C: >99 % ($t_R$ = 17.1 min); MS (ESI) *m/z* 664.0 [(*M*+4H)/4]$^{4+}$; HRMS-ESI *m/z* [*M*+H]$^+$ calcd.for $C_{128}H_{212}N_{21}O_{38}$: 2651.52967, found: 2651.52624.

{1-[4'-(4-{4-[4-(2-methoxyphenyl)piperazin-1-yl]butyl}-1H-1,2,3-triazol-1-yl)-(1,1'-biphenyl)-4-yl]-1-carboxamido-(4,7,10,13,16,19-hexaoxaheneicosanoylamido)}-L-arginyl-L-arginyl-L-prolyl-*N*-[2-(4-hydroxyphenyl)ethyl]glycine-L-isoleucyl-L-leucine (**2e**)

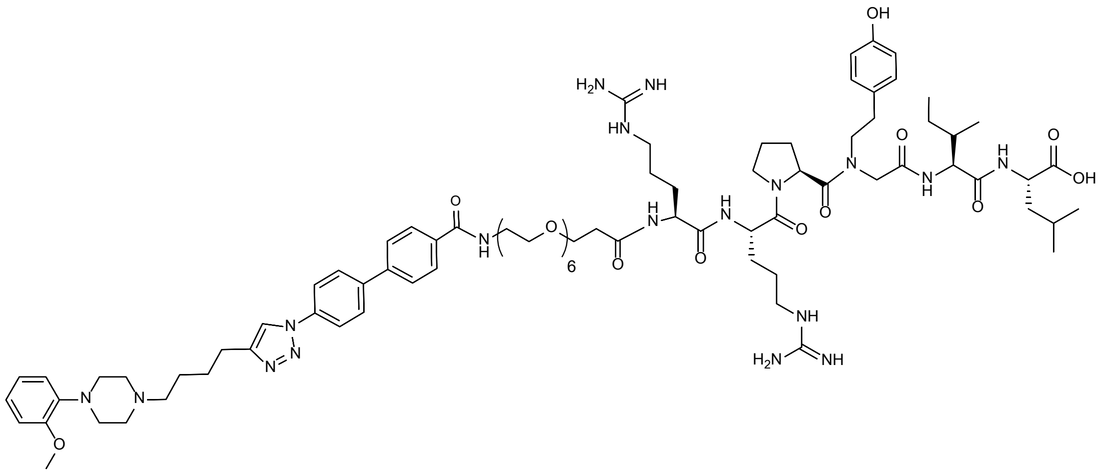

The heterobivalent ligand was synthesized according to method 1 using 2× Fmoc-NH-PEG$_6$-propionic acid and **20**. Purification: eluent: $CH_3CN$ (A) and 0.1 % HCOOH in $H_2O$ (B) applying a linear gradient starting from 5 % A in 95 % B to 42 % A in 58 % B in 15.0 min. FR: 32.0 mL min$^{-1}$. $t_R$: 13.2 min; purity: S1A: > 99 % ($t_R$ = 21.7 min); S2A: >99 % ($t_R$ = 22.7 min); MS (ESI) *m/z* 554.3 [(*M*+3H)/3]$^{3+}$; HRMS-ESI (*m/z*): [(*M*+Na+2H)/3]$^{3+}$ calcd. for $C_{84}H_{126}N_{18}O_{17}$: 561.3195, found: 561.3201.

{1-[4'-(4-{4-[4-(2-methoxyphenyl)piperazin-1-yl]butyl}-1H-1,2,3-triazol-1-yl)-(1,1'-biphenyl)-4-yl]-1-carboxamido-[bis-(4,7,10,13,16,19-hexaoxaheneicosanoylamido)]}-L-arginyl-L-arginyl-L-prolyl-*N*-[2-(4-hydroxyphenyl)ethyl]glycine-L-isoleucyl-L-leucine (**2f**)

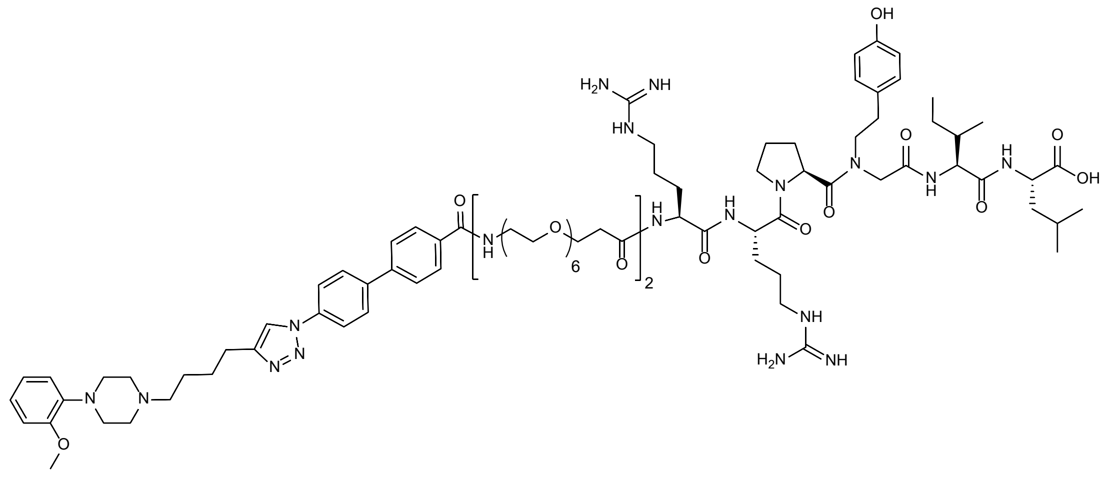

The heterobivalent ligand was synthesized according to method 1 using 2× Fmoc-NH-PEG$_6$-propionic acid and **20**. Purification: eluent: $CH_3CN$ (A) and 0.1 % HCOOH in $H_2O$ (B) applying a linear gradient starting from 5 % A in 95 % B to 42 % A in 58 % B in 15.0 min. FR: 32.0 mL min$^{-1}$. $t_R$: 13.6 min; purity: S1A: >99 % ($t_R$ = 22.4 min); S2A: >99 % ($t_R$ = 23.2 min); HRMS-ESI (*m/z*): [(*M*+Na+2H)/3]$^{3+}$ calcd. for $C_{99}H_{155}N_{19}O_{24}$: 673.3853, found: 673.3876.

{1-[4'-(4-{4-[(2,3-dihydro-1H-inden-2-yl)(propyl)amino]butyl}-1H-1,2,3-triazol-1-yl)-(1,1'-biphenyl)-4-yl]-1-carboxamido-(4,7,10,13,16,19-hexaoxaheneicosanoylamido)}-L-arginyl-L-arginyl-L-prolyl-L-tyrosyl-L-isoleucyl-L-leucine (**3a**)

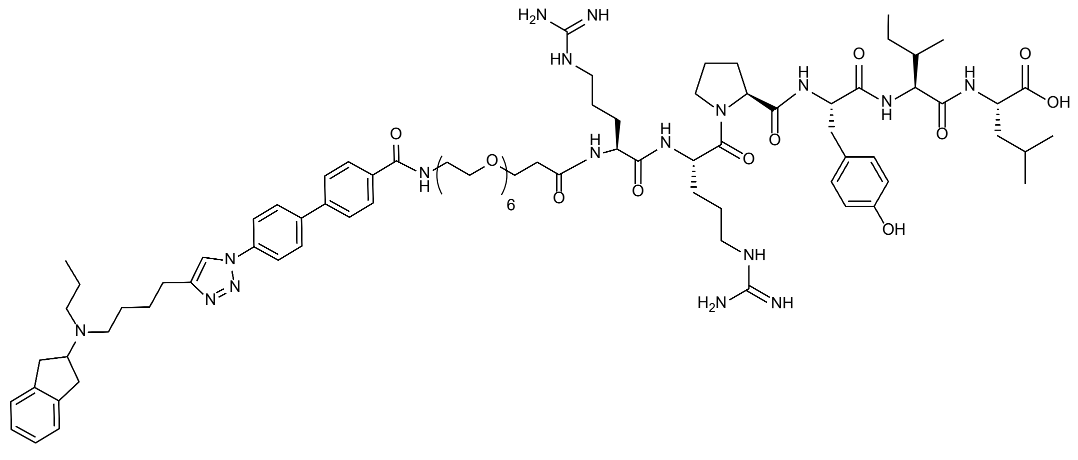

The heterobivalent ligand was synthesized according to method 1 using Fmoc-PEG$_5$-OH and **21**. Purification: eluent: $CH_3CN$ (A) and 0.1 % HCOOH in $H_2O$ (B) applying a linear gradient starting from 5 % A in 95 % B to 42 % A in 58 % B in 15.0 min. FR: 32.0 mL min$^{-1}$. $t_R$: 13.4 min; purity: S1A: >99 % ($t_R$

= 22.4 min); S2A: >99 % ($t_R$ = 23.9 min); ESI-MS (*m/z*): 544.0 [(*M*+3H)/3]$^{3+}$; HRMS-ESI (*m/z*): [*M*+H]$^+$ calcd. for $C_{84}H_{125}N_{17}O_{16}$: 1628.9563, found: 1628.9582.

{1-[4'-(4-{4-[(2,3-dihydro-1H-inden-2-yl)(propyl)amino]butyl}-1H-1,2,3-triazol-1-yl)-(1,1'-biphenyl)-4-yl]-1-carboxamido-[bis-(4,7,10,13,16,19-hexaoxaheneicosanoylamido)]}-L-arginyl-L-arginyl-L-prolyl-L-tyrosyl-L-isoleucyl-L-leucine (**3b**)

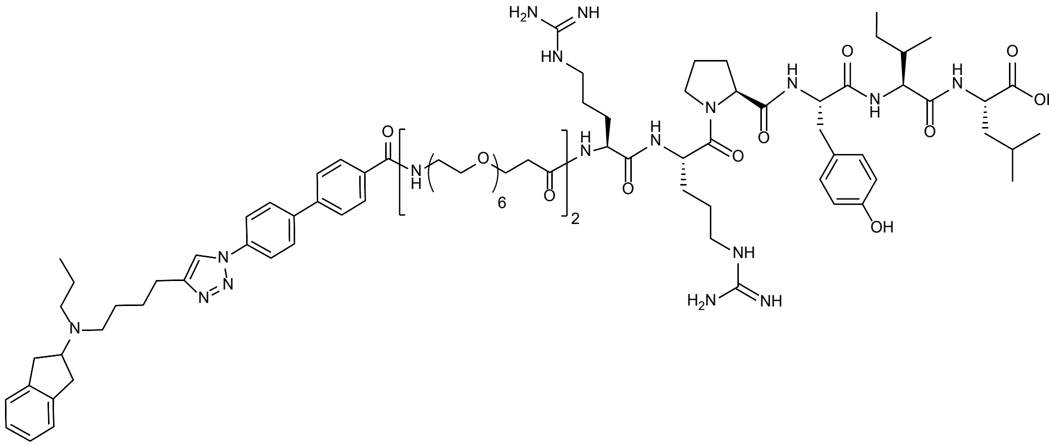

The heterobivalent ligand was synthesized according to method 1 using 2× Fmoc-NH-PEG$_6$-propionic acid and **21**. Purification: eluent: $CH_3CN$ (A) and 0.1 % HCOOH in $H_2O$ (B) applying a linear gradient starting from 5 % A in 95 % B to 42 % A in 58 % B in 15.0 min. FR: 32.0 mL min$^{-1}$. $t_R$: 13.9 min; purity: S1A: >99 % ($t_R$ = 22.9 min); S2A: >99 % ($t_R$ = 24.0 min); ESI-MS (*m/z*): 655.8 [(*M*+3H)/3]$^{3+}$; HRMS-ESI (*m/z*): [(*M*+2H)/2]$^{2+}$ calcd. for $C_{99}H_{154}N_{18}O_{23}$: 983.0805, found: 983.0807.

{1-[4'-(4-{4-[(2,3-dihydro-1H-inden-2-yl)(propyl)amino]butyl}-1H-1,2,3-triazol-1-yl)-(1,1'-biphenyl)-4-yl]-1-carboxamido-[tris-(4,7,10,13,16,19-hexaoxaheneicosanoylamido)]}-L-arginyl-L-arginyl-L-prolyl-L-tyrosyl-L-isoleucyl-L-leucine (**3c**)

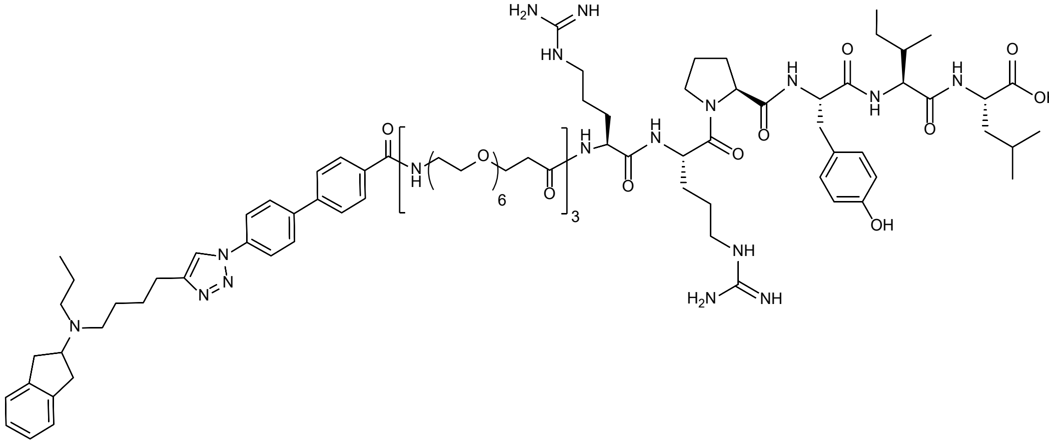

The heterobivalent ligand was synthesized according to method 1 using 3× Fmoc-NH-PEG$_6$-propionic acid and **21**. Purification: eluent: $CH_3CN$ (A) and 0.1 % HCOOH in $H_2O$ (B) applying a linear gradient

starting from 5 % A in 95 % B to 42 % A in 58 % B in 15.0 min. FR: 32.0 mL min$^{-1}$. $t_R$: 13.0 min; purity: S1B: 98 % ($t_R$ = 17.4 min); S2B: 98 % ($t_R$ = 18.6 min); ESI-MS (*m/z*): 768.5 [(*M*+3H)/3]$^{3+}$; HRMS-ESI (*m/z*): [(*M*+H]$^+$ calcd. for $C_{114}H_{183}N_{19}O_{30}$: 2300.3482, found: 2300.3407.

{1-[4'-(4-{4-[(2,3-dihydro-1H-inden-2-yl)(propyl)amino]butyl}-1H-1,2,3-triazol-1-yl)-(1,1'-biphenyl)-4-yl]-1-carboxamido-[tetrakis-(4,7,10,13,16,19-hexaoxaheneicosanoylamido)]}-L-arginyl-L-arginyl-L-prolyl-L-tyrosyl-L-isoleucyl-L-leucine (**3d**)

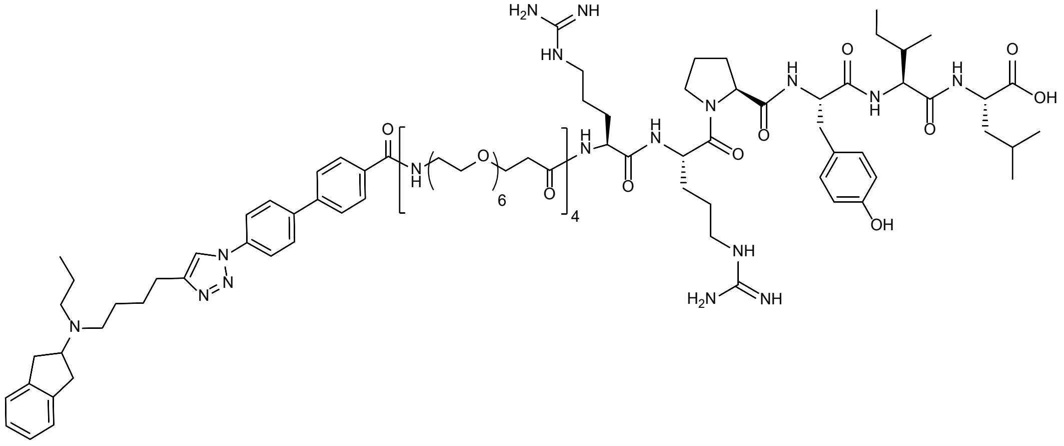

The heterobivalent ligand was synthesized according to method 1 using 4× Fmoc-NH-PEG$_6$-propionic acid and **21**. Purification: eluent: $CH_3CN$ (A) and 0.1 % HCOOH in $H_2O$ (B) applying a linear gradient starting from 5 % A in 95 % B to 42 % A in 58 % B in 15.0 min. FR: 32.0 mL min$^{-1}$. $t_R$: 13.0 min; purity: S1C: 98 % ($t_R$ = 18.5 min); S2C: 98 % ($t_R$ = 17.7 min); MS (ESI) *m/z* 659.8 [(*M*+4H)/4]$^{4+}$; HRMS-ESI *m/z* [*M*+H]$^+$ calcd. for $C_{129}H_{213}N_{20}O_{37}$: 2634.5395, found: 2634.5422.

{1-[4'-(4-{4-[(2,3-dihydro-1H-inden-2-yl)(propyl)amino]butyl}-1H-1,2,3-triazol-1-yl)-(1,1'-biphenyl)-4-yl]-1-carboxamido-(4,7,10,13,16,19-hexaoxaheneicosanoylamido)}-L-arginyl-L-arginyl-L-prolyl-*N*-[2-(4-hydroxyphenyl)ethyl]glycine-L-isoleucyl-L-leucine (**3e**)

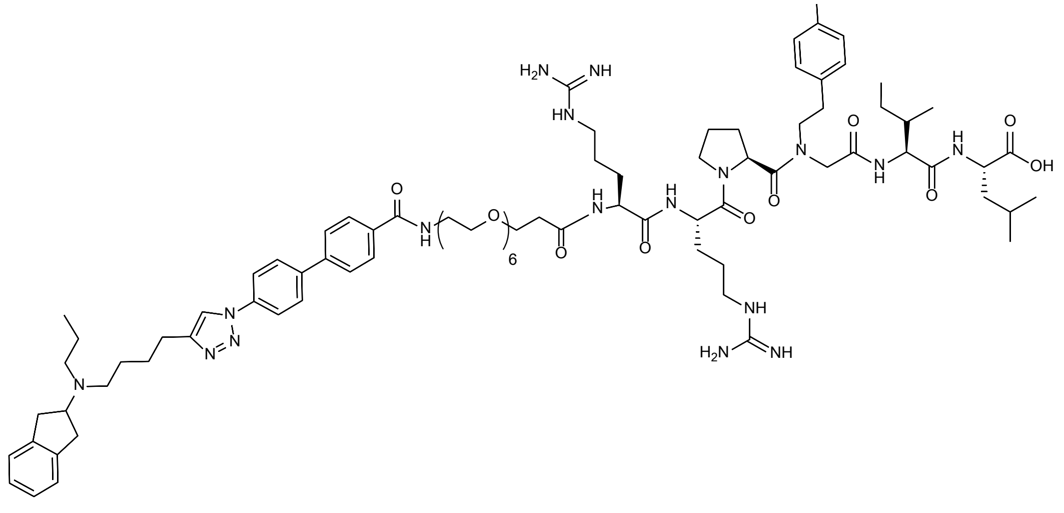

The heterobivalent ligand was synthesized according to method 1 using 1× Fmoc-NH-PEG$_6$-propionic acid and **21**. Purification: eluent: $CH_3CN$ (A) and 0.1 % HCOOH in $H_2O$ (B) applying a linear gradient starting from 5 % A in 95 % B to 42 % A in 58 % B in 15.0 min. FR: 32.0 mL min$^{-1}$. $t_R$: 14.0 min; purity: S1A: >99 % ($t_R$ = 22.8 min); S2A: >99 % ($t_R$ = 22.7 min); ESI-MS (*m/z*): 548.6 [(*M*+3H)/3]$^{3+}$; HRMS-ESI (*m/z*): [(*M*+3H)/3]$^{3+}$ calcd. for $C_{84}H_{127}N_{17}O_{16}$: 548.6632, found: 548.6636.

{1-[4'-(4-{4-[(2,3-dihydro-1H-inden-2-yl)(propyl)amino]butyl}-1H-1,2,3-triazol-1-yl)-(1,1'-biphenyl)-4-yl]-1-carboxamido-[bis-(4,7,10,13,16,19-hexaoxaheneicosanoylamido)]}-L-arginyl-L-arginyl-L-prolyl-*N*-[2-(4-hydroxyphenyl)ethyl]glycine-L-isoleucyl-L-leucine (**3f**)

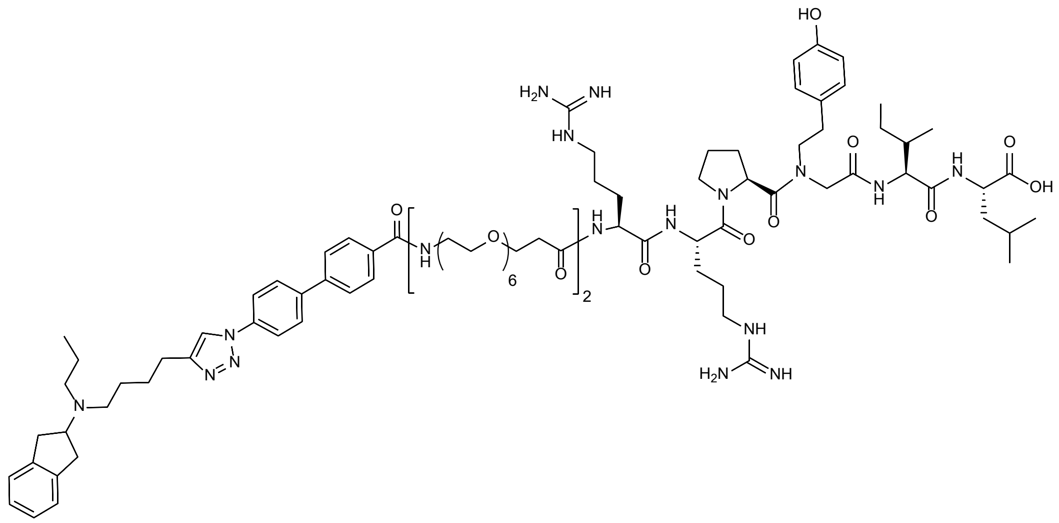

The heterobivalent ligand was synthesized according to method 1 using 2× Fmoc-NH-PEG$_6$-propionic acid and **21**. Purification: eluent: $CH_3CN$ (A) and 0.1 % HCOOH in $H_2O$ (B) applying a linear gradient starting from 5 % A in 95 % B to 42 % A in 58 % B in 15.0 min. FR: 32.0 mL min$^{-1}$. $t_R$: 14.0 min; purity: S1A: >99 % ($t_R$ = 23.1 min); S2A: >99 % ($t_R$ = 24.0 min); ESI-MS (*m/z*): 660.5 [(*M*+3H)/3]$^{3+}$; HRMS-ESI (*m/z*): [(*M*+Na+2H)/3]$^{3+}$ calcd. for $C_{100}H_{156}N_{18}O_{23}$: 667.7178 found: 667.7243.

## Supplementary References

1. Hiller C., Kling R. C., Heinemann F. W., Meyer K., Hübner H., Gmeiner P. Functionally selective dopamine D2/D3 receptor agonists comprising an enyne moiety. *J. Med. Chem.* **56,** 5130-5141 (2013).
2. de Paulis T., Hall H., Ögren S.-O., Wägner A., Stensland B., Csöregh I. Synthesis, crystal structure and antidopaminergic properties of eticlopride (FLB 131). *Eur. J. Med. Chem.* **20,** 273-276 (1985).
3. Guo W., Shi L., Javitch J. A. The fourth transmembrane segment forms the interface of the dopamine D2 receptor homodimer. *J. Biol. Chem.* **278,** 4385-4388 (2003).
4. Lee S. P., O'Dowd B. F., Rajaram R. D., Nguyen T., George S. R. D2 Dopamine Receptor Homodimerization Is Mediated by Multiple Sites of Interaction, Including an Intermolecular Interaction Involving Transmembrane Domain 4. *Biochemistry* **42,** 11023-11031 (2003).
5. Guo W., Shi L., Filizola M., Weinstein H., Javitch J. A. Crosstalk in G protein-coupled receptors: changes at the transmembrane homodimer interface determine activation. *Proc. Natl. Acad. Sci. U.S.A.* **102,** 17495-17500 (2005).
6. Guo W*., et al.* Dopamine D2 receptors form higher order oligomers at physiological expression levels. *EMBO J.* **27,** 2293-2304 (2008).
7. Kühhorn J., Götz A., Hübner H., Thompson D., Whistler J., Gmeiner P. Development of a bivalent dopamine D(2) receptor agonist. *J. Med. Chem.* **54,** 7911-7919 (2011).
8. Ciruela F*., et al.* Combining Mass Spectrometry and Pull-Down Techniques for the Study of Receptor Heteromerization. Direct Epitope-Epitope Electrostatic Interactions between Adenosine A2A and Dopamine D2 Receptors. *J. Anal. Chem.* **76,** 5354-5363 (2004).
9. Borroto-Escuela D. O*., et al.* A serine point mutation in the adenosine A2AR C-terminal tail reduces receptor heteromerization and allosteric modulation of the dopamine D2R. *Biochem. Biophys. Res. Commun.* **394,** 222-227 (2010).
10. Lukasiewicz S., Polit A., Kedracka-Krok S., Wedzony K., Mackowiak M., Dziedzicka-Wasylewska M. Hetero-dimerization of serotonin 5-HT(2A) and dopamine D(2) receptors. *Biochim. Biophys. Acta* **1803,** 1347-1358 (2010).
11. Borroto-Escuela D. O*., et al.* Characterization of the A2AR-D2R interface: focus on the role of the C-terminal tail and the transmembrane helices. *Biochem. Biophys. Res. Commun.* **402,** 801-807 (2010).
12. Pei L*., et al.* Uncoupling the dopamine D1-D2 receptor complex exerts antidepressant-like effects. *Nat. Med.* **16,** 1393-1395 (2010).
13. O'Dowd B. F., Ji X., Nguyen T., George S. R. Two amino acids in each of D1 and D2 dopamine receptor cytoplasmic regions are involved in D1-D2 heteromer formation. *Biochem. Biophys. Res. Commun.* **417,** 23-28 (2012).
14. O'Dowd B. F., Nguyen T., Ji X., George S. R. D5 dopamine receptor carboxyl tail involved in D5-D2 heteromer formation. *Biochem. Biophys. Res. Commun.* **431,** 586-589 (2013).

15. Hasbi A., *et al.* A peptide targeting an interaction interface disrupts the dopamine D1-D2 receptor heteromer to block signaling and function in vitro and in vivo: effective selective antagonism. *FASEB J.*, 1-15 (2014).

16. Egloff P., *et al.* Structure of signaling-competent neurotensin receptor 1 obtained by directed evolution in Escherichia coli. *Proc. Natl. Acad. Sci. U.S.A.* **111,** E655-E662 (2014).

17. Huang J., Chen S., Zhang J. J., Huang X.-Y. Crystal structure of oligomeric β1-adrenergic G protein–coupled receptors in ligand-free basal state. *Nat. Struct. Mol. Biol.* **20,** 419-425 (2013).

18. Dörfler M., Tschammer N., Hamperl K., Hübner H., Gmeiner P. Novel D3 selective dopaminergics incorporating enyne units as nonaromatic catechol bioisosteres: synthesis, bioactivity, and mutagenesis studies. *J. Med. Chem.* **51,** 6829-6838 (2008).

19. Tschammer N., *et al.* Highly Potent 5-Aminotetrahydropyrazolopyridines: Enantioselective Dopamine D-3 Receptor Binding, Functional Selectivity, and Analysis of Receptor-Ligand Interactions. *J. Med. Chem.* **54,** 2477-2491 (2011).

20. Schaab C., *et al.* Structure-Based Evolution of Subtype-Selective Neurotensin Receptor Ligands. *ChemistryOpen* **3,** 206-218 (2014).

21. Hornak V., Abel R., Okur A., Strockbine B., Roitberg A., Simmerling C. Comparison of multiple Amber force fields and development of improved protein backbone parameters. *Proteins: Struct. Funct. Bioinform.* **65,** 712-725 (2006).

22. Wang J., Wolf R. M., Caldwell J. W., Kollman P. A., Case D. A. Development and testing of a general amber force field. *J. Comput. Chem.* **25,** 1157-1174 (2004).

23. Frisch M. J., *et al.* Gaussian 09, Revision B.01. Gaussian, Inc.: Wallingford, CT (2009).

24. Bayly C. I., Cieplak P., Cornell W., Kollman P. A. A well-behaved electrostatic potential based method using charge restraints for deriving atomic charges: the RESP model. *J. Phys. Chem.* **97,** 10269-10280 (1993).

25. Clark T., Hennemann M., Murray J. S., Politzer P. Halogen bonding: the σ-hole. *J. Mol. Model.* **13,** 291-296 (2007).

26. Politzer P., Murray J. S., Clark T. Halogen bonding: an electrostatically-driven highly directional noncovalent interaction. *Phys. Chem. Chem. Phys.* **12,** 7748-7757 (2010).

27. Rendine S., Pieraccini S., Forni A., Sironi M. Halogen bonding in ligand-receptor systems in the framework of classical force fields. *Phys. Chem. Chem. Phys.* **13,** 19508-19516 (2011).

28. Kolář M., Hobza P. On Extension of the Current Biomolecular Empirical Force Field for the Description of Halogen Bonds. *J. Chem. Theory Comput.* **8,** 1325-1333 (2012).

29. Jorgensen W. L., Schyman P. Treatment of Halogen Bonding in the OPLS-AA Force Field: Application to Potent Anti-HIV Agents. *J. Chem. Theory Comput.* **8,** 3895-3901 (2012).

30. Van Der Spoel D., Lindahl E., Hess B., Groenhof G., Mark A. E., Berendsen H. J. GROMACS: fast, flexible, and free. *J. Comput. Chem.* **26,** 1701-1718 (2005).

31. Hess B., Kutzner C., van der Spoel D., Lindahl E. GROMACS 4: Algorithms for highly efficient, load-balanced, and scalable molecular simulation. *J. Chem. Theory Comput.* **4,** 435-447 (2008).

32. Wolf M. G., Hoefling M., Aponte-Santamaría C., Grubmüller H., Groenhof G. g_membed: Efficient insertion of a membrane protein into an equilibrated lipid bilayer with minimal perturbation. *J. Comput. Chem.* **31,** 2169-2174 (2010).

33. Siu S. W., Vácha R., Jungwirth P., Böckmann R. A. Biomolecular simulations of membranes: physical properties from different force fields. *J. Chem. Phys.* **128,** 125103 (2008).

34. Goetz A., Lanig H., Gmeiner P., Clark T. Molecular dynamics simulations of the effect of the G-protein and diffusible ligands on the β2-adrenergic receptor. *J. Mol. Biol.* **414,** 611-623 (2011).

35. Berendsen H., Grigera J., Straatsma T. The missing term in effective pair potentials. *J. Phys. Chem.* **91,** 6269-6271 (1987).

36. Pettersen E. F.*, et al.* UCSF Chimera—A visualization system for exploratory research and analysis. *J. Comput. Chem.* **25,** 1605-1612 (2004).

37. Hübner H., Haubmann C., Utz W., Gmeiner P. Conjugated enynes as nonaromatic catechol bioisosteres: Synthesis, binding experiments and computational studies of novel dopamine receptor agonists recognizing preferentially the D3 subtype. *J. Med. Chem.* **43,** 756-762 (2000).

38. Hayes G., Biden T. J., Selbie L. A., Shine J. Structural subtypes of the dopamine D2 receptor are functionally distinct: expression of the cloned D2A and D2B subtypes in a heterologous cell line. *Mol. Endocrinol.* **6,** 920-926 (1992).

39. Sokoloff P., Giros B., Martres M.-P., Bouthenet M.-L., Schwartz J.-C. Molecular cloning and characterization of a novel dopamine receptor (D3) as a target for neuroleptics. *Nature* **347,** 146-151 (1990).

40. Asghari V., Sanyal S., Buchwaldt S., Paterson A., Jovanovic V., Van Tol H. H. M. Modulation of intracellular cyclic AMP levels by different human dopamine D4 receptor variants. *J. Neurochem.* **65,** 1157-1165 (1995).

41. Jordan M., Schallhorn A., Wurm F. M. Transfecting mammalian cells: optimization of critical parameters affecting calcium-phosphate precipitate formation. *Nucleic Acids Res.* **24,** 596-601 (1996).

42. Lowry O. H., Rosebrough N. J., Farr A. L., Randall R. J. Protein measurement with the folin phenol reagent. *J. Biol. Chem.* **193,** 265-275 (1951).

43. Einsiedel J.*, et al.* Discovery of highly potent and neurotensin receptor 2 selective neurotensin mimetics. *J. Med. Chem.* **54,** 2915-2923 (2011).

44. Watanabe K., Fukumura T., Sasaki S., Maeda M., Takehara S. New Fluorine-Substituted Analogue of Eticlopride with High Affinity toward Dopamine D2 Receptors. *Chem. Pharm. Bull. (Tokyo)* **39,** 3211-3214 (1991).

45. Westmark P. R., Gardiner S. J., Smith B. D. Selective monosaccharide transport through lipid bilayers using boronic acid carriers. *J. Am. Chem. Soc.* **118,** 11093-11100 (1996).
